# The magnitude of sex differences in verbal episodic memory increases with social progress: Data from 54 countries across 40 years

**DOI:** 10.1371/journal.pone.0214945

**Published:** 2019-04-22

**Authors:** Martin Asperholm, Sanket Nagar, Serhiy Dekhtyar, Agneta Herlitz

**Affiliations:** 1 Division of Psychology, Department of Clinical Neuroscience, Karolinska Institutet, Stockholm, Sweden; 2 Monash Institute of Cognitive and Clinical Neuroscience, School of Psychological Science, Monash University, Melbourne, Australia; 3 Aging Research Center, Department of Neurobiology, Care Sciences and Society, Karolinska Institutet, Stockholm, Sweden; Nathan S Kline Institute, UNITED STATES

## Abstract

Sex differences in episodic memory have been reported. We investigate (1) the existence of sex differences in verbal and other episodic memory tasks in 54 countries, and (2) the association between the time- and country-specific social progress indicators (a) female to male ratio in education and labor force participation, (b) population education and employment, and (c) GDP per capita, and magnitude of sex differences in verbal episodic memory tasks. Data were retrieved from 612 studies, published 1973–2013. Results showed that females outperformed (Cohen’s *d* > 0) males in verbal (42 out of 45 countries) and other (28 out of 45 countries) episodic memory tasks. Although all three social progress indicators were, separately, positively associated with the female advantage in verbal episodic memory performance, only population education and employment remained significant when considering the social indicators together. Results suggest that women’s verbal episodic memory performance benefits more than men’s from education and employment.

## Introduction

The presence of sex differences in some but not all cognitive functions is well documented [1]. For example, women are often reported to have an advantage in verbal production [2], reading comprehension [3], and episodic memory (recollection of past experiences in terms of their content, location, and temporal occurrence; [4, 5]), while men often perform at a higher level on visuospatial [6, 7] and some numerical tasks [8–10]. These sex differences are typically modest in size and the underlying mechanisms are not yet sufficiently understood. Despite this, they have consistently been reported from early childhood into old age, and in most examined regions of the world, although their magnitude seems to vary across countries [3, 5, 8, 10–14]. Here, factors contributing to the variation in magnitude of sex differences in episodic memory will be examined.

Along with the constant progression in economic, political, environmental, and social landscapes across the globe [15, 16], there is evidence suggesting that cognitive performance is improving. The *Flynn effect*, describing intergenerational gains in IQ scores, has been documented in almost every region where it has been examined [17]. Some of the factors contributing to these cognitive gains across generations are increased education, shifts in family size, and strengthened health care [18]. The *Flynn effect* is typically larger in regions that have experienced the most rapid pace of development over the examined time period [19] and women are reported to exhibit a larger *Flynn effect* than men [19].

The gender equality of a country often refers to the degree to which women, relative to men, have access to education, economic opportunity, and political resources and may thereby be an indicator of the living conditions of women. Several composite and domain-specific nation-level indicators of gender equality exist, focusing to a varying degree on political resources, economic opportunities, education, and health [20]. Based on findings of the origin of the *Flynn effect*, the association between indicators of gender equality and sex differences in cognitive performance is of interest.

The associations between gender equality indicators and cognitive sex differences have been investigated using the PISA database, where data on mathematics performance and reading comprehension in 15-16-year-olds is available between the years 2000 and 2009 (OECD-PISA). For example, Guiso and colleagues [3] examined the 2003 PISA wave and reported associations between the Gender Gap Index (GGI; [21]) on the one hand, and mathematical performance on the other, with boys´ overall advantage disappearing in the most gender equal countries. Else-Quest and colleagues [8] examined other indicators of gender equality and mathematics performance in the same 2003 PISA wave and for Trends in International Mathematics and Science Study (IEA-TIMSS). Associations were found between gender equality in girls´ school enrollment and TIMSS, and between other indicators of gender equality and sex differences in mathematic performance in PISA data. In contrast, Stoet and Geary [22] reported that no consistent pattern could be found when analyzing all four PISA waves in relation to GGI and other gender equality indicators from United Nations Development Project (UNDP). Also, Lippa and colleagues [12] investigated mental rotation and line angle judgment performance in the BBC internet survey of human sex differences [23] and found a relationship between these measures and gender equality going in the opposite direction, with larger sex differences favoring men in more gender equal societies.

Relatively few studies have been conducted on sex differences in those cognitive areas in which there is a general female advantage. However, a larger female advantage in the PISA reading comprehension test has been found in more gender equal societies [3, 24], although not systematically [22]. Using data from Survey of Health, Ageing and Retirement in Europe (SHARE), Weber [10] reported that having experienced better living conditions in younger adulthood is associated with larger sex differences favoring women in a verbal episodic memory task across 13 European countries in individuals 50 years and older. Similarly, Bonsang and colleagues [25], also using data from SHARE and databases from other international studies, found that educational attainment, labor-market participation and societal gender norms favoring women’s participation in the work force were positively related to a female advantage in the same verbal episodic memory task assessed in 27 countries.

Taken together, findings on the association between social progress indicators and sex differences in cognitive tasks are mixed. Furthermore, most research has been conducted on the same databases, thereby limiting the generalizability of the research to sex differences in mathematics, reading comprehension, and a specific verbal episodic memory task. In contrast, we used 612 individual studies published 1973–2013, originating from 54 countries to, first, examine the existence of sex differences across countries in both verbal and other episodic memory tasks. Because recent research [3, 10, 12, 25] has demonstrated that similar patterns of cognitive sex differences exist across nations, we expected to find sex differences favoring females in most countries. Our second goal was to explain potential cross-national variability in these sex differences, exploring whether time-specific national indicators of gender equality, education level and labor force participation, and economic development are associated with cross-national variations of sex differences in episodic memory. We hypothesized that these national and time-specific indicators would be positively correlated with a relative female advantage in episodic memory and that the indicator of gender equality would be the strongest predictor.

## Methods

### Dataset

A database of effect sizes—standardized differences in episodic memory performance between men and women—was obtained from a meta-analysis investigating sex differences in episodic memory [5]. In total, the database consisted of 612 studies originating from 54 different countries, published between 1973 and 2013, involving a total of 587,691 participants. Most of the articles included had several samples (e.g., age groups), participating in more than one episodic memory task (e.g., list of word and list of faces), some with more than one dependent measure (e.g., free and cued recall of a word list, and immediate and delayed recognition of faces).

All studies included in the database fulfilled the following criteria: (1) the study contained original, empirical data and was published in a peer-reviewed journal available in English; (2) the sample consisted of males and females who were not selected based on based on any diagnosis, disease, or disorder and not influenced in any way that may have affected their episodic memory performance; (3) episodic memory was tested using controlled and uniform tasks for all participants within the sample; and (4) the dependent measure assessed episodic memory performance (Asperholm et al., 2018). For more information about the dataset, see Asperholm and colleagues [5].

### Episodic memory tasks

The episodic memory tasks were originally sorted into eight categories based on the material to be remembered [5]. Here, we analyzed the dataset *Verbal*, in which the episodic memory tasks consisted of words, sentences, facts, conversations, or nameable objects to be remembered. This category contained 495 studies originating from 45 countries. The other seven categories, *Images*, *Faces*, *Movies*, *Locations*, *Routes*, *Sensory*, *and Remaining* were not analyzed separately because they originated from a limited number of countries (*Movies* k = 7; *Routes* k = 9; *Faces* k = 12; *Sensory* k = 4), had social indicator distributions which negated useful analyses (*Images*; *Locations*), or had material which was unknown or could not be categorized into any of the categories (*Remaining*). Instead, these categories were combined into the dataset *Other* episodic memory tasks, thus, containing all effect sizes but those included in *Verbal*. The *Other* category contained 373 studies originating from 45 countries.

### Country and year

For both datasets, *Verbal* and *Other*, if information regarding the country where the study was conducted was not explicitly stated in the article, it was derived from the affiliation(s) of the (first) author(s). Studies originating from countries from which predictors could not be retrieved were excluded, resulting in 54 countries being represented in the final data set. Since exact dates for data collection were not available for most studies, dates were estimated by subtracting 1 from year of publication. Thus, the data collections forming the basis of the included studies were assumed to have taken place between 1972 and 2013.

### Social progress indicators

Three indicators of a country’s living conditions were assessed for their association with the magnitude of sex differences in episodic memory performance. As the studies used in the analyses had been conducted at different time-points, spanning from 1972 to 2013, the indicators needed to be time-specific as well as country-specific. The indicators, described below, were retrieved from the World Bank (WB; [26]) and UNDP [27].

#### Gender equality

This is a composite measure that comprises (1) the UNDP indicator Average Years of Schooling Attained (Female population, 25 years and over) subtracted by Average Years of Schooling Attained (Male population, 25 years and over) and (2) the WB indicator Female to male ratio in labor force participation (for ages between 15 and 64). All data points were z-transformed using the mean and standard deviation for all possible year/country combinations of the included countries throughout the relevant years 1972–2013. This composite measure was assumed to be indicative of national gender equality in educational and occupational opportunities from 1972 to 2013.

#### Population education and employment

This is a composite measure that comprises (1) the UNDP indicator *Average Years of Schooling Attained (Total population*, *25 years and over)* and (2) the WB indicator *Labor force participation rate* (% of total population ages 15–64). All data points were z-transformed using the mean and standard deviation for all possible year/country combinations for the included countries throughout the relevant years 1972–2013. This composite measure was assumed to be indicative of the national education level and labor force participation from 1972 to 2013.

#### GDP per capita

Gross domestic product (GDP) per capita is a measure of economic activity in relation to the size of the population. This measure, given in current US dollars, was extracted from the WB database and subsequently transformed using the natural logarithm to achieve a normal distribution of the variable. All data points were z-transformed using the mean and standard deviation for all possible year/country combinations of the included countries throughout the relevant years 1972–2013.

#### Data extrapolation

The indicators of *Average Years of Schooling Attained* (UNDP) were available in 5-year intervals. Indicators of *Labor force participation* (WB) had a more consistent coverage but were only available from 1990 and onwards, while *GDP per Capita* was available for the complete time-range. If an indicator value was missing for a certain year and country combination, linear extrapolation was undertaken using the closest available data points before and after the year in question. If only a later or prior data point was available, the value of that data point was used. For the full dataset, this resulted in estimations of 43% of the values for *Gender Equality*, 43% for *Population Education and Employment*, and 0% for *GDP per Capita*.

A table of the studies, effect sizes, and social progress indicators included in the analyses, can be found in Supporting information (S1 Table), together with a list of references for those studies (S1 References).

### Statistical analysis

Sex differences were specified using Cohen’s *d* ((*m*_women_−*m*_men_)/ *sd*_total_), with a positive effect size indicating an advantage for females in episodic memory and a negative effect size indicating an advantage for males. Analyses of publication bias has previously been reported, which showed that the sex differences in episodic memory did not result from a bias to study or report sex differences, or from using selective samples [5].

In order to determine the presence as well as the extent of sex differences in *Verbal* and *Other* across countries, two five-level random-effects meta-analytic linear models with country as a discrete moderator were performed (accounting for the hierarchical structure of the data with studies, samples and tasks described under *Dataset*).

The next step involved examining the association between living conditions (*Gender Equality*, *Population Education and Employment*, and *GDP per Capita*) and sex differences in episodic memory for *Verbal* and *Other* across countries over the period 1972–2013. First, we computed the correlations (Pearson’s *r*) between the social progress indicators alone, followed by separate six-level meta-regression models (adding country as a hierarchical level) assessing the direct effects of each measure on sex differences in *Verbal* episodic memory tasks. The final part of the analysis involved fitting a meta-regression that included all three indicators, allowing us to assess the independent contributions of *Gender Equality*, *Population Education and Employment*, and *GDP per Capita* to the variation in sex differences in *Verbal*. A power analysis to calculate sample size was not computed beforehand as sample size was determined by available studies [5].

All analyses above were performed using version 1.9–9 of the R package *metafor* [28].

## Results

**Fig 1:**
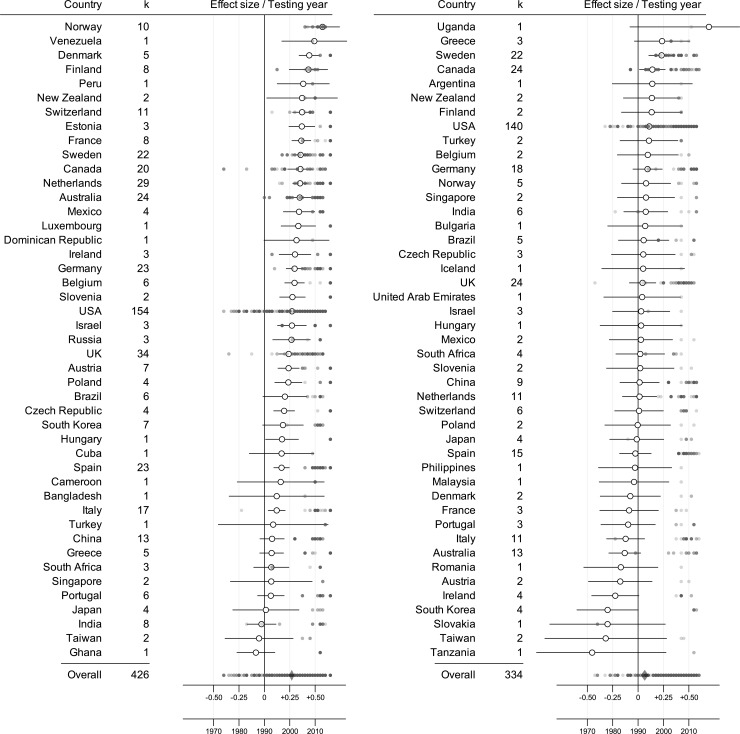
Effect size estimates for each country for both task categories. Forest plot describing the variation in sex differences in (a) Verbal and (b) Other episodic memory tasks across the (a) 45 and (b) 45 countries used in the analyses, with k = number of studies available for each country. Cohen’s *d* (unfilled circles) is presented on the x-axis, with error bars describing the 95% confidence intervals (notice that some error bars have been truncated). Filled circles indicate the year each study was carried out.

Two separate five-level random effects meta-analyses with country as moderator were computed for both the datasets *Verbal* (Fig 1A) and *Other* (Fig 1B) to assess the overall pattern of sex differences and variation in magnitude of sex differences across countries. For *Verbal* episodic memory tasks, there was an overall female advantage (*d* = .27; CI = [.24, .30]; p < .001) that was seen in most countries (i.e., Cohen’s *d* > 0 in 42 out of 45 countries), although the sex difference was not statistically significant in all of them. The moderator analysis showed a statistical significant impact of country (QM (df = 44) = 185; p < .001), justifying subsequent analyses trying to explain this variation.

For *Other* episodic memory tasks, there was an overall small female advantage (*d* = .07; CI = [.03, .10]; p < .001), seen in approximately four-fifths of the countries (i.e., Cohen’s *d* > 0 in 28 out of 45 countries), but again not statistically significant in all of them. The moderator analysis showed no statistical significant impact of country (QM (df = 44) = 52; p = .18), thereby not justifying analyses of variation across countries.

Correlation analyses for the full dataset revealed positive associations between the three indicators (*Gender Equality* vs. *Population Education and Employment*: *r* = .70, *p* < .001; *Gender Equality* vs. *GDP per Capita*: *r* = .50, *p* < .001*; Population Education and Employment* vs. *GDP per Capita*: *r* = .56, *p* < .001).

**Fig 2:**
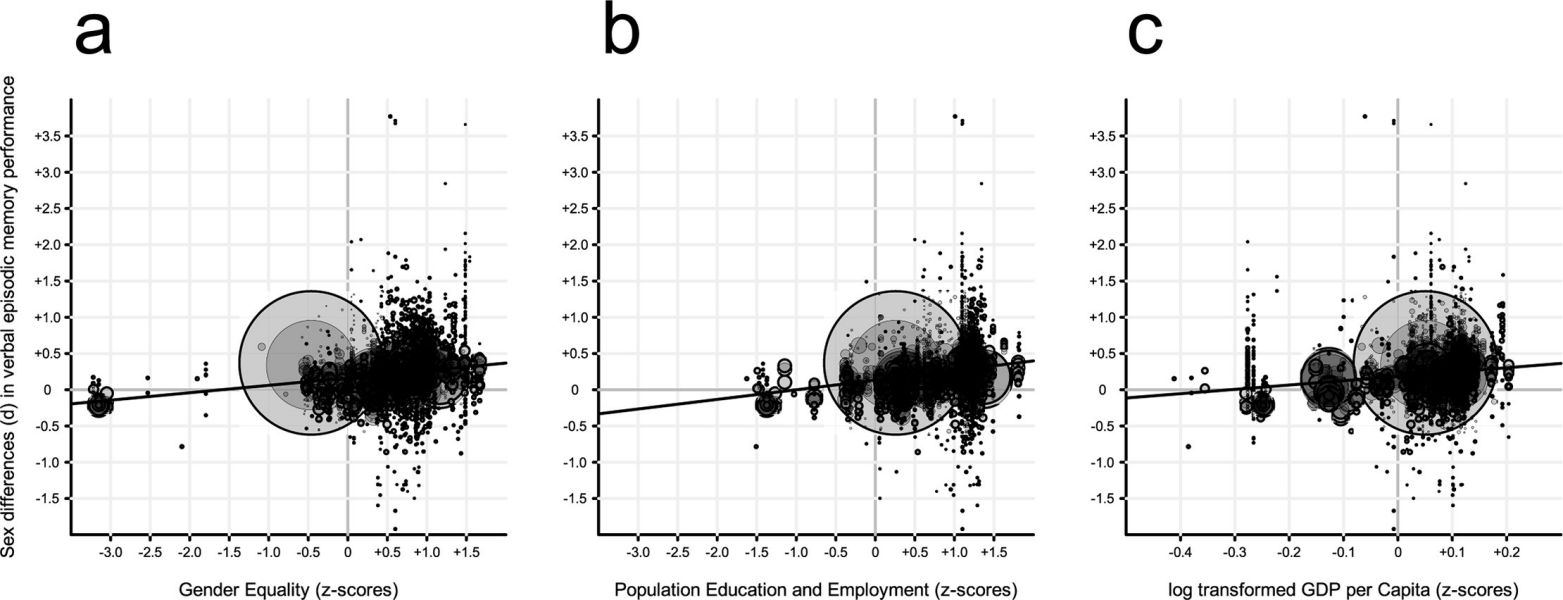
Scatterplots for the three simple effects analyses. Indicator of (a) Gender equality, (b) Population Education and Employment, (c) GDP per Capita (x-axis) plotted against sex differences in Verbal episodic memory performance (y-axis). The diameter of each data point is equal to the inverse of its squared variance. The lines indicate the best-fitting regressions.

Simple and combined models estimating direct unadjusted effects of the indicator variables *Gender Equality*, *Population Education and Employment*, and *GDP per Capita* were computed for *Verbal* episodic memory tasks (for summary statistics, see Table 1; for scatterplots of the simple effects, see Fig 2). For *Verbal*, the estimated B-values in all simple models were positive and statistically significant, whereas for the combined model, only *Population Education and Employment* remained significant. Because the indicator variables were transformed to z-values before they were analyzed, the estimated B-values for these indicators specify how much the magnitude of the sex difference in *Verbal* episodic memory changes per change in z-point for the indicator. Thus, in the combined model, one z-point change in *Population Education and Employment* is associated with an increase of 0.08 in Cohen’s *d* in *Verbal* episodic memory performance.

**Table 1:** Summary statistics of regression analyses for *Verbal* episodic memory task.

	Simple model 1	Simple model 2	Simple model 3	Combined model
**Intercept**				
** Estimate**	0.16***	0.13***	0.18***	0.14***
** Standard error**	0.02	0.02	0.02	0.02
**Gender Equality**				
** B estimate**	0.10			0.04
** B standard error**	0.02			0.03
**Population Education and Employment**				
** B estimate**		0.13***		0.08*
** B standard error**		0.02		0.04
**GDP per Capita**				
** B estimate**			0.60***	0.18
** B standard error**			0.16	0.20
**Observations**	2681	2681	2681	2681
**R**^**2**^	0.12	0.15	0.11	0.16

* p < .05

** p < .01

*** p < .001

Meta-regressions were also computed for *Verbal* episodic memory tasks excluding the large databases used by Weber et al. [10] and Bonsang et al. [25], leaving the 419 studies collected by Asperholm and colleagues [5]. Excluding these databases did not change the overall pattern of results except that Population Education and Employment went from significant to marginally significant in the combined model (see S2 Table). Similarly, analyzing only the databases used by Weber et al. [10] and Bonsang et al. [25] yielded a similar pattern of results, the only difference between this analysis and the original for *Verbal* being that the estimated B-value for *GDP per capita* in the combined model became significant (see S2 Table).

## Discussion

The aims of this study were (1) to determine the existence and magnitude of sex differences in episodic memory tasks across countries, and (2) to investigate if and how societal changes—in terms of time-specific national indicators of gender equality, educational level and labor force participation, and economic development—were associated with the magnitude of sex differences in episodic memory. These questions were addressed in a large dataset, including 612 studies originating from 54 countries, spanning over 40 years. In line with our hypothesis, results showed that there was an overall female advantage for *Verbal* episodic memory, found in 42 of 45 countries although not reliably so in all. *Other* episodic memory tasks also yielded an advantage for women, although smaller, found in 28 of 45 countries. Furthermore, results indicated that all three social progress indicators were, independently, positively associated with the female advantage in *Verbal* episodic memory performance. *Population Education and Employment* was the most important predictor among the three indicators for *Verbal* episodic memory, contrary to our hypothesis that gender equality would be the strongest predictor.

Although the female advantage in Verbal episodic memory tasks was seen in most countries, the advantage was not reliable in all of them (see Fig 1A). One reason for the lack of significance may be that too few studies were conducted in some countries providing a less reliable estimate of the sex difference. The finding of a general female advantage in *Verbal* episodic memory is in line with other studies reporting an advantage for women in most countries [10, 14, 25]. It is also in line with other research, reporting remarkably similar patterns of sex differences in reading comprehension, mathematics, mental rotation, and line angle judgment across a large number of countries (e.g., [11, 12]). Our results extend previous findings of similarities across nations in regard to episodic memory, not only in that more countries and studies were included, but also in that the female advantage in episodic memory can be generalized to a multitude of verbal episodic memory tasks, and does not only hold for the specific word list analyzed in previous studies [10, 25].

By linking country- and time specific social indicators to each of the studies included in our analysis, we found that the indicators *Gender equality*, *Population Education and Employment*, and *GDP per Capita* were reliable predictors of the female advantage when analyzed separately. However, the only predictor of a female *Verbal* episodic memory advantage when taking all indicators into account was *Population Education and Employment*, an indicator of education level and labor force participation in the population. There is substantial literature showing that educational attainment is positively associated with cognitive performance, with recent evidence from quasi-experimental studies demonstrating that prolonging compulsory education by one or two years may increase cognitive performance [29, 30]. We should therefore expect an indicator of a country’s education level to be predictive of the country’s cognitive performance level in general. Our results suggest, however, that women gain cognitively more than men from higher educational and occupational participation, as the female advantage is larger in countries with higher population education and occupation. This result both confirms and extends previous findings [10, 19, 25] by showing that societal improvements in education and labor force participation are associated with a larger female advantage in a multitude of verbal episodic memory tasks.

The indicator aimed to capture *Gender Equality*, namely the difference between women’s and men’s educational level and labor force participation, and *GDP per Capita* were both highly correlated with *Population Education and Employment*. It is therefore likely that *Population Education and Employment* captures some of the positive effects estimated in the two other indicators, thereby explaining why these indicators were relatively less predictive of the sex difference in *Verbal* episodic memory.

The sex difference in *Other* episodic memory tasks was small. This is not surprising as the categories constituting *Other* episodic memory tasks to a varying degree yield sex differences, with a female (*Movies*, *Locations*, *Faces*, *Sensory*, *Remaining*), male (*Routes*) or no (*Images*) advantage [5]. As the analysis of cross-national variability in the sex differences in *Other* episodic memory tasks came out insignificant, analyses attempting to explain variation across countries were redundant.

### Strengths and limitations

Although the current study has a number of strengths, such as assessing sex differences in a large number of countries sampled over a 40-year period, it also has some limitations. The most obvious limitation is that cause and effect inferences cannot be made; the results only suggest an association between the social indicators and the magnitude of the difference. Second, the majority of research on episodic memory is conducted in North America, Europe, and Australia, whereas few studies have been conducted in African, Asian, and South American countries. The estimates of the magnitude of the sex differences are therefore less reliable in the latter. Lastly, the current analyses comprised studies that used a variety of verbal episodic memory tasks. While this heterogeneity may act as a limitation, it also forms the basis of a very conservative test of the hypotheses.

## Conclusions

The results of the current study indicate that the female advantage in episodic memory is present in most countries. The results also suggest that the advantage in verbal episodic memory tasks is further heightened in countries with higher population education and labor force participation, likely serving as proxies for increased education, heightened cognitive stimulation, and a more enriching environment. Although it is still an open question why women seem to be more positively affected by these societal improvements than men, it can be hypothesized that women benefit disproportionately because they may start from a more disadvantaged level. More research into these questions is warranted.

## Supporting information

S1 Table Details of all studies used in the analyses. Studies included in the database with country of study, effect size *d* (variance) of *Verbal* and *Other* episodic memory tasks, total number of males/females participating in the study, together with country- and time-specific measures indicative of gender equality in educational and occupational opportunities, population education level and labor force participation, and GDP per capita. Effect sizes were derived by using the formulas recommended by Borenstein et al. [31] to combine intersample and intrasample data points. For more information about this procedure, see Asperholm et al. (5). Explanation of headings: Country = Country of study; *d* (variance) = Cohen’s *d* and variance of categories *Verbal* and *Other* episodic memory tasks (when combining all effect sizes within a study using verbal material or other, non-verbal, material); N Males/Females = The total number of males/females participating in the study; Gender equality = A country- and time-specific measure indicative of gender equality in educational and occupational opportunities; Population Education Employment = A country- and time-specific measure indicative of education level and labor force participation; GDP per Capita = A country- and time-specific measure of economic activity in relation to the size of the population.
(PDF)

S2 Table Summary table of additional sensitivity regression analyses. Summary table of regression analyses for *Verbal* excluding data from databases used by Weber et al. [10] and Bonsang et al. [25], and for *Verbal* only containing data used by Weber et al. [10] and Bonsang et al. [25]. (*) p < .10; * *p* < .05; ** *p* < .01; *** *p* < .001.
(PDF)

S1 References References of studies included in the analyses. (PDF)
