# Correction: The magnitude of sex differences in verbal episodic memory increases with social progress: Data from 54 countries across 40 years

**DOI:** 10.1371/journal.pone.0217033

**Published:** 2019-05-13

**Authors:** The PLOS ONE Staff

Due to errors introduced during the typesetting process, Fig 1 and Fig 2 do not appear correctly; sections of the figures are distorted, and the thickness of multiple lines and borders have been increased. The publisher apologizes for the errors. Please see the correct Fig 1 and Fig 2 here.

**Fig 1:**
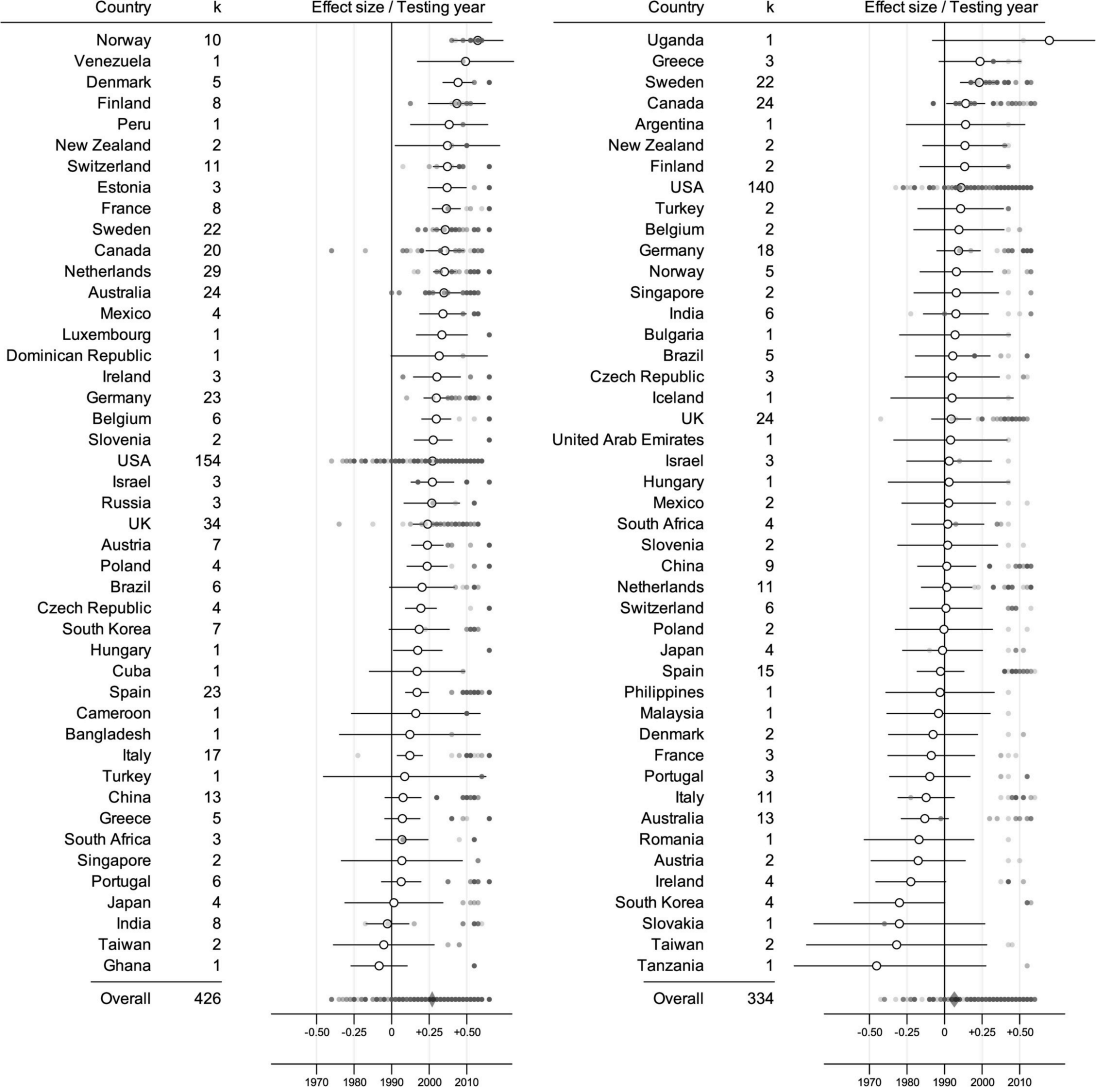
Effect size estimates for each country for both task categories. Forest plot describing the variation in sex differences in (a) Verbal and (b) Other episodic memory tasks across the (a) 45 and (b) 45 countries used in the analyses, with k = number of studies available for each country. Cohen’s *d* (unfilled circles) is presented on the x-axis, with error bars describing the 95% confidence intervals (notice that some error bars have been truncated). Filled circles indicate the year each study was carried out.

**Fig 2:**
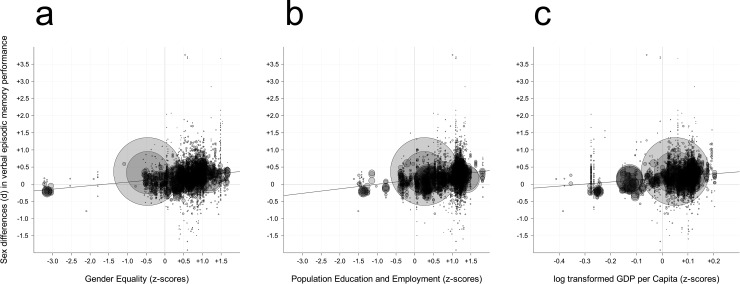
Scatterplots for the three simple effects analyses. Indicator of (a) Gender equality, (b) Population Education and Employment, (c) GDP per Capita (x-axis) plotted against sex differences in Verbal episodic memory performance (y-axis). The diameter of each data point is equal to the inverse of its squared variance. The lines indicate the best-fitting regressions.
