# Supplementary material for: The magnitude of sex differences in verbal episodic memory increases with social progress: Data from 54 countries across 40 years
Source: PLoS One. 2019 Apr 22;14(4):e0214945. doi: 10.1371/journal.pone.0214945 (PMC6476491; doi:10.1371/journal.pone.0214945)
Supplement: S1 Table — Studies included in the database with country of study, effect size d (variance) of Verbal and Other episodic memory tasks, total number of males/females participating in the study, together with country- and time-specific measures indicative of gender equality in educational and occupational opportunities, population education level and labor force participation, and GDP per capita. Effect sizes were derived by using the formulas recommended by Borenstein et al. [31] to combine intersample and intrasample data points. For more information about this procedure, see Asperholm et al. (5). Explanation of headings: Country = Country of study; d (variance) = Cohen’s d and variance of categories Verbal and Other episodic memory tasks (when combining all effect sizes within a study using verbal material or other, non-verbal, material); N Males/Females = The total number of males/females participating in the study; Gender equality = A country- and time-specific measure indicative of gender equality in educational and occupational opportunities; Population Education Employment = A country- and time-specific measure indicative of education level and labor force participation; GDP per Capita = A country- and time-specific measure of economic activity in relation to the size of the population. (PDF) [file pone.0214945.s001.pdf]

Table S1. Details of all studies used in the analyses.

Study	Country	d Verbal (variance)	d Other (variance)	N Males/ Females	Gender Equality	Population Education Employment	GDP per Capita
Abbs et al., 2011	USA	0.42 (0.09)		27/21	0.93	1.12	48374
Acevedo et al., 2010	USA	0.84 (0.09)	-0.34 (0.08)	26/24	0.92	1.17	47002
Aikins et al., 2010	USA	-0.13 (0.17)		12/12	0.92	1.17	47002
Aine et al., 2005	Australia	0.03 (0.24)		10/8	0.68	0.90	30441
Albus et al., 1997	Germany		0.37 (0.10)	20/20	-0.52	0.34	30564
Aliotti & Rajabiun, 1991	USA		0.05 (0.04)	47/66	0.84	1.14	23954
Allwood et al., 2006	Sweden		0.06 (0.05)	40/41	1.53	1.35	43085
Almela et al., 2012	Spain	0.08 (0.05)		43/42	0.56	0.66	31832
Amone et al., 2011	Italy	-0.06 (0.06)		32/32	-0.11	-0.31	35852
Anderson et al., 2004	Australia	0.11 (0.17)		12/11	0.68	0.90	23440
Anderson et al., 2006	UK	0.35 (0.05)	0.30 (0.05)	33/50	0.57	0.87	40048
Areh, 2011	Slovenia		0.26 (0.01)	119/161	0.80	0.83	23439
Arentoft et al., 2009	USA	0.99 (0.11)		19/21	0.89	1.21	48401
Arnold et al., 1987	USA	0.60 (0.03)		82/75	0.74	1.12	19115
Astur et al., 1998	USA		-0.96 (0.09)	21/27	0.66	1.29	31573
Astur et al., 2004	USA		-0.49 (0.07)	24/37	0.78	1.21	39677
Au et al., 2004	USA	0.40 (0.01)	-0.06 (0.01)	375/429	0.78	1.21	39677
Ayesa-Arriola et al., 2014	Spain	0.34 (0.03)	-0.50 (0.03)	97/62	0.63	0.68	29371
Bacon & Izaute, 2009	France		-0.21 (0.20)	12/9	0.48	0.39	45413
Badcock et al., 2011	Australia	0.42 (0.02)		159/101	1.00	1.09	51846
Bangirana et al., 2011	Uganda		0.70 (0.07)	34/27	0.28	-0.39	609
Banks et al., 1987	USA	0.83 (0.08)		23/37	0.74	1.12	19115
Barid et al., 2007	USA	0.82 (0.09)		23/28	0.85	1.21	46437
Barker-Collo et al., 2010	New Zealand	0.77 (0.40)		17/17	1.37	1.08	28201
Baron et al., 2012	USA	0.24 (0.03)	0.20 (0.03)	67/59	0.92	1.08	49782
Bearden et al., 2006	USA	0.28 (0.11)		15/25	0.85	1.19	44308
Beatty & Bruellman, 1987	USA		0.15 (0.03)	59/64	0.74	1.12	19115

Study	Country	d Verbal (variance)	d Other (variance)	N Males/ Females	Gender Equality	Population Education Employment	GDP per Capita
Becker et al., 2005	USA		-0.04 (0.09)	20/33	0.81	1.19	41922
Beinhoff et al., 2008	Germany	0.57 (0.08)		28/23	0.26	1.13	41815
Bellace et al., 2013	USA	-0.41 (0.35)	-0.56 (0.36)	5/7	0.91	1.08	51433
Bender et al., 2010	USA	0.39 (0.02)		88/183	0.92	1.17	47002
Bengner et al., 2006	Germany		0.48 (0.13)	17/15	0.19	0.96	34697
Bernardet et al., 2011	Spain		-0.74 (0.13)	29/11	0.50	0.63	30738
Beydoun et al., 2013	USA	0.17 (0.00)	-0.08 (0.00)	938/1228	0.91	1.08	51433
Bielak et al., 2012	Australia	0.41 (0.00)		3517/3635	1.01	1.10	62217
Birenbaum et al., 1994	Israel		0.42 (0.01)	204/206	0.19	0.44	12531
Blankevoort et al., 2013	Netherlands	0.62 (0.02)		98/122	0.53	1.23	49475
Bleecker et al., 1988	USA	0.55 (0.02)		87/109	0.77	1.12	20101
Block et al., 2009	USA		0.26 (0.05)	41/40	0.89	1.21	48401
Bloise & Johnson, 2007	USA	0.29 (0.06)		30/33	0.85	1.21	46437
Boeke et al., 2012	USA		0.16 (0.00)	441/449	0.92	1.08	49782
Boeuf-Cazou et al., 2011	France	0.47 (0.00)		536/483	0.59	0.46	40706
Boman, 2004	Sweden	0.10 (0.04)	0.42 (0.04)	48/48	1.40	1.19	36961
Borges & Vaughn, 1977	USA		0.84 (0.10)	22/22	0.47	1.00	8611
Börsch-Supan, 2016c	Austria	0.19 (0.00)		1820/2445	-0.29	0.67	48324
Börsch-Supan, 2016b	Austria	0.07 (0.04)		36/62	-0.49	0.47	46587
Börsch-Supan, 2016a	Austria	0.06 (0.00)		771/1057	-0.56	0.32	38242
Börsch-Supan, 2016d	Austria	-0.08 (0.02)		100/83	-0.29	0.69	50558
Börsch-Supan, 2016b	Belgium	0.29 (0.01)		147/175	0.42	0.26	44404
Börsch-Supan, 2016c	Belgium	0.17 (0.00)		1296/1554	0.50	0.24	44734
Börsch-Supan, 2016a	Belgium	0.16 (0.00)		1710/1955	0.36	0.21	36967
Börsch-Supan, 2016d	Belgium	0.13 (0.00)		684/744	0.54	0.29	46622
Börsch-Supan, 2016b	Czech Republic	0.15 (0.00)		1161/1549	0.36	0.89	18334
Börsch-Supan, 2016d	Czech Republic	0.07 (0.00)		660/870	0.47	1.09	19814
Börsch-Supan, 2016c	Czech Republic	0.06 (0.00)		1962/2574	0.44	1.00	19641
Börsch-Supan, 2016d	Denmark	0.34 (0.00)		879/987	0.89	1.15	60362
Börsch-Supan, 2016a	Denmark	0.29 (0.00)		741/852	0.68	1.21	48817
Börsch-Supan, 2016c	Denmark	0.28 (0.01)		229/213	0.86	1.18	58125

Study	Country	d Verbal (variance)	d Other (variance)	N Males/ Females	Gender Equality	Population Education Employment	GDP per Capita
Börsch-Supan, 2016b	Denmark	0.28 (0.00)		596/701	0.74	1.27	58501
Börsch-Supan, 2016c	Estonia	0.27 (0.00)		2553/3890	1.22	1.12	17491
Börsch-Supan, 2016d	Estonia	0.20 (0.01)		180/139	1.22	1.14	19155
Börsch-Supan, 2016b	France	0.35 (0.00)		417/490	0.43	0.37	41601
Börsch-Supan, 2016d	France	0.26 (0.02)		99/105	0.60	0.52	42571
Börsch-Supan, 2016a	France	0.20 (0.00)		1289/1613	0.30	0.32	34880
Börsch-Supan, 2016c	France	0.20 (0.00)		1543/1911	0.60	0.49	40838
Börsch-Supan, 2016c	Germany	0.59 (0.08)		30/32	0.35	1.32	44011
Börsch-Supan, 2016d	Germany	0.19 (0.00)		2086/2289	0.35	1.35	45601
Börsch-Supan, 2016b	Germany	0.15 (0.00)		465/514	0.26	1.13	41815
Börsch-Supan, 2016a	Germany	0.10 (0.00)		1347/1540	0.19	0.96	34697
Börsch-Supan, 2016b	Greece	-0.10 (0.00)		435/476	-0.26	0.12	28827
Börsch-Supan, 2016a	Greece	-0.04 (0.00)		1212/1391	-0.37	0.06	22552
Börsch-Supan, 2016c	Hungary	0.06 (0.00)		1284/1660			
Börsch-Supan, 2016b	Ireland	0.18 (0.00)		494/583	0.89	0.84	61314
Börsch-Supan, 2016d	Israel	0.16 (0.00)		912/1156	0.91	0.95	36281
Börsch-Supan, 2016a	Italy	0.01 (0.00)		1117/1372	-0.31	-0.38	31959
Börsch-Supan, 2016b	Italy	0.01 (0.00)		567/587	-0.24	-0.35	37699
Börsch-Supan, 2016c	Italy	-0.03 (0.00)		657/758	-0.03	-0.21	34814
Börsch-Supan, 2016d	Italy	-0.03 (0.00)		814/915	-0.02	-0.22	35368
Börsch-Supan, 2016d	Luxembourg	0.22 (0.00)		731/816	-0.03	0.50	113727
Börsch-Supan, 2016b	Netherlands	0.27 (0.00)		396/451	0.41	1.14	51241
Börsch-Supan, 2016a	Netherlands	0.25 (0.00)		1302/1488	0.34	0.98	41577
Börsch-Supan, 2016d	Netherlands	0.23 (0.00)		805/917	0.53	1.25	51425
Börsch-Supan, 2016c	Netherlands	0.19 (0.00)		397/453	0.53	1.23	49475
Börsch-Supan, 2016c	Poland	0.25 (0.02)		112/97	0.52	0.36	13142
Börsch-Supan, 2016b	Poland	0.09 (0.00)		1054/1336	0.45	0.06	11248
Börsch-Supan, 2016c	Portugal	-0.07 (0.00)		866/1093	0.75	0.04	20577
Börsch-Supan, 2016d	Slovenia	0.19 (0.00)		438/537	0.86	0.77	23144
Börsch-Supan, 2016c	Slovenia	0.10 (0.00)		1164/1483	0.85	0.77	22478
Börsch-Supan, 2016c	Spain	-0.08 (0.00)		851/954	0.60	0.68	28648

Study	Country	d Verbal (variance)	d Other (variance)	N Males/ Females	Gender Equality	Population Education Employment	GDP per Capita
Börsch-Supan, 2016b	Spain	-0.04 (0.01)		417/390	0.13	0.49	32709
Börsch-Supan, 2016a	Spain	-0.02 (0.00)		967/1330	-0.09	0.36	26511
Börsch-Supan, 2016d	Spain	-0.01 (0.00)		1543/1660	0.63	0.68	29371
Börsch-Supan, 2016c	Sweden	0.42 (0.04)		53/63	1.33	1.36	57134
Börsch-Supan, 2016d	Sweden	0.36 (0.00)		1238/1351	1.34	1.43	60283
Börsch-Supan, 2016a	Sweden	0.31 (0.00)		1379/1563	1.53	1.35	43085
Börsch-Supan, 2016b	Sweden	0.16 (0.01)		327/360	1.46	1.33	53324
Börsch-Supan, 2016b	Switzerland	0.29 (0.01)		321/411	0.14	1.47	63223
Börsch-Supan, 2016c	Switzerland	0.28 (0.00)		1207/1345	0.31	1.79	83209
Börsch-Supan, 2016d	Switzerland	0.25 (0.04)		51/54	0.36	1.80	84669
Börsch-Supan, 2016a	Switzerland	0.16 (0.00)		454/502	0.08	1.22	54798
Bowden, 1989	Australia		-0.84 (0.10)	22/22	0.23	0.78	14261
Bozikas et al., 2010	Greece	0.29 (0.07)	-0.43 (0.07)	31/31	-0.10	0.21	29711
Bracco et al., 2011	Italy	-0.06 (0.06)	-0.07 (0.06)	35/35	-0.11	-0.31	35852
Bradbard & Endsley, 1983	USA	-0.19 (0.13)		18/18	0.54	1.09	14439
Bradbard et al., 1986	USA		-0.16 (0.07)	31/25	0.72	1.11	18269
Brevik et al., 2013	Norway	0.89 (0.04)		42/83	1.21	1.21	101564
Brewster et al., 2011	Canada		0.00 (0.03)	34/44	1.11	1.33	47446
Brindal et al., 2012	Australia	0.31 (0.10)		19/21	1.01	1.10	62217
Bringmann et al., 1989	USA	-0.27 (0.06)		35/30	0.79	1.13	21483
Brito et al., 1998	Brazil		0.42 (0.01)	199/199	0.50	-0.71	5260
Brodsky et al., 2003	USA	0.36 (0.13)		15/16	0.73	1.25	38166
Brønnick et al., 2011	Norway	0.48 (0.02)		95/85	1.18	1.20	87646
Brooking et al., 2012	Brazil	0.36 (0.05)	-0.13 (0.05)	44/46	0.81	-0.12	13039
Brown et al., 2007	USA		-0.20 (0.02)	91/196	0.85	1.21	46437
Bruck, 2009	USA		0.05 (0.14)	32/26	0.89	1.21	48401
Buchanan & Tranel, 2008	USA		0.21 (0.15)	20/20	0.86	1.20	48062
Buchmann et al., 2008	Switzerland	0.12 (0.12)	0.38 (0.12)	15/21	0.14	1.47	63223
Bücker et al., 2014	Canada	0.02 (0.07)		25/39	1.12	1.34	52266
Burdick et al., 2008	USA	0.62 (0.12)	0.17 (0.11)	24/14	0.86	1.20	48062
Butts et al., 1995	USA	-0.18 (0.10)		20/20	0.69	1.21	27777

Study	Country	d Verbal (variance)	d Other (variance)	N Males/ Females	Gender Equality	Population Education Employment	GDP per Capita
Cadar et al., 2012	UK	0.31 (0.00)		490/528	0.80	1.11	41020
Cahill et al., 2001	USA		0.10 (0.19)	11/11	0.65	1.30	36450
Campeanu et al., 2013	Canada	1.84 (0.45)	-0.49 (0.35)	4/12	1.11	1.33	52495
Canli et al., 2002	USA		0.98 (0.19)	12/12	0.69	1.27	37274
Cánovas et al., 2011	Spain		-0.13 (0.21)	8/13	0.50	0.63	30738
Cansino et al., 2012	Mexico	-0.04 (0.00)	0.05 (0.00)	750/750	-0.39	-0.36	9730
Caplan & Lipman, 1995	USA		-0.50 (0.04)	53/54	0.69	1.21	27777
Carlson & Sherwin, 1998	Canada	0.03 (0.06)	-0.16 (0.06)	31/41	0.66	0.80	21770
Carnero-Pardo et al., 2012	Spain	0.14 (0.01)		156/205	0.56	0.66	31832
Carrus et al., 2010	UK		-0.13 (0.09)	21/25	0.74	1.08	37166
Casella et al., 2012	Italy	0.28 (0.23)		8/10	-0.09	-0.31	38332
Caselli et al., 2011	USA	0.72 (0.01)		187/434	0.93	1.12	48374
Cashdan et al., 2012	Tanzania		-0.48 (0.06)	15/9			740
Casiere & Ashton, 1996	USA		0.56 (0.10)	24/19	0.64	1.24	28782
Cavalieri et al., 2010	Austria		-0.30 (0.01)	269/318	-0.35	0.57	47654
Chai & Jacobs, 2009	USA		-0.16 (0.17)	26/25	0.89	1.21	48401
Chaill et al., 2001	USA		0.03 (0.18)	11/11	0.65	1.30	36450
Chapman et al., 2011	USA	0.63 (0.10)		21/21	0.93	1.12	48374
Chavez et al., 1982	USA		0.55 (0.08)	26/26	0.48	1.08	13993
Cherney, 2005	USA	0.23 (0.03)		60/60	0.81	1.19	41922
Cherney & Ryalls, 1999	USA	0.13 (0.07)	0.83 (0.11)	30/30	0.65	1.30	32949
Chin & Rickard, 2010	Australia	0.78 (0.05)		32/66	0.97	1.08	42715
Chipman & Kimura, 1998	Canada	0.40 (0.03)		73/72	0.66	0.80	21770
Choi & L'Hirondell, 2005	Canada	0.36 (0.04)	0.09 (0.04)	50/61	0.93	1.25	31980
Choi & Silverman, 2003	Canada	0.23 (0.01)	0.21 (0.01)	273/308	0.85	1.10	24168
Choudhury et al., 2003	USA		0.48 (0.02)	75/156	0.73	1.25	38166
Christensen et al., 2000	Australia	0.05 (0.01)		206/219	0.49	0.79	20536
Christensen et al., 2001	Australia	0.10 (0.01)		170/190	0.55	0.82	21665
Christensen et al., 2004	Australia	0.12 (0.00)	0.04 (0.00)	446/427	0.68	0.90	23440
Cinan et al., 2007	Turkey		0.21 (0.02)	72/74	-2.12	-1.86	7727
Clare et al., 2000	UK		0.23 (0.04)	39/71	0.82	0.62	26676

Study	Country	d Verbal (variance)	d Other (variance)	N Males/ Females	Gender Equality	Population Education Employment	GDP per Capita
Clark & Teasdale, 1985	UK	0.87 (0.07)		32/32	0.62	0.46	8179
Cockroft & Blackburn, 2008	South Africa	-0.38 (0.06)		32/32	0.40	-0.77	6161
Collaer & Evans, 1982	USA		0.19 (0.01)	134/138	0.48	1.08	13993
Colley et al., 2002	UK	0.40 (0.04)		50/50	0.82	0.66	25980
Collie et al., 1999	Australia	0.98 (0.04)		44/92	0.45	0.81	21340
Coluccia et al., 2011	Italy	-0.10 (0.01)		226/238	-0.11	-0.31	35852
Comijs et al., 2010	Netherlands	0.43 (0.00)		551/586	0.48	1.26	51900
Conde-Sala et al., 2012	Spain		0.09 (0.04)	39/73	0.56	0.66	31832
Corbeil & McKelvie, 2008	Canada	0.14 (0.08)		26/28	1.04	1.33	44545
Corrêa et al., 2012	Brazil		0.24 (0.61)	2/9	0.81	-0.12	13039
Costa et al., 2013	Portugal	0.04 (0.01)		241/262	0.75	0.04	20577
Cox & Waters, 1986	USA	0.17 (0.03)		60/60	0.72	1.11	18269
Crook et al., 1990	USA		0.40 (0.01)	149/298	0.82	1.14	22922
Crook et al., 1993	USA		-0.20 (0.01)	243/366	0.74	1.17	25493
Crotty et al., 2012	USA	0.31 (0.04)	0.11 (0.04)	58/50	0.92	1.08	49782
Cruse & Critchlow Leigh, 1987	USA	0.29 (0.02)		108/108	0.74	1.12	19115
Cserjesi et al., 2012	Netherlands	0.46 (0.03)		58/72	0.52	1.17	53537
Curry et al., 1986	USA	-0.39 (0.05)	0.15 (0.05)	48/49	0.72	1.11	18269
Daalman et al., 2011	Netherlands	0.85 (0.05)	-0.13 (0.05)	30/71	0.49	1.18	50341
Dabbs et al., 1998	USA		0.11 (0.02)	90/104	0.66	1.29	31573
Daprati et al., 2013	Italy		0.01 (0.41)	13/3	-0.03	-0.21	34814
Davey et al., 2013	USA	0.01 (0.04)		34/151	0.91	1.08	51433
Davidson, 2006	USA		0.05 (0.04)	53/53	0.85	1.19	44308
de Bartolomeis et al., 2013	Italy	0.20 (0.39)		19/3	-0.03	-0.21	34814
De Goede & Postma, 2008	Netherlands	0.32 (0.05)	0.30 (0.05)	41/40	0.41	1.14	51241
DeFries et al., 1982	USA	0.16 (0.00)		3252/3317	0.48	1.08	13993
Deloire et al., 2005	France	0.18 (0.11)	-0.12 (0.11)	14/30	0.30	0.29	33875
den Heijer et al., 2003	Netherlands	0.14 (0.01)		202/210	0.17	0.94	28817
Dennett et al., 2012	Australia		-0.92 (0.03)	60/93	1.01	1.10	62217
Dewhurst et al., 2012	UK	0.43 (0.04)		50/50	0.80	1.11	41020
Diege et al., 2010	Sweden	0.35 (0.07)		22/44	1.37	1.29	46207

Study	Country	d Verbal (variance)	d Other (variance)	N Males/ Females	Gender Equality	Population Education Employment	GDP per Capita
Dingwall et al., 2010	Australia		-0.24 (0.02)	95/111	0.97	1.08	42715
Dodrill, 1979	USA	0.32 (0.04)	-0.40 (0.04)	47/47	0.45	1.03	10587
Doty & Kerr, 2005	USA		1.40 (0.08)	30/30	0.81	1.19	41922
Drakeford et al., 2010	UK	1.00 (0.44)	0.72 (0.43)	3/13	0.74	1.08	37166
Duff et al., 2010	USA	0.15 (0.01)	-0.21 (0.01)	300/418	0.92	1.17	47002
Eals & Silverman, 1994	Canada	0.04 (0.05)	0.48 (0.03)	81/82	0.59	0.77	20017
Economou, 2009	Greece	-0.04 (0.01)		140/182	-0.21	0.14	31997
Economou et al., 2006	Greece	-0.09 (0.16)		11/16	-0.37	0.06	22552
Ecuyer-Dab & Robert, 2004	Canada		0.31 (0.02)	95/121	0.90	1.20	28172
Edelstein et al., 1998	USA	0.37 (0.01)	-0.28 (0.01)	209/300	0.66	1.29	31573
Eidelman et al., 2012	USA	0.48 (0.12)		6/10	0.92	1.08	49782
Elamin et al., 2011	Ireland	0.10 (0.03)	-0.41 (0.04)	55/61	0.94	0.71	48261
Elias et al., 1997	USA	0.10 (0.00)	-0.07 (0.00)	742/1063	0.65	1.26	30068
Ellis et al., 1973	UK		0.98 (0.07)	30/30	0.39	0.34	3030
Ellis et al., 2009	Australia	0.37 (0.01)	-0.22 (0.01)	328/440	0.91	1.08	49628
Epstein, 1974	USA	-0.57 (0.04)		57/57	0.52	0.92	6741
Ernest, 1983	Canada	0.22 (0.02)		72/137	0.33	0.75	12440
Espin et al., 2013	Spain	0.64 (0.08)		18/44	0.60	0.68	28648
Evardone & Alexander, 2009	USA		0.18 (0.05)	41/38	0.89	1.21	48401
Fein & McGillivray, 2007	USA	0.21 (0.11)	0.10 (0.11)	16/23	0.85	1.21	46437
Fein et al., 2006	USA	0.29 (0.08)	0.13 (0.08)	25/23	0.85	1.19	44308
Felmingham et al., 2012	Australia	-0.41 (0.10)		20/21	1.01	1.10	62217
Ferguson et al., 2008	USA		0.00 (0.06)	29/43	0.86	1.20	48062
Ferguson et al., 2010	USA		0.12 (0.02)	79/133	0.92	1.17	47002
Fernandez-Mendoza et al., 2009	USA		-0.14 (0.01)	312/396	0.89	1.21	48401
Ferree & Cahill, 2009	USA		-0.06 (0.10)	16/32	0.89	1.21	48401
Fichman et al., 2010	Brazil	0.26 (0.02)		65/118	0.76	-0.02	8475
File et al., 2001	UK	1.05 (0.28)		8/8	0.88	0.65	26401
Fillenbaum et al., 2011	USA	-0.14 (0.04)		72/65	0.93	1.12	48374
Findlay et al., 2009	Canada	0.16 (0.00)		9477/12744	1.05	1.35	46596
Fischer et al., 2007	Sweden		0.33 (0.19)	12/12	1.49	1.30	46256

Study	Country	d Verbal (variance)	d Other (variance)	N Males/ Females	Gender Equality	Population Education Employment	GDP per Capita
Fiske & Gatz, 2007	Sweden	0.15 (0.01)	-0.01 (0.01)	177/241	1.49	1.30	46256
Flegr et al., 2012	Czech Republic		0.24 (0.02)	100/93	0.41	0.93	21657
Foti et al., 2011	Italy		0.33 (0.32)	6/7	-0.11	-0.31	35852
Frasson et al., 2011	Italy	0.21 (0.02)		99/128	-0.11	-0.31	35852
Fried & Johanson, 2008	USA		-0.31 (0.01)	134/489	0.86	1.20	48062
Friedman et al., 2002	USA	0.67 (0.02)		108/129	0.69	1.27	37274
Frings et al., 2006	Germany		0.16 (0.20)	10/10	0.19	0.96	34697
Friswell et al., 2008	UK	-0.50 (0.24)	-0.20 (0.22)	9/9	0.63	0.98	48428
Fritsch et al., 2007	USA	0.51 (0.05)		104/107	0.85	1.21	46437
Fuentes et al., 2012	Canada	0.76 (0.22)	1.60 (0.25)	6/24	1.12	1.33	52084
Fulda et al., 2010	Germany	0.73 (0.19)		9/14	0.29	1.23	41733
Gale et al., 2008	UK	0.33 (0.02)		130/111	0.63	0.98	48428
Gale et al., 2007	USA	0.74 (0.03)	0.18 (0.03)	59/113	0.85	1.21	46437
Galea & Kimura, 1993	Canada	0.34 (0.13)	-0.54 (0.06)	49/48	0.58	0.77	20771
Gallagher & Burke, 2007	Ireland		-0.86 (0.04)	60/57	0.85	0.78	54286
Gallagher et al., 2006	UK		-0.04 (0.02)	60/60	0.57	0.87	40048
Ganguli et al., 2009	USA	0.11 (0.00)	0.00 (0.00)	748/1180	0.89	1.21	48401
Gavazzeni et al., 2012	Sweden	-0.03 (0.06)		34/38	1.30	1.34	59594
Gedney & Logan, 2004	USA		-0.11 (0.06)	37/30	0.78	1.21	39677
Geer & McGlone, 1990	USA	0.01 (0.12)		20/20	0.82	1.14	22922
Gerstorf et al., 2006	Germany	0.14 (0.01)		258/258	0.19	0.96	34697
Gibbs & Wilson, 1999	USA		-0.74 (0.09)	17/15	0.65	1.30	32949
Glaser et al., 2012	Canada	-0.02 (0.12)		17/17	1.12	1.33	52084
Göder et al., 2004	Germany		0.68 (0.26)	10/7	-0.09	0.76	30360
Gogos et al., 2010	Australia	0.23 (0.09)	-0.56 (0.10)	21/22	0.97	1.08	42715
Goldstein et al., 1998	USA	0.24 (0.15)	0.08 (0.15)	13/14	0.66	1.29	31573
Golier et al., 2005	USA	0.26 (0.22)		13/7	0.81	1.19	41922
Gonzales et al., 2012	USA	0.92 (0.08)	-0.14 (0.08)	24/30	0.92	1.08	49782
González et al., 2001	USA	0.26 (0.01)		331/470	0.65	1.30	36450
Gow et al., 2013	UK	0.26 (0.00)		544/543	0.80	1.15	41295
Grabe & Kamhawi, 2006	USA		0.28 (0.06)	36/39	0.85	1.19	44308

Study	Country	d Verbal (variance)	d Other (variance)	N Males/ Females	Gender Equality	Population Education Employment	GDP per Capita
Grabe & Samson, 2011	USA		0.14 (0.01)	193/193	0.93	1.12	48374
Grahn et al., 2013	Sweden	0.21 (0.14)		15/13	1.33	1.36	57134
Grambaite et al., 2014	Norway	0.73 (0.19)		11/12	1.23	1.20	102910
Gruzelier et al., 1999	UK	0.10 (0.04)	-0.58 (0.04)	45/50	0.76	0.57	26281
Guillem & Mograss, 2005	Canada		1.03 (0.18)	16/10	0.93	1.25	31980
Guillem et al., 2009	Canada		0.52 (0.23)	9/9	1.05	1.35	46596
Gummerum et al., 2013	UK	-0.25 (0.05)		19/19	0.80	1.15	41295
Gupta & Kaur, 1996	India	0.06 (0.05)		20/20	-2.40	-1.38	382
Gur et al., 2012	USA	0.13 (0.00)	0.06 (0.00)	1597/1851	0.92	1.08	49782
Haan et al., 2003	USA	0.55 (0.00)		585/823	0.73	1.25	38166
Haász et al., 2013	Norway	1.03 (0.05)		33/67	1.21	1.21	101564
Hagsand et al., 2013	Sweden		0.12 (0.11)	13/29	1.33	1.36	57134
Hall & Mast, 2008	Switzerland	0.69 (0.02)	0.03 (0.04)	170/172	0.14	1.47	63223
Halpern, 1985	USA	-0.09 (0.02)		39/49	0.66	1.10	17134
Halpern et al., 2011	USA	0.25 (0.08)	0.04 (0.07)	38/21	0.93	1.12	48374
Han et al., 2012	China		0.29 (0.01)	141/130	0.25	0.32	5574
Hannay & Rogers, 1979;	USA		0.06 (0.08)	24/24	0.45	1.03	10587
Harness et al., 2008	USA	0.17 (0.03)		54/54	0.86	1.20	48062
Harrington et al., 2013	USA	0.25 (0.06)	0.37 (0.07)	26/42	0.91	1.08	51433
Hassan & Rahman, 2007	UK		0.55 (0.08)	40/20	0.61	0.94	42534
Hassmén et al., 2002	Sweden	-0.40 (0.05)		28/26	1.27	1.16	26969
Haut & Barch, 2006	USA	-0.14 (0.04)	-0.20 (0.04)	47/63	0.85	1.19	44308
Hayden et al., 2005	USA	-0.12 (0.01)	-0.22 (0.01)	203/282	0.81	1.19	41922
Hazlett et al., 2010	USA	0.34 (0.06)		35/35	0.92	1.17	47002
Heisz et al., 2013	Canada		0.67 (0.05)	40/40	1.11	1.33	52495
Heller et al., 2010	USA		0.46 (0.04)	30/30	0.92	1.17	47002
Hellström et al., 2008	Sweden	0.26 (0.03)		108/51	1.46	1.33	53324
Hellvin et al., 2012	Norway	0.60 (0.04)	0.00 (0.04)	49/61	1.22	1.18	100575
Herlitz et al., 1997	Sweden	0.28 (0.00)	0.21 (0.00)	470/530	1.10	1.13	32587
Herlitz & Kabir, 2006	Bangladesh	0.10 (0.01)		194/232	-0.42	-1.50	486
Herlitz & Yonker, 2002	Sweden		0.37 (0.02)	88/99	1.27	1.16	26969

Study	Country	d Verbal (variance)	d Other (variance)	N Males/ Females	Gender Equality	Population Education Employment	GDP per Capita
Herlitz et al., 1999	Sweden	0.24 (0.02)	-0.06 (0.02)	100/100	1.09	1.11	30144
Herlitz et al., 2013	Sweden	0.39 (0.02)	0.45 (0.02)	85/102	1.33	1.36	57134
Herold et al., 2013	Germany	0.18 (0.20)		12/9	0.35	1.32	44011
Herrmann et al., 1992	UK		1.02 (0.14)	16/16	0.51	0.55	19901
Herzmann et al., 2012	USA		-0.13 (0.16)	13/13	0.92	1.08	49782
Hirata et al., 2009	Brazil	0.25 (0.00)		333/871	0.71	-0.06	8707
Hogervorst et al., 2004	UK	0.25 (0.03)	-0.11 (0.03)	79/66	0.68	0.76	32575
Holsen et al., 2008	USA		0.69 (0.39)	5/6	0.86	1.20	48062
Honda & Nihei, 2009	Japan		0.34 (0.05)	40/44	0.12	0.83	37866
Horgan et al., 2004	USA		0.30 (0.02)	82/106	0.78	1.21	39677
Horgan et al., 2009	USA	-0.06 (0.04)	0.37 (0.04)	44/64	0.89	1.21	48401
Horgan et al., 2012	USA	0.41 (0.03)		60/64	0.92	1.08	49782
Hota, 1983	India	0.15 (0.07)		32/28	-1.79	-1.64	280
Huang et al., 2008	Taiwan		-0.75 (0.09)	21/27			
Hubley, 2010	Canada		0.07 (0.08)	16/48	1.09	1.33	40773
Huestegge et al., 2012	UK		-0.17 (0.11)	18/18	0.80	1.11	41020
Hynd & Obrzut, 1978	USA	0.29 (0.10)		20/20	0.46	1.01	9471
Hyttinena et al., 2010	Finland	0.33 (0.01)		112/197	1.16	0.67	47107
Iachini et al., 2008	Italy		-0.49 (0.12)	8/9	-0.24	-0.35	37699
Iachini et al., 2005	Italy		-0.14 (0.03)	64/64	-0.32	-0.36	31175
Iachini et al., 2009	Italy		-0.40 (0.03)	70/70	-0.18	-0.30	40640
Ionescu, 2000	USA	-0.09 (0.11)		17/20	0.66	1.30	34621
Ionescu, 2002	USA	-0.01 (0.02)		95/112	0.69	1.27	37274
Ionescu, 2004	USA	-0.16 (0.09)		23/23	0.78	1.21	39677
Iqbal et al., 2009	UK	0.29 (0.17)	-0.30 (0.17)	13/11	0.68	1.04	45195
Iverson et al., 2014	Canada	0.24 (0.04)	-0.05 (0.04)	50/50	1.12	1.34	52266
James & Kimura, 1997	Canada		0.32 (0.02)	67/66	0.63	0.77	21183
Janowski et al., 2012	Poland	-0.10 (0.07)	-0.55 (0.08)	20/44	0.51	0.31	13891
Jehna et al., 2011	Austria	0.98 (0.36)		5/8	-0.33	0.61	46660
Jhoo et al., 2010	South Korea	-0.18 (0.28)		12/5	-0.55	0.30	18339
Jiang et al., 2010	USA	0.43 (0.21)	0.87 (0.22)	8/13	0.92	1.17	47002

Study	Country	d Verbal (variance)	d Other (variance)	N Males/ Females	Gender Equality	Population Education Employment	GDP per Capita
Jones et al., 1996	USA	-0.05 (0.06)		36/36	0.64	1.24	28782
Karadayi et al., 2014	Turkey	0.03 (0.15)		10/20	-1.69	-1.42	10800
Kargopoulos et al., 2003	Greece		1.04 (0.14)	16/16	-0.48	-0.21	14110
Kashyap et al., 2013	India	0.00 (0.08)	-0.16 (0.05)	81/30	-3.04	-1.47	1444
Kawano et al., 2013	Japan	0.00 (0.08)		23/27	0.19	0.89	46701
Kayser et al., 2007	USA	0.43 (0.10)		19/21	0.85	1.21	46437
Keefe et al., 2009	USA	0.61 (0.01)		170/184	0.89	1.21	48401
Keith et al., 2009	USA	-0.01 (0.00)	0.05 (0.00)	9544/10630	0.89	1.21	48401
Keith et al., 2011	USA		0.16 (0.00)	1300/1300	0.93	1.12	48374
Kelley et al., 2005	USA	-0.40 (0.36)	-0.22 (0.34)	5/7	0.81	1.19	41922
Kennet et al., 2000	USA	0.46 (0.00)		724/868	0.66	1.30	34621
Kersker et al., 2003	USA		0.19 (0.08)	17/16	0.73	1.25	38166
Kessels et al., 2006	Netherlands	0.11 (0.03)	0.09 (0.03)	61/100	0.34	0.98	41577
Kim & Hamann, 2011	South Korea	0.21 (0.13)		17/15	-0.52	0.35	22151
Kim & Kang, 1999	South Korea	0.59 (0.01)		181/176	-1.07	-0.04	8134
Kim et al., 2012	South Korea	0.18 (0.01)	-0.22 (0.01)	290/485	-0.51	0.37	24156
Kim et al., 2013	South Korea	-0.05 (0.02)	-0.38 (0.02)	101/129	-0.50	0.39	24454
Kimura & Clarke, 2002	USA	0.47 (0.05)		41/44	0.69	1.27	37274
Kimura & Seal, 2003	Canada	0.57 (0.08)		25/28	0.85	1.10	24168
Kisser et al., 2012	USA	0.01 (0.06)	0.13 (0.15)	21/94	0.92	1.08	49782
Koenig et al., 2013	USA	-0.18 (0.23)	-0.45 (0.22)	7/13	0.91	1.08	51433
Koerts et al., 2013	Netherlands	1.07 (0.07)		32/33	0.53	1.23	49475
Komulainen et al., 2008	Finland	0.35 (0.00)		684/705	1.12	0.69	48289
Kormi-Nouri et al., 2003	Sweden		0.39 (0.03)	60/60	1.34	1.17	29572
Kovac & Majerova, 1974	Slovakia		-0.31 (0.01)	123/102	-0.11	0.69	2396
Kowal et al., 2012	China	-0.07 (0.00)		5895/6751	0.25	0.33	4515
Kowal et al., 2012	Ghana	-0.22 (0.00)		2227/2039	-0.03	0.23	1323
Kowal et al., 2012	India	-0.21 (0.00)		3262/3205	-2.98	-1.38	1388
Kowal et al., 2012	Mexico	0.18 (0.00)		848/1330	-0.39	-0.36	8861
Kowal et al., 2012	Russia	0.05 (0.00)		1351/2468	0.66	0.83	10675
Kowal et al., 2012	South Africa	-0.09 (0.00)		1555/2105	0.36	-0.77	7393

Study	Country	d Verbal (variance)	d Other (variance)	N Males/ Females	Gender Equality	Population Education Employment	GDP per Capita
Kramer et al., 1997	USA	0.30 (0.01)		401/372	0.65	1.26	30068
Kramer et al., 2003	USA	0.49 (0.00)		489/530	0.73	1.25	38166
Kremen et al., 1997	USA	0.12 (0.06)	0.43 (0.06)	28/44	0.65	1.26	30068
Krohne & Hock, 2008	Germany		0.35 (0.04)	50/47	0.26	1.13	41815
Kromann et al., 2011	Denmark		-0.14 (0.03)	24/42	0.83	1.24	57648
Kroneisen & Bell, 2013	Germany		-0.02 (0.06)	19/103	0.35	1.32	44011
Kuriyama et al., 2011	Japan		-0.08 (0.17)	7/8	0.22	0.89	42935
Lachman & Agrigoroaei, 2010	USA	0.44 (0.03)		81/68	0.92	1.17	47002
Lachman et al., 2014	USA	0.43 (0.00)		1921/2326	0.90	1.06	52660
Laing et al., 2011	Australia	0.44 (0.01)		192/173	1.00	1.09	51846
Lajiness-O'Neill et al., 2011	USA	0.47 (0.07)	0.14 (0.07)	24/41	0.93	1.12	48374
Lam et al., 2008	USA	0.51 (0.00)	-0.24 (0.00)	602/849	0.86	1.20	48062
Lanca, 1998	Canada		-0.41 (0.05)	30/30	0.66	0.80	21770
Larrabee & Crook, 1993	USA	0.51 (0.00)	0.54 (0.00)	417/417	0.74	1.17	25493
Larson et al., 2003	Sweden		0.54 (0.06)	33/35	1.34	1.17	29572
Larsson et al., 2009	Sweden		0.29 (0.02)	98/104	1.41	1.33	55747
Latvala et al., 2009	Finland	0.61 (0.01)		157/248	1.09	0.73	53401
Laukka et al., 2013	Sweden	0.16 (0.00)		1011/1637	1.33	1.36	57134
Laureati et al., 2008	Netherlands		0.41 (0.03)	37/48	0.41	1.14	51241
Lauvsnes et al., 2013	Norway	-0.20 (0.16)	-0.18 (0.16)	8/33	1.21	1.21	101564
Lavoie et al., 2007	Canada	0.60 (0.19)	-0.29 (0.19)	10/12	1.00	1.27	40387
Lawrence et al., 2008	UK		0.04 (0.01)	223/215	0.63	0.98	48428
Lee et al., 2002	China	0.22 (0.02)	0.14 (0.02)	125/138	-0.03	0.60	1047
Lee et al., 2007	USA	0.41 (0.00)	-0.31 (0.00)	388/744	0.85	1.21	46437
Lee et al., 2012	Australia	0.66 (0.01)	-0.10 (0.01)	136/283	1.01	1.10	62217
Lehmann et al., 2006	UK	0.52 (0.00)		895/1087	0.57	0.87	40048
Lehrner, 1993	USA		0.69 (0.08)	27/29	0.74	1.17	25493
Lejbak et al., 2008	Canada		0.53 (0.10)	20/20	1.04	1.33	44545
Lester & Miller, 1974	USA	0.68 (0.11)		20/20	0.52	0.92	6741
Levy et al., 2005	USA		0.30 (0.08)	31/24	0.81	1.19	41922
Lewin & Herlitz, 2002	Sweden		0.36 (0.02)	93/99	1.27	1.16	26969

Study	Country	d Verbal (variance)	d Other (variance)	N Males/ Females	Gender Equality	Population Education Employment	GDP per Capita
Lewin et al., 2001	Sweden	0.15 (0.02)	-0.03 (0.02)	91/94	1.15	1.11	29283
Leynes et al., 2013	USA	-0.29 (0.16)		9/22	0.91	1.08	51433
Liben & Signorella, 1980	USA	0.00 (0.14)		16/15	0.43	1.05	11696
Liben & Signorella, 1993	USA	0.25 (0.02)		115/134	0.74	1.17	25493
Lin et al., 2008	Taiwan	0.23 (0.03)		79/72			
Lin et al., 2010	Australia	-0.16 (0.05)	0.50 (0.06)	37/38	0.97	1.08	42715
Lindholm & Christianson, 1998	Sweden		0.38 (0.02)	80/84	1.10	1.12	29898
Lindquist et al., 2009	Sweden	1.80 (0.74)	0.31 (0.54)	5/3	1.41	1.33	55747
Ling et al., 2013	USA	0.56 (0.09)		25/24	0.91	1.08	51433
Lipton et al., 2010	USA	0.18 (0.01)		154/270	0.92	1.17	47002
Lobnig et al., 2005	Germany	-0.08 (0.36)		9/4	0.06	0.81	34166
Loebach Wetherell et al., 2002	Sweden	0.25 (0.01)	0.21 (0.01)	288/416	1.27	1.16	26969
Lombardo et al., 2007	UK	0.59 (0.20)		23/7	0.61	0.94	42534
Lorenzi-Cioldi, 1993	Switzerland	0.28 (0.03)		65/80	-0.37	1.07	39436
Loskutova et al., 2009	USA	0.15 (0.05)		36/48	0.89	1.21	48401
Lövdén et al., 2007	Germany	0.00 (0.12)	-0.90 (0.14)	16/16	0.23	1.05	36448
Lovén et al., 2012	Sweden		0.12 (0.08)	24/28	1.30	1.34	59594
Lucas et al., 2005	USA	0.04 (0.02)	0.20 (0.02)	71/237	0.81	1.19	41922
Lui et al., 2011	China	0.32 (0.12)	-0.43 (0.12)	15/20	0.25	0.33	4515
Lunzer et al., 1976	UK	-0.03 (0.02)		105/105	0.33	0.39	4300
Luszcz, 1992	Australia	0.37 (0.04)		52/54	0.25	0.76	18837
Luzzi et al., 2011	Italy		-0.30 (0.01)	168/178	-0.11	-0.31	35852
Lynn & Wilson, 1993	Ireland	0.36 (0.00)		605/594	-0.08	-0.38	15732
Maass et al., 2011	Germany	0.54 (0.02)	-0.34 (0.02)	94/83	0.31	1.28	41788
Maggi et al., 2008	Italy	-0.21 (0.00)		1186/1452	-0.24	-0.35	37699
Maheu et al., 2008	USA	-0.27 (0.25)		10/7	0.86	1.20	48062
Malaspina et al., 2012	USA		-0.21 (0.15)	13/14	0.92	1.08	49782
Malek-Ahmadi et al., 2011	USA	0.73 (0.02)		82/101	0.93	1.12	48374
Malloy-Diniz et al., 2007	Brazil	0.35 (0.02)		111/112	0.67	-0.13	5808
Marchant et al., 2010	UK	0.48 (0.15)		15/12	0.74	1.08	37166
Marquié et al., 2010	France	0.37 (0.00)		1644/1554	0.54	0.44	41631

Study	Country	d Verbal (variance)	d Other (variance)	N Males/ Females	Gender Equality	Population Education Employment	GDP per Capita
Marsland et al., 2006	USA	0.31 (0.01)	0.48 (0.01)	237/223	0.85	1.19	44308
Martin et al., 2012	Australia	0.59 (0.01)	-0.37 (0.01)	241/187	1.01	1.10	62217
Martins et al., 2005	Portugal	0.13 (0.01)	-0.21 (0.01)	275/228	0.43	-0.11	18046
Martins et al., 2012	Portugal	0.29 (0.01)	-0.14 (0.01)	172/307	0.71	0.05	23195
Maruff et al., 2004	Australia	0.21 (0.14)		25/10	0.68	0.90	23440
Marx et al., 2009	USA	0.14 (0.02)	0.06 (0.02)	613/55	0.89	1.21	48401
Maseda et al., 2013	Spain	0.17 (0.19)		4/27	0.60	0.68	28648
Mast & Hall, 2006	USA		0.53 (0.01)	188/233	0.85	1.19	44308
Mataix-Cols et al., 2006	Spain	0.91 (0.11)		20/20	-0.09	0.36	26511
Mathias et al., 2013	Australia	0.51 (0.06)	0.21 (0.06)	34/37	1.02	1.09	67646
Matsuoka et al., 2012	Japan	-0.11 (0.20)		17/7	0.18	0.88	46230
May & Hutt, 1974	Canada	0.66 (0.07)		30/30	0.50	0.60	5871
McBurney et al., 1997	USA		0.82 (0.04)	57/46	0.65	1.26	30068
McCall et al., 2007	USA	0.02 (0.05)		31/46	0.85	1.21	46437
McFarlane et al., 2002	Australia		-0.03 (0.02)	51/46	0.60	0.85	19495
McGivern et al., 1997	USA	0.43 (0.01)	0.43 (0.01)	191/203	0.65	1.26	30068
McGivern et al., 1998	USA		0.73 (0.07)	30/33	0.66	1.29	31573
McGugin et al., 2012	USA		0.21 (0.02)	102/121	0.92	1.08	49782
McGuinnes et al., 1990	USA	0.55 (0.05)	0.69 (0.02)	85/94	0.82	1.14	22922
McGuinness & McLaughlin, 1982	USA		0.83 (0.06)	40/40	0.48	1.08	13993
McKelvie, 1987	Canada		0.13 (0.06)	41/64	0.43	0.77	14404
McKelvie et al., 1993	Canada		-0.71 (0.09)	15/15	0.58	0.77	20771
McMains et al., 1993	USA		0.25 (0.09)	33/16	0.74	1.17	25493
McWiliams et al., 2014	USA	0.15 (0.04)		38/68	0.90	1.06	52660
Mecklinger et al., 2011	Germany	-0.12 (0.11)		19/19	0.31	1.28	41788
Meekes et al., 2013	Netherlands	0.06 (0.10)		16/26	0.53	1.23	49475
Mehta et al., 2013	India	0.07 (0.08)	-0.04 (0.08)	42/18	-3.04	-1.47	1444
Meier, 1991	USA	0.26 (0.04)		36/93	0.84	1.14	23954
Meier et al., 2002	Switzerland	0.43 (0.01)		198/89	-0.18	1.16	38539
Meijer et al., 2011	Netherlands	0.54 (0.01)		267/320	0.49	1.18	50341
Meléndes-Moral et al., 2010	Spain		-0.04 (0.03)	19/41	0.40	0.60	32333

Study	Country	d Verbal (variance)	d Other (variance)	N Males/ Females	Gender Equality	Population Education Employment	GDP per Capita
Mellet et al., 2014	France	0.47 (0.01)		213/220	0.60	0.52	42571
Merema et al., 2013	USA	0.31 (0.03)	0.09 (0.02)	62/115	0.91	1.08	51433
Meyers-Levy & Maheswaran, 1991	USA	0.43 (0.05)		45/45	0.84	1.14	23954
Mielke et al., 2012	USA	0.51 (0.01)	0.07 (0.01)	265/218	0.92	1.08	49782
Miller & Santoni, 1986	USA		-0.67 (0.05)	23/23	0.72	1.11	18269
Minett et al., 2005	UK	-0.03 (0.07)	-0.75 (0.08)	23/37	0.63	0.80	38306
Mizuno et al., 2011	Japan	-0.10 (0.03)		55/63	0.22	0.89	42935
Moffat et al., 1998	USA		-1.59 (0.07)	40/34	0.66	1.29	31573
Mokri et al., 2013	Mexico	0.25 (0.03)		149/161	-0.32	-0.30	9721
Morales et al., 2010	Spain	-0.03 (0.01)		359/525	0.40	0.60	32333
Morgan et al., 2006	USA	0.10 (0.04)		63/45	0.85	1.19	44308
Morrens et al., 2008	Belgium	-0.08 (0.22)		20/6	0.42	0.26	44404
Mueller et al., 2008	UK		-1.34 (0.20)	12/12	0.63	0.98	48428
Müller et al., 2007	Germany	0.49 (0.16)	0.06 (0.16)	17/10	0.23	1.05	36448
Munro et al., 2012	USA	0.27 (0.00)		477/480	0.92	1.08	49782
Murre et al., 2013	Netherlands	0.12 (0.00)	0.30 (0.00)	4109/8350	0.53	1.23	49475
Nairne et al., 2009	USA	0.26 (0.03)		75/75	0.89	1.21	48401
Närhi et al., 2010	Finland	0.74 (0.10)		17/31	1.16	0.67	47107
Naveh-Benjamin et al., 2011	USA	0.38 (0.03)		61/70	0.93	1.12	48374
Newhouse et al., 2007	USA		-0.54 (0.10)	20/20	0.85	1.21	46437
Ngandu et al., 2006	Finland	0.22 (0.00)		365/627	1.10	0.60	38969
Nieto et al., 2012	Spain	0.49 (0.13)	-0.11 (0.13)	17/14	0.56	0.66	31832
Nobili et al., 2010	Italy	0.41 (0.18)		8/21	-0.15	-0.32	36977
Novotny et al., 2003	USA	0.25 (0.04)		46/52	0.73	1.25	38166
O’Hara et al., 2006	USA	0.22 (0.03)		44/79	0.85	1.19	44308
O’Sullivan, 1997	Canada	0.53 (0.14)		13/17	0.63	0.77	21183
Öberg et al., 2002	Sweden		0.36 (0.06)	36/35	1.27	1.16	26969
Kristensen & Oerbeck, 2006	Norway		-0.03 (0.07)	26/36	1.07	1.25	66775
Ojeda et al., 2010	Spain	0.66 (0.11)		34/13	0.40	0.60	32333
Østby et al., 2012	Norway		0.50 (0.04)	55/52	1.22	1.18	100575
Otero Dadin et al., 2009	Spain	0.52 (0.11)	0.22 (0.11)	20/19	0.25	0.56	35579

Study	Country	d Verbal (variance)	d Other (variance)	N Males/ Females	Gender Equality	Population Education Employment	GDP per Capita
Ott & Lyman, 1993	USA	-0.01 (0.14)	0.56 (0.14)	18/12	0.74	1.17	25493
Owen & Lynn, 1993	South Africa	0.24 (0.00)	0.13 (0.00)	1574/1637	0.06	-1.15	3557
Palmer et al., 2013	Australia		0.03 (0.01)	45/68	1.02	1.09	67646
Palomo et al., 2013	Spain	0.02 (0.02)	-0.14 (0.02)	65/114	0.60	0.68	28648
Parada et al., 2011	Spain	0.64 (0.03)		31/29	0.50	0.63	30738
Parks et al., 2011	USA	0.60 (0.02)		70/147	0.93	1.12	48374
Passamonti et al., 2011	Italy	0.17 (0.30)		10/5	-0.11	-0.31	35852
Pati & Dash, 1990	India		0.25 (0.06)	36/36	-2.01	-1.50	353
Pauli et al., 2005	Germany	0.32 (0.30)		5/11	0.06	0.81	34166
Paulo et al., 2011	Portugal	0.07 (0.01)		77/212	0.76	0.05	22540
Pauls et al., 2013	Germany		0.05 (0.01)	330/366	0.35	1.32	44011
Pavlik et al., 2013	USA	0.17 (0.03)	-0.12 (0.02)	63/135	0.91	1.08	51433
Payne et al., 2006	USA		-0.08 (0.08)	27/29	0.85	1.19	44308
Peavy et al., 2012	USA	0.44 (0.25)		5/20	0.92	1.08	49782
Pedersen et al., 2012	Denmark	0.41 (0.06)		33/39	0.84	1.22	61304
Pena-Casanova et al., 2009	Spain	0.02 (0.01)	-0.11 (0.01)	137/203	0.25	0.56	35579
Pérez-Carpinell et al., 2008	Spain		0.08 (0.04)	50/50	0.13	0.49	32709
Pérez-Carpinell et al., 2006	Spain		0.13 (0.08)	25/25	-0.09	0.36	26511
Perrig-Chiello et al., 2000	Switzerland	0.04 (0.02)		109/65	-0.27	1.13	40577
Persinger & Richards, 1995	Canada	0.89 (0.11)		20/20	0.59	0.77	19859
Phillips & Fox, 1998	UK	-0.06 (0.07)		15/15	0.70	0.57	24803
Phillips et al., 2010	UK	0.55 (0.07)	0.26 (0.07)	31/31	0.74	1.08	37166
Phillips et al., 2011	UK	0.05 (0.24)		5/28	0.80	1.11	38293
Pickel, 2009	USA		0.16 (0.02)	96/211	0.89	1.21	48401
Piper et al., 2011	USA		0.55 (0.03)	67/61	0.93	1.12	48374
Pontón et al., 1996	USA	0.04 (0.01)	-0.15 (0.01)	120/180	0.64	1.24	28782
Portin et al., 1995	Finland	0.51 (0.01)		143/179	0.58	0.22	20306
Postma et al., 1998	Netherlands		-0.86 (0.11)	20/20	-0.06	0.56	26405
Postma et al., 1999	Netherlands		-0.72 (0.08)	23/34	-0.02	0.65	27534
Postma et al., 2003	Netherlands	0.04 (0.06)	-0.17 (0.06)	32/32	0.17	0.94	28817
Potts et al., 1986	USA	-0.36 (0.03)		72/72	0.72	1.11	18269

Study	Country	d Verbal (variance)	d Other (variance)	N Males/ Females	Gender Equality	Population Education Employment	GDP per Capita
Pouliot & Gagnon, 2005	Canada		0.07 (0.03)	60/60	0.93	1.25	31980
Rabitt et al., 1995	UK	0.24 (0.00)		583/1426	0.56	0.50	19709
Ragland et al., 2000	USA	0.70 (0.14)	0.90 (0.15)	16/14	0.66	1.30	34621
Rahman & Clarke, 2005	UK	0.89 (0.11)		19/20	0.63	0.80	38306
Rahman et al., 2003	UK	0.12 (0.03)	0.22 (0.04)	60/60	0.77	0.72	28301
Rahman et al., 2005	UK	0.74 (0.08)	0.48 (0.08)	26/26	0.63	0.80	38306
Rahman, Bakare, & Serinsu, 2011	UK		0.06 (0.07)	30/30	0.80	1.11	38293
Rahman, Newland, & Smyth, 2011	UK		0.18 (0.04)	70/35	0.80	1.11	38293
Rao & Moely, 1989	India	-0.78 (0.07)		29/29	-1.98	-1.52	361
Rapeli et al., 2007	Finland	0.31 (0.24)	-0.13 (0.25)	8/9	1.10	0.65	41121
Rapisarda et al., 2013	Singapore	0.07 (0.02)	0.27 (0.02)	87/84	0.19	0.69	54451
Raz et al., 2009	USA		0.25 (0.02)	64/125	0.89	1.21	48401
Razumnikova & Vol'f, 2007	Russia	1.23 (0.15)		16/16	0.69	0.72	6920
Read et al., 2006	Sweden	0.25 (0.00)		561/799	1.53	1.35	43085
Rehmman & Herlitz, 2006	Sweden	-0.03 (0.02)	0.38 (0.02)	88/109	1.53	1.35	43085
Rehnman & Herlitz, 2007	Sweden	0.24 (0.02)	0.44 (0.02)	107/112	1.49	1.30	46256
Reijmer et al., 2013	Netherlands	0.26 (0.12)		21/14	0.53	1.23	49475
Reis et al., 2013	USA	0.54 (0.00)		1132/1388	0.91	1.08	51433
Reiswich et al., 2012	Germany		-0.40 (0.09)	20/27	0.34	1.33	45936
Resmini et al., 2012	Spain	0.50 (0.15)	0.20 (0.15)	9/25	0.56	0.66	31832
Ridout et al., 2009	UK		-0.81 (0.34)	4/14	0.68	1.04	45195
Rizk-Jackson et al., 2006	USA		-0.23 (0.16)	14/13	0.85	1.19	44308
Robinson et al., 1996	USA	0.15 (0.03)		78/61	0.64	1.24	28782
Romano et al., 2014	Italy	0.32 (0.11)	-0.05 (0.11)	18/19	-0.02	-0.22	35368
Rosenbloom et al., 2005	USA		0.28 (0.08)	21/29	0.81	1.19	41922
Rothen & Meier, 2009	Switzerland		-0.04 (0.31)	6/7	0.25	1.61	72120
Rucklidge, 2006	New Zealand	0.28 (0.06)	0.04 (0.06)	30/35	0.96	1.12	27751
Ruff et al., 1988	USA	0.45 (0.03)		68/72	0.77	1.12	20101
Ruffieux et al., 2010	Cameroon	0.22 (0.04)		53/67	0.01	0.03	1165
Ruggiero et al., 2008	Italy		-0.62 (0.03)	46/46	-0.24	-0.35	37699
Ruiz de Azua et al., 2013	Spain	0.29 (0.11)		23/15	0.60	0.68	28648

Study	Country	d Verbal (variance)	d Other (variance)	N Males/ Females	Gender Equality	Population Education Employment	GDP per Capita
Rupp et al., 2006	Germany	0.60 (0.14)		16/14	0.19	0.96	34697
Sabia et al., 2009	UK	0.05 (0.00)		3937/1613	0.68	1.04	45195
Said et al., 1990	Australia	0.26 (0.08)		25/28	0.24	0.78	17811
Salthouse & Siedlecki, 2007	USA	0.26 (0.01)	0.09 (0.01)	102/225	0.85	1.21	46437
Santos et al., 2005	Brazil		0.00 (0.03)	64/63	0.62	-0.24	3596
Santos et al., 2013	Portugal	-0.02 (0.01)		226/261	0.75	0.04	20577
Savage & Gouvier, 1992	USA	0.06 (0.03)		66/68	0.79	1.13	24405
Savaskan et al., 2007	Switzerland		-0.51 (0.23)	9/9	0.11	1.35	57349
Scanlon Jones, 1984	USA		0.30 (0.01)	234/225	0.60	1.10	15561
Schaefer et al., 2013	Switzerland		-0.36 (0.11)	17/20	0.31	1.79	83209
Schatz et al., 2012	USA	0.15 (0.00)	-0.01 (0.00)	1426/726	0.92	1.08	49782
Schirmer et al., 2013	Singapore	0.05 (0.04)		48/48	0.19	0.69	54451
Schmitzer-Torbert, 2007	USA		-0.03 (0.04)	22/23	0.85	1.21	46437
Schofield et al., 2012	Australia	0.54 (0.17)		9/20	1.01	1.10	62217
Schretlen et al., 2007	USA	0.32 (0.04)	0.20 (0.04)	49/48	0.85	1.21	46437
Schwartz & Philippe, 1991	USA		0.72 (0.02)	102/102	0.84	1.14	23954
Seasmon et al., 2002	USA	-0.02 (0.04)		50/50	0.69	1.27	37274
Segalàs et al., 2010	Spain	0.31 (0.09)	-0.55 (0.09)	31/19	0.40	0.60	32333
Segura et al., 2009	Spain	0.38 (0.12)	0.72 (0.12)	18/17	0.25	0.56	35579
Serra et al., 2010	Italy	0.18 (0.18)		12/11	-0.15	-0.32	36977
Sharps et al., 1993	USA		0.01 (0.14)	8/8	0.74	1.17	25493
Shi et al., 2012	China	0.14 (0.02)		90/156	0.25	0.32	5574
Shichita et al., 1986	Japan		-0.40 (0.01)	145/157	-0.39	0.19	11466
Silverman et al., 2007	Argentina		0.41 (0.02)	164/103	0.19	0.16	6640
Silverman et al., 2007	Australia		0.29 (0.00)	4717/4435	0.80	1.01	36085
Silverman et al., 2007	Austria		0.14 (0.01)	203/173	-0.50	0.37	40431
Silverman et al., 2007	Belgium		0.46 (0.00)	871/615	0.39	0.21	38852
Silverman et al., 2007	Brazil		0.21 (0.01)	197/138	0.67	-0.13	5808
Silverman et al., 2007	Bulgaria		0.34 (0.01)	177/187	0.95	0.09	4456
Silverman et al., 2007	Canada		0.34 (0.00)	6750/6502	1.00	1.27	40387
Silverman et al., 2007	China		0.18 (0.01)	215/188	0.13	0.40	2082

Study	Country	d Verbal (variance)	d Other (variance)	N Males/ Females	Gender Equality	Population Education Employment	GDP per Capita
Silverman et al., 2007	Czech Republic		0.38 (0.02)	167/113	0.37	0.91	15159
Silverman et al., 2007	Denmark		0.21 (0.00)	447/421	0.72	1.28	52041
Silverman et al., 2007	Finland		0.44 (0.00)	1016/814	1.10	0.65	41121
Silverman et al., 2007	France		0.20 (0.00)	623/446	0.35	0.34	36545
Silverman et al., 2007	Germany		0.39 (0.00)	985/650	0.23	1.05	36448
Silverman et al., 2007	Greece		0.40 (0.00)	456/432	-0.31	0.11	24801
Silverman et al., 2007	Hungary		0.30 (0.02)	108/115			
Silverman et al., 2007	Iceland		0.32 (0.02)	105/110	1.26	1.50	56097
Silverman et al., 2007	India		0.22 (0.00)	2670/699	-2.80	-1.16	817
Silverman et al., 2007	Ireland		0.26 (0.00)	2868/2668	0.85	0.78	54286
Silverman et al., 2007	Israel		0.09 (0.01)	244/163	0.90	0.75	21905
Silverman et al., 2007	Italy		0.32 (0.01)	279/199	-0.27	-0.35	33411
Silverman et al., 2007	Japan		0.28 (0.01)	314/189	0.10	0.75	34076
Silverman et al., 2007	Malaysia		0.23 (0.01)	330/300	-0.57	-0.30	6195
Silverman et al., 2007	Mexico		0.29 (0.01)	241/154	-0.68	-0.42	8666
Silverman et al., 2007	Netherlands		0.37 (0.00)	1378/1030	0.36	1.03	44454
Silverman et al., 2007	New Zealand		0.43 (0.00)	1089/1070	1.05	1.15	26671
Silverman et al., 2007	Norway		0.36 (0.01)	392/287	1.09	1.25	74115
Silverman et al., 2007	Philippines		0.24 (0.01)	194/233	0.35	-0.50	1395
Silverman et al., 2007	Poland		0.37 (0.01)	236/254	0.45	0.05	9000
Silverman et al., 2007	Portugal		0.23 (0.01)	223/167	0.54	-0.02	19821
Silverman et al., 2007	Romania		0.10 (0.01)	192/195	0.14	-0.05	5829
Silverman et al., 2007	Singapore		0.30 (0.00)	1110/1233	-0.44	0.18	33580
Silverman et al., 2007	Slovenia		0.20 (0.02)	148/113	0.67	0.71	19726
Silverman et al., 2007	South Africa		0.23 (0.01)	256/156	0.42	-0.85	5668
Silverman et al., 2007	Spain		0.30 (0.00)	543/337	0.03	0.43	28483
Silverman et al., 2007	Sweden		0.33 (0.00)	939/451	1.49	1.30	46256
Silverman et al., 2007	Switzerland		0.34 (0.01)	366/228	0.11	1.35	57349
Silverman et al., 2007	Turkey		0.35 (0.00)	709/659	-2.12	-1.86	7727
Silverman et al., 2007	UK		0.28 (0.00)	46763/45928	0.61	0.94	42534

Study	Country	d Verbal (variance)	d Other (variance)	N Males/ Females	Gender Equality	Population Education Employment	GDP per Capita
Silverman et al., 2007	United Arab Emirates		0.31 (0.01)	194/133	0.33	0.74	42950
Silverman et al., 2007	USA		0.34 (0.00)	51084/45265	0.85	1.21	46437
Simons et al., 2004	USA	-0.33 (0.08)	0.68 (0.09)	20/39	0.78	1.21	39677
Simpson et al., 2005	Ireland		-0.25 (0.01)	196/191	0.74	0.52	47641
Skjerve et al., 2007	Norway	-0.05 (0.06)		27/39	1.09	1.25	74115
Slegers et al., 2011	Netherlands	0.36 (0.00)		613/641	0.49	1.18	50341
Slone et al., 2000	USA		0.24 (0.04)	35/94	0.66	1.30	34621
Smeets et al., 2006	Netherlands	-0.28 (0.14)		14/15	0.34	0.98	41577
Smith et al., 2011	Canada	0.67 (0.12)		14/26	1.11	1.33	47446
Smith & Fein, 2010	USA		0.40 (0.07)	33/25	0.92	1.17	47002
Smits et al., 1997	Netherlands	0.11 (0.04)		52/48	-0.10	0.44	28699
Snitz et al., 2009	USA	0.52 (0.00)	-0.38 (0.00)	1650/1418	0.89	1.21	48401
Snitz et al., 2010	USA		0.21 (0.00)	743/1178	0.92	1.17	47002
Soares et al., 2012	Brazil	0.15 (0.01)		107/195	0.81	-0.12	13039
Sobal & Juhasz, 1977	USA	1.28 (0.05)		48/48	0.47	1.00	8611
Söderlund et al., 2006	Germany	0.08 (0.03)		75/83	0.19	0.96	34697
Söderlund et al., 2006	Italy	0.40 (0.04)		52/48	-0.31	-0.38	31959
Söderlund et al., 2006	Netherlands	0.22 (0.01)		118/109	0.34	0.98	41577
Söderlund et al., 2006	Poland	0.14 (0.04)		62/50	0.48	0.09	7976
Söderlund et al., 2006	Spain	-0.03 (0.04)		51/42	-0.09	0.36	26511
Söderlund et al., 2006	Sweden	0.22 (0.03)		64/69	1.53	1.35	43085
Söderlund et al., 2006	UK	-0.08 (0.03)		134/43	0.57	0.87	40048
Solowij et al., 2011	Australia	0.06 (0.08)		18/44	1.00	1.09	51846
Sommer et al., 2013	Germany		0.35 (0.01)	393/415	0.35	1.32	44011
Song et al., 2012	China	0.16 (0.08)		26/26	0.25	0.32	5574
Sonnega et al., 2014	USA	0.29 (0.00)		9461/9060	0.70	1.18	26465
Sosa et al., 2009	China	-0.16 (0.00)		893/1132	0.19	0.36	3441
Sosa et al., 2009	Cuba	0.04 (0.00)		933/1688	-0.11	-0.25	5386
Sosa et al., 2009	Dominican Republic	0.19 (0.00)		612/1157	0.57	-0.33	4997
Sosa et al., 2009	India	-0.17 (0.00)		722/1099	-2.89	-1.27	1023

Study	Country	d Verbal (variance)	d Other (variance)	N Males/ Females	Gender Equality	Population Education Employment	GDP per Capita
Sosa et al., 2009	Mexico	0.30 (0.00)		679/1144	-0.51	-0.39	9579
Sosa et al., 2009	Peru	0.25 (0.00)		694/1073	-0.08	0.84	4245
Sosa et al., 2009	Venezuela	0.37 (0.00)		666/1160	0.49	-0.22	11225
Spiers et al., 2008	USA		0.79 (0.11)	20/20	0.86	1.20	48062
Squeglia et al., 2011	USA		0.19 (0.07)	31/24	0.93	1.12	48374
Stangor, 1988	USA		0.81 (0.08)	19/39	0.77	1.12	20101
Staresina et al., 2005	Austria	0.02 (0.21)		9/10	-0.58	0.19	36693
Stein et al., 2012	Germany	0.36 (0.00)		474/976	0.34	1.33	45936
Steptoe et al., 2013	UK	0.15 (0.00)		5024/6043	0.68	0.76	32575
Stewart et al., 2001	UK	0.18 (0.01)		123/162	0.88	0.65	26401
Stijntjes et al., 2013	Netherlands	0.62 (0.01)		247/253	0.53	1.23	49475
Stumpf, 1998	USA		0.16 (0.00)	1274/864	0.66	1.29	31573
Stumpf & Jackson, 1994	Germany	0.36 (0.00)	0.07 (0.00)	96968/90142	-0.47	0.25	25489
Su et al., 2007	Taiwan		0.17 (0.11)	16/21			
Sunderaraman et al., 2013	USA	0.98 (0.04)		26/36	0.91	1.08	51433
Sung & Dawis, 1981	USA		0.04 (0.00)	464/545	0.42	1.07	12598
Susilo et al., 2013	USA	0.12 (0.00)	0.15 (0.00)	936/1095	0.91	1.08	51433
Swan et al., 2005;	USA	0.83 (0.02)		196/60	0.81	1.19	41922
Takei et al., 2009	Japan	0.36 (0.19)		23/7	0.12	0.83	37866
Tamm et al., 2013	Australia	0.72 (0.23)		14/7	1.02	1.09	67646
Temple & Cornish, 1993	UK	0.59 (0.03)	0.04 (0.03)	64/64	0.53	0.52	20487
Terry et al., 2013	USA	0.38 (0.16)	-0.07 (0.15)	10/19	0.91	1.08	51433
Thakur et al., 1981	India		1.11 (0.06)	40/40	-1.72	-1.68	272
Thomson et al., 2005	UK	-0.10 (0.01)		222/310	0.63	0.80	38306
Timothy, 2014	USA	0.28 (0.00)		1664/3124	0.90	1.06	52660
Tippet et al., 2009	Canada		-0.74 (0.18)	12/12	1.05	1.35	46596
Toomela, 2012	Estonia	0.30 (0.03)		80/55	1.28	1.11	17454
Torniainen et al., 2011	Finland	0.48 (0.03)		61/62	1.12	0.66	46205
Torrent et al., 2011	Spain	0.75 (0.13)		13/22	0.50	0.63	30738
Torres et al., 2013	Brazil	-0.45 (0.14)		14/15	0.81	-0.14	12157
Tottenham et al., 2003	Canada		0.73 (0.07)	31/31	0.85	1.10	24168

Study	Country	d Verbal (variance)	d Other (variance)	N Males/ Females	Gender Equality	Population Education Employment	GDP per Capita
Townes et al., 2008	USA	0.01 (0.01)		276/227	0.86	1.20	48062
Trachtenberg et al., 2005	Taiwan	-0.26 (0.00)		1202/1134			
Trahan & Quintana, 1990	USA	0.21 (0.03)	-0.18 (0.03)	70/70	0.82	1.14	22922
Tropp Sneider et al., 2011	USA		-0.78 (0.27)	8/8	0.93	1.12	48374
Unsworth, 2010	USA	0.09 (0.03)	0.15 (0.03)	64/101	0.92	1.17	47002
Unterhalter et al., 2007	UK	0.21 (0.11)		20/20	0.61	0.94	42534
Unwerzagt et al., 2011	USA	0.30 (0.02)		74/107	0.93	1.12	48374
Uttl et al., 2002	USA	0.13 (0.01)		174/177	0.69	1.27	37274
Uttner et al., 2011	Germany	0.47 (0.28)	0.07 (0.30)	8/7	0.31	1.28	41788
Vakil & Blachstein, 1994	Israel		0.29 (0.02)	103/87	0.19	0.44	12531
Vakil & Blachstein, 1997	Israel	0.15 (0.01)		257/271	0.43	0.53	19286
Vakil et al., 2010	Israel	0.31 (0.00)		700/679	0.94	0.87	27796
Valis et al., 2011	Czech Republic	1.35 (0.45)	-0.74 (0.42)	7/5	0.38	0.91	19764
van Boxtel et al., 1996	Netherlands	0.56 (0.05)		40/40	-0.16	0.38	28885
van der Werf et al., 2012	Netherlands	0.47 (0.01)		275/347	0.52	1.17	53537
van Exel et al., 2002;	Netherlands	0.35 (0.01)		156/288	0.14	0.89	26584
van Hooren et al., 2007	Netherlands	0.50 (0.01)		292/286	0.36	1.03	44454
van Oostrom et al., 2012	Netherlands	0.22 (0.01)		181/323	0.52	1.17	53537
Vanhoutte et al., 2012	Belgium	0.60 (0.21)		10/10	0.49	0.23	47700
Vaskinn et al., 2011	Norway	0.40 (0.01)		182/158	1.18	1.20	87646
Veena et al., 2010	India		0.01 (0.01)	261/281	-2.94	-1.33	1125
Veena et al., 2014	India	0.01 (0.01)		261/279	-3.04	-1.47	1456
Venter & Louw, 2004	South Africa		-0.04 (0.01)	191/192	0.35	-1.05	3807
Venter & Low, 2005	South Africa		0.13 (0.01)	112/96	0.29	-0.99	4901
Verbaam et al., 2007	Netherlands	0.99 (0.06)		42/33	0.36	1.03	44454
Vilberg & Rugg, 2012	USA	-0.25 (0.23)	-0.39 (0.23)	8/10	0.92	1.08	49782
Villardita et al., 1981	Italy	-0.06 (0.03)	0.15 (0.03)	68/54	-0.74	-1.03	8429
Vitulli & Henderson, 1994	USA		-0.13 (0.05)	33/68	0.70	1.18	26465
Volf & Razumnikova, 2001	Russia	1.46 (0.25)		10/10	0.47	0.60	1772
Volz-Sidiropoulou & Gauggel, 2011	Germany	0.54 (0.05)		40/40	0.31	1.28	41788
Wagner et al., 2012	Germany	0.42 (0.00)		497/758	0.34	1.33	45936

Study	Country	d Verbal (variance)	d Other (variance)	N Males/ Females	Gender Equality	Population Education Employment	GDP per Capita
Wagovich et al., 2012	USA	0.30 (0.27)		6/10	0.92	1.08	49782
Wahlin et al., 2006	USA	0.23 (0.01)		146/240	0.85	1.19	44308
Waldstein & I., 2004	USA	0.15 (0.07)	0.29 (0.07)	30/26	0.78	1.21	39677
Walhovd et al., 2006	Norway	0.56 (0.06)		31/40	1.07	1.25	66775
Wang, 2012b	China	0.84 (0.09)		23/28	0.25	0.32	5574
Wang, 2012a	China		0.01 (0.05)	37/37	0.25	0.32	5574
Wang, 2013	China		0.15 (0.04)	45/48	0.24	0.31	6265
Wang & Fu, 2009	China	0.32 (0.10)	0.23 (0.11)	20/20	0.19	0.36	3441
Wang & Fu, 2010	China	0.09 (0.03)	0.13 (0.03)	75/73	0.22	0.35	3800
Wang et al., 2013	China	-0.21 (0.00)		744/719	0.24	0.31	6265
Weintraub et al., 2009	USA	0.25 (0.00)		3154/6345	0.89	1.21	48401
Weirich et al., 2011	Germany		0.47 (0.12)	17/19	0.31	1.28	41788
Weiss et al., 2006	Austria	0.11 (0.05)		40/40	-0.56	0.32	38242
Wesson Ashford et al., 2014	USA	0.00 (0.01)		272/596	0.90	1.06	52660
West et al., 1992	USA	0.32 (0.00)	0.23 (0.00)	1000/1343	0.79	1.13	24405
West et al., 2002	USA		0.61 (0.02)	37/43	0.69	1.27	37274
Wicks et al., 2009	UK	-0.07 (0.12)		22/13	0.68	1.04	45195
Widmann et al., 2012	Germany	0.63 (0.14)	-0.06 (0.13)	14/17	0.34	1.33	45936
Wiebe & Watkins, 1980	USA	0.13 (0.02)	0.12 (0.02)	100/100	0.43	1.05	11696
Wiederholt et al., 1993	USA	0.61 (0.01)	-0.19 (0.01)	530/610	0.74	1.17	25493
Wilhelm & Van Klink, 2007	Netherlands		0.02 (0.02)	76/96	0.36	1.03	44454
Wingbermühle et al., 2012	Netherlands	0.61 (0.10)	-0.27 (0.10)	17/25	0.52	1.17	53537
Woicik et al., 2009	USA	0.07 (0.11)		45/11	0.89	1.21	48401
Wolf et al., 2001	Germany	0.72 (0.14)		25/11	-0.60	0.52	23719
Wolff et al., 2013	Germany		0.10 (0.07)	28/28	0.35	1.32	44011
Woo et al., 2012	South Korea	0.07 (0.02)	-0.11 (0.02)	111/79	-0.51	0.37	24156
Woolley et al., 2010	Belgium		-0.54 (0.19)	11/11	0.47	0.25	44881
Xu et al., 2011	China	0.15 (0.00)		6798/18168	0.25	0.33	4515
Yang et al., 2012	China	-0.32 (0.00)		837/989	0.25	0.32	5574
Yang Zhang et al., 2012	USA		-0.02 (0.01)	589/215	0.92	1.08	49782
Ye et al., 2012	South Korea	0.29 (0.01)	-0.46 (0.01)	245/685	-0.51	0.37	24156

Study	Country	d Verbal (variance)	d Other (variance)	N Males/ Females	Gender Equality	Population Education Employment	GDP per Capita
Young, 1979	USA	-0.09 (0.14)	-0.90 (0.14)	16/16	0.45	1.03	10587
Young & Wilson, 1994	USA	0.06 (0.08)		24/28	0.70	1.18	26465
Youngjohn et al., 1991	USA	0.31 (0.00)		689/845	0.84	1.14	23954
Ystad et al., 2009	Norway	0.81 (0.03)		50/120	1.16	1.36	96881
Yurgelun-Todd et al., 2003	USA		-0.08 (0.12)	13/24	0.73	1.25	38166
Zahodne et al., 2014	USA	0.16 (0.00)		328/726	0.90	1.06	52660
Zanello et al., 2006	Switzerland	-0.14 (0.20)		9/11	0.08	1.22	54798
Zehnder et al., 2009	Switzerland	0.60 (0.02)		136/88	0.25	1.61	72120
Wang et al., 2010	China	0.24 (0.20)		10/10	0.22	0.35	3800
Zhang et al., 2012	China		-1.18 (0.33)	21/4	0.25	0.32	5574
Zhong et al., 2014	USA	0.61 (0.00)		593/786	0.90	1.06	52660
Zimmerman et al., 2012	USA	0.29 (0.01)		208/341	0.92	1.08	49782
Zoladz et al., 2013	USA	0.17 (0.09)		18/30	0.91	1.08	51433

Studies included in the database with country of study, effect size *d* (variance) of *Verbal* and *Other* episodic memory tasks, total number of males/females participating in the study, together with country- and time-specific measures indicative of gender equality in educational and occupational opportunities, population education level and labor force participation, and GDP per capita. Explanation of headings: Country = Country of study; *d* (variance) = Cohen's *d* and variance of categories *Verbal* and *Other* episodic memory tasks (when combining all effect sizes within a study using verbal material or other, non-verbal, material); N Males/Females = The total number of males/females participating in the study; Gender equality = A country- and time-specific measure indicative of gender equality in educational and occupational opportunities; Population Education Employment = A country- and time-specific measure indicative of education level and labor force participation; GDP per Capita = A country- and time-specific measure of economic activity in relation to the size of the population.
