# Supplementary material for: The magnitude of sex differences in verbal episodic memory increases with social progress: Data from 54 countries across 40 years
Source: PLoS One. 2019 Apr 22;14(4):e0214945. doi: 10.1371/journal.pone.0214945 (PMC6476491; doi:10.1371/journal.pone.0214945)
Supplement: S2 Table — Summary table of regression analyses for Verbal excluding data from databases used by Weber et al. [10] and Bonsang et al. [25], and for Verbal only containing data used by Weber et al. [10] and Bonsang et al. [25]. (*) p < .10; * p < .05; ** p < .01; *** p < .001. (PDF) [file pone.0214945.s002.pdf]

Table S2. **Summary table of additional sensitivity regression analyses.**

			Simple model 1	Simple model 2	Simple model 3	Combined model
Verbal, excluding databases						
	Intercept					
		Intercept estimate	0.21***	0.18***	-0.47(*)	0.13
		Intercept standard error	0.02	0.03	0.28	0.39
	Gender Equality					
		B estimate	0.11***			0.04
		B standard error	0.03			0.04
	Population Education and Work					
		B estimate		0.15***		0.11*
		B standard error		0.03		0.05
	GDP per capita					
		B estimate			0.07**	0.01
		B standard error			0.03	0.04
	Observations		220	220	220	220
	Multiple R^2		0.07	0.09	0.05	0.10
	Adjusted R^2		0.07	0.09	0.05	0.08
Verbal, only databases						
	Intercept					
		Intercept estimate	0.10***	0.05*	-1.00***	-0.54**
		Intercept standard error	0.02	0.02	0.11	0.20
	Gender Equality					
		B estimate	0.12***			0.04
		B standard error	0.02			0.03
	Population Education and Work					
		B estimate		0.15***		0.08**
		B standard error		0.02		0.03
	GDP per capita					
		B estimate			0.11***	0.06**
		B standard error			0.01	0.02
	Observations		64	64	64	64
	Multiple R^2		0.28	0.43	0.35	0.52
	Adjusted R^2		0.27	0.42	0.34	0.49

Summary table of regression analyses for *Verbal* excluding data from databases used by Weber et al., (2014) and Bonsang et al. (2017), and for *Verbal* only containing data used by Weber et al., (2014) and Bonsang et al. (2017). (*) p <.10; * $p < .05$; ** $p < .01$; *** $p < .001$.
