# Supplementary material for: The magnitude of sex differences in verbal episodic memory increases with social progress: Data from 54 countries across 40 years
Source: PLoS One. 2019 Apr 22;14(4):e0214945. doi: 10.1371/journal.pone.0214945 (PMC6476491; doi:10.1371/journal.pone.0214945)
Supplement: S1 References — (PDF) [file pone.0214945.s003.pdf]

Table S2. References of studies included in the analyses.

Abbs, B., Liang, L., Makris, N., Tsuang, M., Seidman, L. J., & Goldstein, J. M. (2011). Covariance modeling of MRI brain volumes in memory circuitry in schizophrenia: Sex differences are critical. *Neuroimage*, *56(4)*, 1865–1874. doi:10.1016/j.neuroimage.2011.03.079

Acevedo, S. F., Piper, B. J., Craytor, T. S. B., & Raber, J. (2010). Apolipoprotein E4 and sex affect neurobehavioral performance in primary school children. *Pediatric Research*, *67(3)*, 293–299. doi:10.1203/PDR.0b013e3181cb8e68

Aikins, D. E., Anticevic, A., Kiehl, K. A., & Krystal, J. H. (2010). Sex-related differences in amygdala activity influences immediate memory. *NeuroReport*, *21*, 273–276. doi:10.1097/WNR.0b013e328335b3f9

Aine, C. J., Adair, J. C., Knoefel, J. E., Hudson, D., Qualls, C., Kovacevic, S., . . . Stephen, J. M. (2005). Temporal dynamics of age-related differences in auditory incidental verbal learning. *Cognitive Brain Research*, *24*, 1–18. doi:10.1016/j.cogbrainres.2004.10.024

Albus, M., Hubmann, W., Mohr, F., Scherer, J., Sobizack, N., Franz, U., . . . Wahlheim, C. (1997). Are there gender differences in neuropsychological performance in patients with first-episode schizophrenia? *Schizophrenia Research*, *28(1)*, 39–50.

Aliotti, N. C. & Rajabiun, D. A. (1991). Visual memory development in preschool children. *Perceptual and Motor Skills*, *73(3, Pt 1)*, 792–794.

Allwood, C. M., Granhag, P. A., & Jonsson, A.-C. (2006). Child witnesses' metamemory realism. *Scandinavian Journal of Psychology*, *47*, 461–470. doi:10.1111/j.1467-9459.2006.00530.x

Almela, M., van der Meij, L., Hidalgo, V., Villada, C., & Salvador, A. (2012). The cortisol awakening response and memory performance in older people. *Psychoneuroendocrinology*, *37(12)*, 1929–1940. doi:10.1016/j.psychoneuen.2012.04.009

Amone, B., Pompili, A., Tavares, M. C., & Gasbarri, A. (2011). Sex-related memory recall and talkativeness for emotional stimuli. *Frontiers in Behavioral Neuroscience*, *5*, 1–9. doi:10.3389/fnbeh.2011.00052

Anderson, E. J., de Jager, C. A., & Iversen, S. D. (2006). The Placing Test: Preliminary investigations of a quick and simple memory test designed to be sensitive to pre-dementia Alzheimer's disease but not to normal ageing. *Journal of Clinical and Experimental Neuropsychology*, *28*, 843–858. doi:10.1080/13803390591001016

Anderson, V. A., Morse, S. A., Catroppa, C., Haritou, F., & Rosenfeld, J. V. (2004). Thirty month outcome from early childhood head injury: A prospective analysis of neurobehavioural recovery. *Brain*, *127*, 2608–2620. doi:10.1093/brain/awh320

Areh, I. (2011). Gender-related differences in eyewitness testimony. *Personality and Individual Differences*, *50(5)*, 559–563. doi:10.1016/j.paid.2010.11.027

Arentoft, A., Sweat, V., Starn, V., Oliver, S., Hassenstab, J., Bruehl, H., . . . Convit, A. (2009). Plasma BDNF is reduced among middle-aged and elderly women with impaired insulin function: Evidence of a compensatory mechanism. *Brain and Cognition*, *71*, 147–152. doi:10.1016/j.bandc.2009.04.009

Arnold, M. E., Petros, T. V., Beckwith, B. E., Coons, G., & Gorman, N. (1987). The effects of caffeine, impulsivity, and sex on memory for word lists. *Psychology & Behavior*, *41(1)*, 25–30.

Astur, R. S., Ortiz, M. L., & Sutherland, R. J. (1998). A characterization of performance by men and women in a virtual Morris water task: A large and reliable sex difference. *Behavioral Brain Research*, *93*, 185–190.

Astur, R. S., Tropp, J., Sava, S., Constable, R. T., & Markus, E. J. (2004). Sex differences and correlations in a virtual Morris water task a virtual radial arm maze, and mental rotation. *Behavioural Brain Research*, *151*, 103–115. doi:10.1016/j.bbr.2003.08.024

Au, R., Seshardi, S., Wolf, P. A., Elias, M. F., Elias, P. K., Sullivan, L., . . . D'Agostino, R. B. (2004). New norms for a new generation: Cognitive performance in the Framingham Offspring Cohort. *Experimental Aging Research*, *30(4)*, 333–358. doi:10.1080/03610730490484380

Ayesa-Arriola, R., Rodriguez-Sanchez, J. M., Gomez-Ruiz, E., Roiz- Santiáñez, R., Reeves, L. L., & Crespo-Facorro,

B. (2014). No sex differences in neuropsychological performance in first episode psychosis patients. *Progress in Neuro-Psychopharmacology and Biological Pstchiatry*, *48*, 149–154. doi:10.1016/j.pnpbp.2013.09.009

Bacon, E. & Izaute, M. (2009). Metacognition in schizophrenia: Processes underlying patients' reflections on their own episodic memory. *Biological Psychiatry*, *66*, 1031–1037. doi:10.1016/j.biopsych.2009.07.013

Badcock, J. C., Dragovi, M., Dawson, L., & Jones, R. (2011). Normative data for rey's auditory verbal learning test in individuals with schizophrenia. *Archives of Clinical Neuroscience*, *26(3)*, 205–213. doi:10.1093/arclin/acr005

Bangirana, B., Musisi, S., Boivin, M. J., Ehnvall, A., John, C. C., Bergemann, T. L., & Allebeck, P. (2011). Malaria with neurological involvement in Ugandan children: Effect on cognitive ability, academic achievement and behaviour. *Malaria Journal*, *10:334*, 1–7. doi:10.1186/1475-2875-10-334

Banks, P. G., Dickson, A. L., & Plasay, M. T. (1987). The verbal selective reminding test: Preliminary data for healthy elderly. *Experimental Aging Research*, *13(4)*, 203–207.

Barid, T. R., Whalers, R. G., & Copper, C. K. (2007). Nonrecognition of print advertising: Emotion arousal and gender effects. *Journal of Marketing Communications*, *13(1)*, 39–51. doi:10.1080/13527260600942616

Barker-Collo, S., Clarkson, A., Cribb, A., & Grogan, M. (2010). The impact of American content on California Verbal Learning Test performance: A New Zealand illustration. *The Clinical Neuropsychologist*, *16(3)*, 290–299. doi:10.1076/clin.16.3.290.13856

Baron, I. S., Brandt, J., Ahronovich, M. D., Baker, R., Erickson, K., & Litman, F. R. (2012). Selective deficit in spatial location memory in extremely low birth weight children at age six: The PETIT study. *Child Neuropsychology: A Journal on Normal and Abnormal Development in Childhood and Adolescence*, *18(3)*, 299–311. doi:10.1080/09297049.2011.613815

Bearden, C. E., Glahn, D. C., Monkul, E. S., Barrett, J., Najt, P., Kaur, S., . . . Soares, J. C. (2006). Sources of declarative memory impairment in bipolar disorder: Mnemonic processes and clinical features. *Journal of Psychiatric Research*, *40*, 47–58. doi:10.1016/j.jpsychires.2005.08.006

Beatty, W. W. & Bruellman, J. A. (1987). Absence of gender differences in memory for map learning. *Bulletin of the Psychonomic Society*, *25(4)*, 238–239.

Becker, D. V., Kenrick, D. T., Guerin, S., & Maner, J. K. (2005). Concentrating on beauty: Sexual selection and sociospatial memory. *Personality and Social Psychology Bulletin*, *31(12)*, 1643–1652. doi:10.1177/0146167205279583

Beinhoff, U., Tumani, H., Brettschneider, J., Bittner, D., & Riepe, M. W. (2008). Gender-specificities in Alzheimer's disease and mild cognitive impairment. *Journal of Neurology*, *255(1)*, 117–122. doi:10.1007/s00415-008-0726-9

Bellace, M., Williams, J. M., Mohamed, F. B., & Faro, S. H. (2013). An fMRI study of the activation of the hippocampus by emotional memory. *International Journal of Neuroscience*, *123(2)*, 121–127. doi:10.3109/00207454.2012.742894

Bender, A. R., Naveh-Benjamin, M., & Raz, N. (2010). Associative deficit in recognition memory in a lifespan sample of healthy adults. *Psychology and Aging*, *25(4)*, 940–948. doi:10.1037/a0020595

Bengner, T., Fortmeier, C., Malina, T., Lindenau, M., Voges, B., Goebell, E., & Stodieck, S. (2006). Sex differences in face recognition memory in patients with temporal lobe epilepsy, patients with generalized epilepsy, and healthy controls. *Epilepsy & Behavior*, *9*, 593–600. doi:10.1016/j.yebeh.2006.08.021

Bernardet, U., Väljamäe, A., Inderbitzin, M., Wierenga, S., Mura, A., & Verschure, P. F. M. J. (2011). Quantifying human subjective experience and social interaction using the eXperience Induction Machine. *Brain Research Bulletin*, *85*, 305–312. doi:10.1016/j.brainresbull.2010.11.009

Beydoun, M. A., Beyboun, H. A., Kitner-Triolo, M. H., Kaufman, J. S., Evans, M. K., & Zonderman, A. B. (2013). Thyroid hormones are associated with cognitive function: Moderation by sex, race, and depressive symptoms. *The Journal of Clinical Endocrinology & Metabolism*, *98(8)*, 3470–3481. doi:10.1210/jc.2013-

1813

Bielak, A. M., Anstey, K. J., Christensen, H., & Windsor, T. D. (2012). Activity engagement is related to level, but not change in cognitive ability across adulthood. *Psychology and Aging*, *27(1)*, 219–228. doi:10.1037/a0024667

Birenbaum, M., Kelly, A. E., & Levi-Keren, M. (1994). Stimulus features and sex differences in mental rotation test performance. *Intelligence*, *19*, 51–64.

Blankevoort, C. G., Scherder, E. J. A., Wieling, M. B., Hortobágyi, T., Brouwer, W. H., Geuze, R. H., & van Heuvelen, M. J. G. (2013). Physical predictors of cognitive performance in healthy older adults: A cross-sectional analysis. *PLoS ONE*, *8(7)*, 1–9. doi:10.1371/journal.pone.0070799

Bleecker, M. L., Bolla-Wilson, K., Agnew, J., & Meyers, D. A. (1988). Age-related sex differences in verbal memory. *Journal of Clinical Psychology*, *44(3)*, 403–411.

Block, S. D., Greenberg, S. N., & Goodman, G. S. (2009). Remembrance of eyewitness testimony: Effects of emotional content, self-relevance, and emotional tone. *Journal of Applied Social Psychology*, *39(12)*, 2859–2878. doi:10.1111/j.1559-1816.2009.00553.x

Bloise, S. M. & Johnson, M. K. (2007). Memory for emotional and neutral information: Gender and individual differences in emotional sensitivity. *Memory*, *15(2)*, 192–204. doi:10.1080/09658210701204456

Boeke, C. E., Gillman, M. W., Hughes, M. D., Rifas-Shiman, S. L., Villamor, E., & Oken, E. (2012). Choline intake during pregnancy and child cognition at age 7 years. *American Journal of Epidemiology*, *177(12)*, 1338–1347. doi:10.1093/aje/kws395

Boeuf-Cazou, O., Bongue, B., Ansiau, D., Marquié, J., & Lapeyre-Mestre, M. (2011). Impact of long-term benzodiazepine use on cognitive functioning in young adults: The VISAT cohort. *European Journal of Clinical Pharmacology*, *67*, 1045–1052. doi:10.1007/s00228-011-1047-y

Boman, E. (2004). The effects of noise and gender on children's episodic and semantic memory. *Scandinavian Journal of Psychology*, *45*, 407–416. doi:10.1111/j.1467-9450.2004.00422.x

Borges, M. A. & Vaughn, L. S. (1977). Cognitive differences between the sexes in memory for names and faces. *Perceptual and Motor Skills*, *45*, 317–318.

Börsch-Supan, A. (2016a). Survey of Health, Ageing and Retirement in Europe (SHARE) wave 1. Release version: 5.0.0. SHARE-ERIC. Data set. *International Journal of Epidemiology*. doi:10.6103/SHARE.w1.500

Börsch-Supan, A. (2016b). Survey of Health, Ageing and Retirement in Europe (SHARE) wave 2. Release version: 5.0.0. SHARE-ERIC. Data set. *International Journal of Epidemiology*. doi:10.6103/SHARE.w2.500

Börsch-Supan, A. (2016c). Survey of Health, Ageing and Retirement in Europe (SHARE) wave 4. Release version: 5.0.0. SHARE-ERIC. Data set. *International Journal of Epidemiology*. doi:10.6103/SHARE.w4.500

Börsch-Supan, A. (2016d). Survey of Health, Ageing and Retirement in Europe (SHARE) wave 5. Release version: 5.0.0. SHARE-ERIC. Data set. *International Journal of Epidemiology*. doi:10.6103/SHARE.w5.500

Bowden, S. C. (1989). Maze learning: Reliability and equivalence of alternate pathways. *The Clinical Neuropsychologist*, *3(2)*, 137–144.

Bozikas, V. P., Kosmidis, M. H., Peltekis, A., Giannakou, M., Nimatoudis, I., Karavatos, A., . . . Garyfallos, G. (2010). Sex differences in neuropsychological functioning among schizophrenia patients. *Australian and New Zeeland Journal of Psychiatry*, *44*, 333–341. doi:10.3109/00048670903489833

Bracco, L., Bessi, V., Alari, F., Sforza, A., Barilaro, A., & Marinoni, M. (2011). Cerebral hemodynamic lateralization during memory tasks as assessed by functional transcranial doppler (fTCD) sonography: Effects of gender and healthy aging. *Cortex*, *47*, 750–758. doi:10.1016/j.cortex.2010.03.007

Bradbard, M. R. & Endsley, R. C. (1983). The effects of sex-typed labeling on preschool children's information-seeking and retention. *Sex Roles*, *9(2)*, 247–260.

Bradbard, M. R., Martin, C. L., Endsley, R. C., & Halverson, C. F. (1986). Influences of sex stereotypes on children's exploration and memory. a competence versus performance distinction. *Developmental Psychology*, *22(4)*, 481–486.

Brevik, E. J., Eikeland, R. A., & Lundervold, A. J. (2013). Subthreshold depressive symptoms have a negative

impact on cognitive functioning in middle-aged and older males. *Frontiers in Psychology*, *4*, Article 309. doi:10.3389/fpsyg.2013.00309

Brewster, P. W. H., Mullin, C. R., Dobrin, R. A., & Steeves, J. K. E. (2011). Sex differences in face processing are mediated by handedness and sexual orientation. *Laterality: Asymmetries of Body, Brain and Cognition*, *16(2)*, 188–200. doi:10.1080/13576500903503759

Brindal, E., Baird, D., Slater, A., Danthii, V., Wilson, C., Bowen, J., & Noakes, M. (2012). The effect of beverages varying in glycaemic load on postprandial glucose responses, appetite and cognition in 10-12-year-old school children. *British Journal of Nutrition*, *110*, 529–537. doi:10.1017/S0007114512005296

Bringmann, M. W., Tyler, K. E., McAhren, P. E., & Bringmann, W. G. (1989). A successful and unsuccessful replication of William Stern's eyewitness research. *Perceptual and Motor Skills*, *69*, 619–625.

Brito, G. N. O., Alfradique, G. M. N., Pereia, C. C. S., Porto, C. M. B., & Santos, T. R. (1998). Developmental norms for eight instruments used in the neuropsychological assessment of children: Studies in Brazil. *Brazilian Journal of Medical and Biological Research*, *31*, 399–412.

Brodsky, M. B., McNeil, M. M., Doyle, P. J., Fossett, T. R. D., Timm, N. H., & Park, G. H. (2003). Auditory serial position effects in story retelling for non-brain-injured participants and persons with aphasia. *Journal of Speech, Language and Hearing Research*, *46*, 1124–1137. doi:10.1044/1092-4388(2003/088)

Brønnick, K., Alves, G., Aarsland, D., Tysnes, O.-B., & Larsen, J. P. (2011). Verbal memory in drug-naive, newly diagnosed Parkinson's disease. The retrieval deficit hypothesis revisited. *Neuropsychology*, *25(1)*, 114–124. doi:10.1037/a0020857

Brooking, L., Uehara, E., Charchat-Fichman, H., & Landeira-Fernandez, J. (2012). Memory performance in Brazilian school-age children. *Psychology & Neuroscience*, *5 (2)*, 165–173. doi:10.3922/j.psns.2012.2.06

Brown, F. C., Roth, R. M., Saykin, A. J., & Beverly-Gibson, G. (2007). A new measure of visual location learning and memory: Development and psychometric properties for the Brown Location Test (BLT). *The Clinical Neuropsychologist*, *21(5)*, 811–825. doi:10.1080/13854040600878777

Bruck, M. (2009). Human figure drawings and children's recall of touching. *Journal of Experimental Psychology: Applied*, *15(4)*, 361–374. doi:10.1037/a0017120

Buchanan, T. W. & Tranel, D. (2008). Stress and emotional memory retrieval: Effects of sex and cortisol response. *Neurobiology of Learning and Memory*, *89(2)*, 134–141. doi:10.1016/j.nlm.2007.07.003

Buchmann, A., Mondadori, C. R. A., Hänggi, J., Aerni, A., Vrticka, P., de Quervain, J. F., . . . Henke, K. (2008). Prion protein M129V polymorphism affects retrieval-related brain activity. *Neuropsychologia*, *48*, 2389–2402. doi:10.1016/j.neuropsychologia.2008.03.002

Bücker, J., Popuri, S., Muralidharan, K., Kozicky, J.-M., Baitz, H. A., Honer, W. G., . . . Yatham, L. N. (2014). Sex differences in cognitive functioning in patients with bipolar disorder who recently recovered from a first episode of mania: Data from the Systematic Treatment Optimization Program for Early Mania (STOP-EM). *Journal of Affective Disorders*, *155*, 162–168. doi:10.1016/j.jad.2013.10.044

Burdick, K. E., Robinson, D. G., Malhotra, A. K., & Szeszko, P. R. (2008). Neurocognitive profile analysis in obsessive-compulsive disorder. *Journal of the International Neuropsychological Society*, *14*, 640–645. doi:10.10170S1355617708080727

Butts, S. J., Mixon, K. D., Mulekar, M. S., & Bringmann, W. G. (1995). Gender differences in eyewitness testimony. *Perceptual and Motor Skills*, *80(1)*, 59–63.

Cadar, D., Pikhart, H., Mishra, G., Stephen, A., Kuh, D., & Richards, M. (2012). The role of lifestyle behaviors on 20-year cognitive decline. *Journal of Aging Research*, *2012*, 1–13. doi:10.1155/2012/304014

Cahill, L., Haier, R. J., White, N. S., Fallon, S., Kilpatrick, L., Lawrence, C., . . . Alkire, M. T. (2001). Sex-related difference in amygdala activity during emotionally influenced memory storage. *Neurobiology of Learning and Memory*, *75*, 1–9. doi:10.1006/nlme.2000.3999

Campeanu, S., Craik, F. I. M., & Alain, C. (2013). Voice congruency facilitates word recognition. *PLoS ONE*, *8(3)*, 1–9. doi:10.1371/journal.pone.0058778

Canli, T., Desmond, J. E., Zhao, Z., & Gabrieli, J. D. E. (2002). Sex differences in the neural basis of emotional

memories. *Proceedings of the National Academy of Sciences of the United States of America*, *99(16)*, 10789–10794. doi:10.1073/pnas.162356599

Cánovas, R., Léon, I., Serrano, P., Roldán, M. D., & Cimadevilla, J. M. (2011). Spatial navigation impairment in patients with refractory temporal lobe epilepsy: Evidence from a new virtual reality-based task. *Epilepsy & Behavior*, *22*, 364–369. doi:10.1016/j.yebeh.2011.07.021

Cansino, S., Estrada-Manilla, C., Hernández-Ramos, E., Martínez-Galindo, J. G., Torres-Trejo, F., Gómez-Fernández, T., . . . Rodríguez-Ortiz, M. (2012). The rate of source memory decline across the adult life span. *Developmental Psychology*, *49(5)*, 973–985. doi:10.1037/a0028894

Caplan, L. J. & Lipman, P. D. (1995). Age and gender differences in the effectiveness of map-like learning aids in memory for routes. *The Journals of Gerontology: Series B: Psychological sciences and social sciences. 50(3)*, 126–133.

Carlson, L. E. & Sherwin, B. B. (1998). Steroid hormones, memory and mood in a healthy elderly population. *Psychoneuroendocrinology*, *23 (6)*, 583–603.

Carnero-Pardo, C., Sáez-Zea, C., De la Vega Cotarelo, R., & Gurpegui, M. (2012). FOTOTRANS Study: Multicentre study on the validity of Fototest under clinical practice conditions. *Neurología*, *27(2)*, 68–75. doi:10.1016/j.nrl.2011.06.001

Carrus, D., Christodoulou, T., Hadjulis, M., Haldane, M., Galea, A., Koukopoulos, A., . . . Frangou, S. (2010). Gender differences in immediate memory in bipolar disorder. *Psychological Medicine*, *40(8)*, 1349–1355. doi:10.1017/S0033291709991644

Casella, S., Zanini, B., Lanzarotto, F., Ricci, C., Marengoni, A., Romanelli, G., & Lanzini, A. (2012). Cognitive performance is impaired in coeliac patients on gluten free diet: A case-control study in patients older than 65 years of age. *Digestive and Liver*

Disease, *44(9)*, 729–735. doi:10.1016/j.dld.2012.03.008 Caselli, R. J., Dueck, A. C., Locke, D. E. C., Hoffman-Snyder, C. R., Woodruff, B. K.,

Rapcsak, S. Z., & Reiman, E. M. (2011). Longitudinal modeling of frontal cognition in APOE ε4 homozygotes, heterozygotes, and noncarriers. *Neurology*, *76*, 1383–1388. doi:10.1212/WNL.0b013e3182167147

Cashdan, E., Marlowe, F. W., Crittenden, A., Porter, C., & Wood, B. M. (2012). Sex differences in spatial cognition among Hadza foragers. *Evolution and Human Behavior*, *33(4)*, 274–284. doi:10.1016/j.evolhumbehav.2011.10.005

Casiere, D. A. & Ashton, N. L. (1996). Eyewitness accuracy and gender. *Perceptual and Motor Skills*, *83(3)*, 914.

Cavalieri, M., Ropele, S., Petrovic, K., Pluta-Fuerst, A., Homayoon, N., Enzinger, C., . . . Schmidt, R. (2010). Metabolic syndrome, brain magnetic resonance imaging, and cognition. *Diabetes Care*, *33(12)*, 2489–2495. doi:10.2337/dc10-0851

Chai, X. J. & Jacobs, L. F. (2009). Effects of cue types on sex differences in human spatial memory. *Behavioural Brain Research*, *208*, 336–342. doi:10.1016/j.bbr.2009.11.039

Chaill, L., Haier, R. J., White, N. S., Fallon, J., Kilpatrick, L., Lawrence, C., . . . Alkire, M. T. (2001). Sex-related difference in amygdala activity during emotionally influenced memory storage. *Neurobiology of Learning and Memory*, *75*, 1–9. doi:10.1006/nlme.2000.3999

Chapman, R. M., Mapstone, M., Gardner, M. N., Sandoval, T. C., McCrary, J. W., Guillily, M. D., . . . DeGrush, E. (2011). Women have farther to fall: Gender differences between normal elderly and Alzheimer's disease in verbal memory engender better detection of Alzheimer's disease in women. *Journal of the International Neuropsychological Society*, *17(4)*, 654–662. doi:10.1017/S1355617711000452

Chavez, E. L., Schwartz, M. M., & Brandron, A. (1982). Effects of sex of subject and method of block presentation on the tactual performance test. *Journal of Consulting and Clinical Psychology*, *50(4)*, 600–601. doi:10.1037//0022-006X.50.4.600

Cherney, I. D. (2005). Children's and adults' recall of sex- stereotyped toy pictures: Effects of presentation and memory task. *Indand and Child Development*, *14*, 11–27. doi:10.1002/icd.372

Cherney, I. D. & Ryalls, B. O. (1999). Gender-linked differences in the incidental memory of children and adults.

Journal of Experimental Child Psychology, *72*, 305–328.

Chin, T. & Rickard, N. S. (2010). Nonperformance, as well as performance, based music engagement predicts verbal recall. *Music Perception*, *27(3)*, 197–208. doi:10.1525/MP.2010.27.3.197

Chipman, K. & Kimura, D. (1998). An investigation of sex differences on incidental memory for verbal and pictorial material. *Learning and Individual Differences*, *10(4)*, 259–272.

Choi, J. & L'Hirondell, N. (2005). Object location memory: A direct test of the verbal memory hypothesis. *Learning and Individual Differences*, *15(3)*, 237–245. doi:10.1016/j.lindif.2005.02.001

Choi, J. & Silverman, I. (2003). Processes underlying sex differences in route-learning strategies in children and adolescents. *Personality and Individual Differences*, *34(7)*, 1153–1166. doi:10.1016/S0191-8869(02)00105-8

Choudhury, E. S., Moberg, P., & Doty, R. L. (2003). Influences of age and sex on a microencapsulated odor memory test. *Chemical Senses*, *28(9)*, 799–805. doi:10.1093/chemse/bjg072

Christensen, H., Korten, A. E., Mackinnon, A. J., Jorm, A. F., Hendersen, A. S., & Rogers, B. (2000). Are changes in sensory disability, reaction time, and grip strength associated with changes in memory and crystallized intelligence? *Gerontology*, *46*, 276–292.

Christensen, H., Mackinnon, A. J., Korten, A., & Jorm, A. F. (2001). The "common cause hypothesis" of cognitive aging: Evidence for not only a common factor but also specific associations of age with vision and grip strength in a cross-sectional analysis. *Psychology and Aging*, *16(4)*, 588–599. doi:10.1037/0882-7974.16.4.588

Christensen, H., Mackinnon, A., Jorm, A. F., Korten, A., Jacomb, P., Hofer, S. M., & Henderson, S. (2004). The Canberra Longitudinal Study: Design, aims, methodology, outcomes and recent empirical investigations. *Aging, Neuropsychology, and Cognition: A Journal on Normal and Dysfunctional Development*, *11(2-3)*, 169–195. doi:10.1080/13825580490511053

Cinan, S., Atalay, D., Sisman, S., Basbug, G., Dervent-Ozbek, S., Teoman, D. D., . . . Yurtsever, O. D. (2007). Memory for object locations: Priority effect and sex differences in associative spatial learning. *Learning and Motivation*, *38*, 326–341. doi:10.1016/j.lmot.2007.01.002

Clare, L., Wilson, B. A., Emslie, H., Tate, R., & Watson, P. (2000). Adapting the Rivermead Behavioural Memory Test Extended Version (RBMT-E) for people with restricted mobility. *British Journal of Clinical Psychology*, *39*, 363–369.

Clark, D. M. & Teasdale, J. D. (1985). Constraints on the effects of mood on memory. *Journal of Personality and Social Psychology*, *48(6)*, 1595–1608.

Cockroft, K. & Blackburn, M. (2008). The relationship between Senior South African Individual Scale - Revised (SSAIS-R) and subtest of reading ability. *South African Journal of Psychology*, *38*, 377–389. doi:10.1177/008124630803800209

Collaer, M. L. & Evans, J. R. (1982). A measure of short-term visual memory based on the WISC-R coding subtest. *Journal of Clinical Psychology*, *38(3)*, 641–644.

Colley, A., Ball, J., Kirby, N., Harvey, R., & Vingelen, I. (2002). Gender-linked differences in everyday memory performance: Effort makes the difference. *Sex Roles*, *47(11)*, 577–582. doi:10.1023/A:1022082023501

Collie, A., Shafiq-Antonacci, R., Maruff, P., Tyler, P., & Currie, J. (1999). Norms and the effects of demographic variables on a neuropsychological battery for use in healthy ageing Australian populations. *Australian and New Zealand Journal of Psychiatry*, *33(4)*, 568–575.

Coluccia, E., Gamboz, N., & Brandimonte, M. A. (2011). Normative data for a battery of free recall, cued recall and recognition tests in the elderly Italian population. *Neurological Sciences*, *32(6)*, 1103–1114. doi:10.1007/s10072-011-0747-5

Comijs, H. C., Gerritsen, L., Penninx, B. W., Bremmer, M. A., Deeg, D. J., & Geerlings, M. I. (2010). The association between serum cortisol and cognitive decline in older persons. *American Journal of Geriatric Psychiatry*, *18(1)*, 42–50. doi:10.1097/JGP.0b013e3181b970ae

Conde-Sala, J. L., Garre-Olmo, J., Vilalta-Franch, J., Llinàs-Reglà, J., Turró-Garriga, O., Lozano-Gallego, M., . . .

López-Pousa, S. (2012). Predictors of cognitive decline in Alzheimer's disease and mild cognitive impairment using the CAMCOG: A five-year follow-up. *International Psychogeriatrics*, *24(6)*, 948–958. doi:10.1017/S1041610211002158

Corbeil, M.-E. M. & McKelvie, S. J. (2008). Pornography use and recall of sexual and neutral words. *North American Journal of Psychology*, *10(2)*, 1–12.

Corrêa, M. S., Balardin, J. B., Caldieraro, M. A. K., Fleck, M. P., Argimon, I., Luz, C., & Bromberg, E. (2012). Contextual recognition memory deficits in major depression are suppressed by cognitive support at encoding. *Biological Psychology*, *89*, 293–299. doi:10.1016/j.biopsycho.2011.11.001

Costa, P. S., Santos, N. C., Cunha, P., Almeida, J., & Sousa, N. (2013). The use of Bayesian latent class cluster models to classify patterns of cognitive performance in healthy ageing. *PLoS ONE*, *8(8)*, 1–8. doi:10.1371/journal.pone.0071940

Cox, D. & Waters, H. S. (1986). Sex differences in the use of organiation strategies: A developmental analysis. *Journal of Experimental Child Psychology*, *41(1)*, 18–37.

Crook, T. H., III, Youngjohn, J. R., & Larrabee, G. J. (1990). The Misplaced Object Test: A measure of everyday visual memory. *Journal of Clinical and Experimental Neuropsychology*, *12(6)*, 819–833.

Crook, T. H., Youngjohn, J. R., & Larrabee, G. J. (1993). The influence of age, gender, and cues on computer-simulated topographic memory. *Developmental Neuropsychology*, *9(1)*, 41–53.

Crotty, K. C., Ahronovich, M. D., Baron, I. S., Baker, R., Erickson, K., & Litman, F. R. (2012). Neuropsychological and behavioral effects of postnatal dexamethasone in extremely low birth weight preterm children at early school age. *Journal of Perinatology*, *32*, 139–146. doi:10.1038/jp.2011.62

Cruse, D. & Critchlow Leigh, B. (1987). ''adam's rib" revisited: Legal and non-legal influences on the the processing of trial testimony. *Social Behavior*, *2*, 221–230.

Cserjesi, R., Van Braeckel, K., Butcher, P. R., Kerstjens, J. M., Reijneveld, S. A., Bouma, A., . . . Bos, A. F. (2012). Functioning of 7-year-old children born at 32 to 35 weeks' gestational age. *Pediatrics*, *130(4)*, e838–e846. doi:10.1542/peds.2011-2079

Curry, J. F., Logue, P. F., & Butler, B. (1986). Child and adolescent norms for Russell's revision of the Wechsler Memory Scale. *Journal of Clinical Child Psychology*, *15(3)*, 214–220.

Daalman, K., van Zandvoort, M., Bootsman, F., Boks, M., Kahn, R., & Sommer, I. (2011). Auditory verbal hallucinations and cognitive functioning in healthy individuals. *Schizophrenia Research*, *132*, 203–207. doi:10.1016/j.schres.2011.07.013

Dabbs, J. M., Jr., Chang, E.-L., Strong, R. A., & Milun, R. (1998). Spatial ability, navigation strategy, and geographic knowledge among men and women. *Evolution and Human Behaviour*, *19(2)*, 89–98.

Daprati, E., Nico, D., Delorme, R., Leboyer, M., & Zalla, T. (2013). Memory for past events: Movement and action chains in high-functioning autism spectrum disorders. *Experimential Brain Research*, *226*, 325–334. doi:0.1007/s00221-013-3436-1

Davey, A., Dai, T., Woodard, J. L., Miller, L. S., Gondo, Y., Johnson, M. A., . . . Centenarian, G. (2013). Profiles of cognitive functioning in a population-based sample of centenarians using factor mixture analysis. *Experimental Aging Research*, *39(2)*, 125–144. doi:10.1080/0361073X.2013.761869

Davidson, D. (2006). The role of basic, self-conscious and self-conscious evaluative emotions in children's memory and understanding of emotion. *Motivation and Emotion*, *30(3)*, 237–247. doi:10.1007/s11031-006-9037-6

De Goede, M. & Postma, A. (2008). Gender differences in memory for objects and their locations: A study on automatic versus controlled encoding and retrieval contexts. *Brain and Cognition*, *66(3)*, 232–242. doi:10.1016/j.bandc.2007.08.004

de Bartolomeis, A., Balletta, R., Giordano, S., Filomena Buonaguro, E., Latte, G., & Iasevoli, F. (2013). Differential cognitive performances between schizophrenic responders and non-responders to antipsychotics: Correlation with course of the illness, psychopathology, attitude to the treatment and antipsychotics doses. *Psychiatry Research*, *210*, 387–395. doi:10.1016/j.psychres.2013.06.042

DeFries, J. C., Corley, R. P., Johnson, R. C., Vandenberg, S. G., & Wilson, J. R. (1982). Sex-by-generation and ethnic group-by-generation interactions in the Hawaii Family Study of Cognition. *Behavior Genetics*, *12(2)*, 223–230.

Deloire, M. S. A., Salort, E., Bonnet, M., Arimone, Y., Boudineau, M., Amieva, H., . . . Brochet, B. (2005). Cognitive impairment as marker of diffuse brain abnormalities in early relapsing remitting multiple sclerosis. *Journal of Neurology, Neurosurgery & Psychiatry*, *76(4)*, 519–526. doi:10.1136/jnnp.2004.045872

den Heijer, T., Geerlings, M. I., Hofman, A., de Jong, F. H., Launer, L. J., Pols, H. A. P., & Breteler, M. M. B. (2003). Higher estrogen levels are not asscoiated with larger hippocampi and better memory performance. *Archives of Neurology*, *60*, 210–220.

Dennett, H. W., McKone, E., Tavashmi, R., Hall, A., Pidcock, M., Edwards, M., & Duchaine, B. (2012). The Cambridge Car Memory Test: A task matched in format to the cambridge face memory test, with norms, reliability, sex differences, dissociations from face memory, and expertise effects. *Behavior Research Methods*, *44(2)*, 587–605. doi:10.3758/s13428-011-0160-2

Dewhurst, S. A., Anderson, R. J., & Knott, L. M. (2012). A gender difference in the false recall of negative words: Women DRM more than men. *Cognition and Emotion*, *26(1)*, 65–74. doi:10.1080/02699931.2011.553037

Diege, N., Maahr, E., & Backenroth-Ohsako, G. (2010). Reduced capacity in a dichotic memory test for adult patients with ADHD. *Journal of Attention Disorders*, *13(6)*, 677–683. doi:10.1177/1087054709347245

Dingwall, K. M., Lewis, M. S., Maruff, P., & Cairney, S. (2010). Assesing cognition following petrol sniffing for indigenous Australians. *Australian and New Zealand Journal of Psychiatry*, *44(7)*, 631–639. doi:10.3109/00048671003627405

Dodrill, C. B. (1979). Sex differences on the Halstead-Reitan Neuropsychological Battery and on other neuropsychological measures. *Journal of Clinical Psychology*, *35(2)*, 236–341.

Doty, R. L. & Kerr, K.-L. (2005). Episodic odor memory: Influences of handedness, sex, and side of nose. *Neuropsychologia*, *43(12)*, 1749–1753. doi:10.1016/j.neuropsychologia.2005.02.007

Drakeford, J. L., Edelstyn, N. M. J., Oyebode, F., Srivastava, S., Calthorpe, W. R., & Mukherjee, T. (2010). Recollection deficiencies in patients with major depressive disorder. *Psychiatry Research*, *175*, 205–210. doi:10.1016/j.psychres.2008.08.010

Duff, K., Schoenberg, M. R., Mold, J. W., Scott, J. G., & Adams, R. L. (2010). Gender differences on the Repeatable Battery for the Assessment of Neuropsychological Status subtests in older adults: Baseline and retest data. *Journal of Clinical and Experimental Neuropsychology*, *33(4)*, 448–455. doi:10.1080/13803395.2010.533156

Eals, M. & Silverman, I. (1994). The hunter-gatherer theory of spatial sex differences: Proximate factors mediating the female advantage in recall of object arrays. *Ethology and Sociobiology*, *15(2)*, 95–105. doi:10.1016/0162-3095(94)90020-5

Economou, A. (2009). Memory score discrepancies by healthy middle-aged and older individuals: The contributions of age and education. *Journal of the International Neuropsychological Society*, *15(6)*, 963–972. doi:10.1017/S1355617709990580

Economou, A., Papageorgiou, S., & Karageorgiou, C. (2006). Working-delayed memory difference detects mild cognitive impairment without being affected by age and education. *Journal of Cinical and Experimental Neuropsychology*, *28(4)*, 528–535. doi:10.1080/13803390590949340

Ecuyer-Dab, I. & Robert, M. (2004). Spatial ability and home-range size: Examining the relationship in western men and women (homo sapiens). *Journal of Comparative Psychology*, *118(2)*, 217–231. doi:10.1037/0735-7036.118.2.217

Edelstein, S. L., Kritz-Silverstein, D., & Barrett-Connor, E. (1998). Prospective association of smoking and alcohol use with cognitive function in an elderly cohort. *Journal of Women's Health*, *7(10)*, 1271–1281.

Eidelman, S., Crandall, C. S., Goodman, J. A., & Blanchar, J. C. (2012). Low-effort thought promotes political conservatism. *Personality and Social Psychology Bulletin*, *38(6)*, 808–820. doi:10.1177/0146167212439213

Elamin, M., Phukan, J., Bede, P., Jordan, N., Byrne, S., Pender, N., & Hardiman, O. (2011). Executive dysfunction is a negative prognostic indicator in patients with ALS without dementia. *Neurology*, *76(14)*, 1263–1269. doi:10.1212/WNL.0b013e318214359f

Elias, M. F., Elias, P. K., D'Agostino, R. B., Silbershatz, H., & Wolf, P. A. (1997). Role of age, education, and gender on cognitive performance in the Framingham Heart Study: Community-based norms. *Experimental Aging Research*, *23(3)*, 201–235. doi:10.1080/03610739708254281

Ellis, A. K., Bush, A. I., Darby, D., De Fazio, D., Foster, J., Hudson, P., . . . Ames, D. (2009). The Australian Imaging, Biomarkers and Lifestyle (AIBL) study of aging: Methodology and baseline characteristics of 1112 individuals recruited for a longitudinal study of Alzheimer's disease. *International psychogeriatrics*, *21(4)*, 672–687. doi:doi:10.1017/S1041610209009405

Ellis, H., Shepherd, J., & Bruce, A. (1973). The effects of age and sex upon adolescents' recognition of faces. *The Journal of Genetic Psychology*, *123*, 173–174.

Epstein, M. L. (1974). Sex differences in incidental learning and recall of related and unrelated word pairs. *The Journal of Psychology*, *88*, 3–8.

Ernest, C. E. (1983). Imagery and verbal ability and recognition memory for pictures and words in males and females. *Educational Psychology*, *3(3-4)*, 227–244.

Espin, L., Almela, M., Hildago, V., Villada, C., Salvador, A., & Gomez-Amor, J. (2013). Acute pre-learning stress and declarative memory: Impact of sex, cortisol response and menstrual cycle phase. *Hormones and Behavior*, *63*, 759–765. doi:10.1016/j.yhbeh.2013.03.013

Evardone, M. & Alexander, G. M. (2009). Anxiety, sex-linked behaviors, and digit ratios (2D:4D). *Archives of Sexual Behavior*, *38*, 442–455. doi:10.1007/s10508-007-9260-6

Fein, G. & McGillivray, S. (2007). Cognitive performance in long-term abstint elderly alcoholics. *Alcoholism: Clinical and Experimental Research*, *31(11)*, 1788–1799. doi:10.1111/j.1530-0277.2006.00185.x

Fein, G., Torres, J., Price, L. J., & Di Sclafani, V. (2006). Cognitive performance in long-term abstinent alcoholic individuals. *Alcoholism: Clinical and Experimental Research*, *30(9)*, 1538–1544. doi:10.1111/j.1530-0277.2006.00185.x

Felmingham, K. L., Tran, T. P., Fong, W. C., & Bryant, R. A. (2012). Sex differences in emotional memory consolidation: The effect of stress-induced salivary alphe-amylase and cortisol. *Biological Psychology*, *89(3)*, 539–544. doi:10.1016/j.biopsycho.2011.12.006

Ferguson, C. J., Cruz, A. M., Martinez, D., Rueda, S. M., & Ferguson, D. E. (2010). Violence and sex as advertising strategies in television commercials. *European Psychologist*, *15(4)*, 304–311. doi:10.1027/1016-9040/a000016

Ferguson, C. J., Cruz, A. M., & Rueda, S. M. (2008). Gender, video game playing habits and visual memory tasks. *Sex Roles*, *58(3-4)*, 279–286. doi:10.1007/s11199-007-9332-z

Fernandez-Mendoza, J., Calhoun, S., Bixler, E. O., Pejovic, S., Karataraki, M., Liao, D., . . . Vgontzas, A. N. (2009). Insomnia with objective short sleep duration is associated with deficits in neuropsychological performance: A general population study. *Sleep and Performance*, *33(4)*, 459–465.

Ferree, N. K. & Cahill, L. (2009). Post-event spontaneous intrusive recollections and strength of memory for emotional events in men and women. *Consciousness and Cognition*, *18*, 126–134. doi:10.1016/j.concog.2008.11.008

Fichman, H. C., Dias, L. B. T., Fernandes, C. S., Lourenço, R., Caramelli, P., & Nitrini, R. (2010). Normative data and construct validity of the Rey Auditory Verbal Learning Test in a Brazilian elderly population. *Psychology & Neuroscience*, *3(1)*, 79–84. doi:10.3922/j.psns.2010.1.010

File, S. E., Fluck, E., & Leahy, A. (2001). Nicotine has calming effects on stress-induced mood changes in females, but enhances aggressive mood in males. *International Journal of Neuropsychopharmacology*, *4(4)*, 371–376. doi:10.1017/S1461145701002577

Fillenbaum, G. G., Burchette, B. M., Unverzagt, F. W., Rexroth, D. F., & Welsh-Bohmer, K. (2011). Norms for cerad constructional praxis recall. *The Clinical Neuropsychologist*, *25(8)*, 1345–1358.

doi:10.1080/13854046.2011.614962
Findlay, L., Bernier, J., Tuokko, H., Kirkland, S., & Gilmour, H. (2009). Validation of cognitive functioning categories in the Canadian Community Health Survey-Healthy Aging. *Health Reports*, *21(4)*, 85–100.
Fischer, H., Sandblom, J., Nyberg, L., Herlitz, A., & Bäckman, L. (2007). Brain activation while forming memories of fearful and neutral faces in women and men. *Emotion*, *7(4)*, 767–773. doi:10.1037/1528-3542.7.4.767
Fiske, A. & Gatz, M. (2007). The Apartment Test: Validity of a memory measure. *Aging, Neuropsychology, and Cognition*, *14*, 441–461. doi:10.1080/13825580600611294
Flegr, J., Hampl, R., ernochová, D., Preiss, M., Biíková, M., Sieger, L., . . . Klose, J. (2012). The relation of cortisol and sex hormone levels to results of psychological, performance, iq and memory tests in military men and women. *Neuroendocrinology Letters*, *33(2)*, 224–235.
Foti, F., Menghini, D., Petrosini, L., Valerio, G., Crinò, A., Vicari, S., . . . Mandolesi, L. (2011). Spatial competences in PraderWilli syndrome: A radial arm maze study. *Behavior Genetics*, *41*, 445–456. doi:10.1007/s10519-011-9471-4
Frasson, P., Ghiretti, R., Catricalà, E., Pomati, S., Marcone, A., Parisi, L., . . . Clerici, F. (2011). Free and Cued Selective Reminding Test: An Italian normative study. *Neurological Sciences*, *32(6)*, 1057–1062. doi:10.1007/s10072-011-0607-3
Fried, C. B. & Johanson, J. C. (2008). Sexual and violent media's inhibition of advertisement memory: Effect or artifact? *Journal of Applied Social Psychology*, *38(7)*, 1716–1735. doi:10.1111/j.1559-1816.2008.00366.x
Friedman, M. A., Schinka, J. A., Mortimer, J. A., & Graves, A. B. (2002). Hopkins Verbal Learning Test-Revised: Norms for elderly African Americans. *The Clinical Neuopsychologist*, *16(3)*, 356–372. doi:10.1076/clin.16.3.356.13857
Frings, L., Wagner, K., Unterrainer, J., Spreer, J., Halsband, U., & Schulze-Bonhage, A. (2006). Gender-related differences in lateralization of hippocampal activation and cognitive strategy. *NeuroReport: For Rapid Communication of Neuroscience Research*, *17(4)*, 417–421. doi:10.1097/01.wnr.0000203623.02082.e3
Friswell, J., Phillips, C., Holding, J., Morgan, C. J. A., Brandner, B., & Curran, H. V. (2008). Acute effects of opioids on memory functions of healthy men and women. *Psycopharmacology*, *198*, 243–250. doi:0.1007/s00213-008-1123-x
Fritsch, T., Larsen, J. D., & Smyth, K. A. (2007). The role of adolescent IQ and gender in the use of cognitive support for remembering in aging. *Aging, Neuropsychology, and Cognition: A Journal on Normal and Dysfunctional Development*, *14(4)*, 394–416. doi:10.1080/13825580500473696
Fuentes, A., Collins, D. L., Garcia-Lorenzo, D., Sled, J. G., Narayanan, S., Arnold, D. L., . . . Till, C. (2012). Memory performance and normalized regional brain volumes in patients with pediatric-onset multiple sclerosis. *Journal of the International Neuropsychological Society*, *18*, 471–480. doi:10.1017/S1355617711001913
Fulda, S., Beitinger, M. E., Reppermund, S., Winkelmann, J., & Wetter, T. C. (2010). Short-term attention and verbal fluency is decreased in restless legs syndrome patients. *Movement Disorders*, *25(15)*, 2641–2648. doi:10.1002/mds.23353
Gale, C. R., Martyn, C. N., Mariott, L. D., Limond, J., Crozier, S., Inskip, H. M., . . . Robinson, S. M. (2008). Dietary patterns in infancy and cognitive and neuropsychological function in childhood. *Journal of Child Psychology and Psychiatry*, *50(7)*, 816–823. doi:10.1111/j.1469-7610.2008.02029.x
Gale, S. D., Baxter, L., Connor, D. J., Herring, A., & Comer, J. (2007). Sex differences on the Rey Auditory Verbal Learning Test and the Brief Visuospatial Memory TestRevised in the elderly: Normative data in 172 participants. *Journal of Clinical and Experimental Neuropsychology*, *29(5)*, 561–567. doi:10.1080/13803390600864760
Galea, L. A. M. & Kimura, D. (1993). Sex differences in route-learning. *Personality and individual Differences*, *14 (1)*, 53–65.
Gallagher, C. & Burke, T. (2007). Age, gender and IQ effects on the Rey-Osterrieth Complex Figure Test. *British Journal of Clinical Psychology*, *46(1)*, 35–45. doi:10.1348/014466506X106047
Gallagher, P., Neave, N., Hamilton, C., & Gray, J. M. (2006). Sex differences in object location memory: Some

further methodological considerations. *Learning and Individual Differences*, *16*, 277–290. doi:10.1016/j.lindif.2006.12.007

Ganguli, M., Snitz, B., Vander Bilt, J., & Chang, C. H. (2009). How much do depressive symptoms affect cognition at the population level? The MonongahelaYoughiogheny Healthy Aging Team (MYHAT) study. *International Journal of Geriatric Psychiatry*, *24*, 1277–1284. doi:10.1002/gps.2257

Gavazzeni, J., Andersson, T., Bäckman, L., Wiens, S., & Fischer, H. (2012). Age, gender, and arousal in recognition of negative and neutral pictures 1 year later. *Psychology and Aging*, *27(4)*, 1039–1052. doi:10.1037/a0027946

Gedney, J. J. & Logan, H. (2004). Memory for stress-associated acute pain. *The Journal of Pain*, *5(2)*, 83–91. doi:10.1016/j.jpain.2003.11.005

Geer, J. H. & McGlone, M. S. (1990). Sex differences in memory for erotica. *Cognition and Emotion*, *4(1)*, 71–78.

Gerstorf, D., Herlitz, A., & Smith, J. (2006). Stability of sex differences in cognition in advanced old age: The role of education and attrition. *Journal of Gerontology*, *61B(4)*, 245–249.

Gibbs, A. C. & Wilson, J. F. (1999). Sex differences in route learning by children. *Perceptual and Motor Skills*, *88(2)*, 590–594.

Glaser, E., Mendrek, A., Germaine, M., Lakis, N., & Lavoie, M. E. (2012). Sex differences in memory of emotional images: A behavioral and electrophysiological investigation. *International Journal of Psychophysiology*, *85*, 17–26. doi:10.1016/j.ijpsycho.2012.01.007

Göder, R., Boigs, M., Braun, S., Friege, L., Fritzer, G., Aldenhoff, J. B., & Hinze-Selch, D. (2004). Impairment of visuospatial memory is associated with decreased slow wave sleep in schizophrenia. *Journal of Psychiatric Research*, *38(6)*, 591–599. doi:10.1016/j.jpsychires.2004.04.005

Gogos, A., Joshua, N., & Rossell, S. L. (2010). Use of the Repeatable Battery for the Assessment of Neuropsychological Status (RBANS) to investigate group and gender differences in schizophrenia and bipolar disorder. *Australian and New Zealand Journal of Psychiatry*, *44(3)*, 220–229. doi:10.3109/00048670903446882

Goldstein, J. M., Seidman, L. J., Goodman, J. M., Koren, D., Lee, H., Weintraub, S., & Tsuang, M. T. (1998). Are there sex differences in neuropsychological functions among patients with schizophrenia? *American Journal of Psychiatry*, *155(10)*, 1358–1364.

Golier, J. A., Yehuda, R., De Santi, S., Segal, S., Dolan, S., & de Leon, M. J. (2005). Absence of hippocampal differences in survivors of the Nazi Holocaust with and without posttraumatic stress disorder. *Psychiatry Research: Neuroimaging*, *139*, 53–64. doi:10.1016/j.pscychresns.2005.02.007

Gonzales, M. M., Tarumi, T., Eagan, D. E., Tanaka, H., Vaghasia, M., & Haley, A. P. (2012). Indirect effects of elevated body mass inde on memory performance through altered cerebral metabolite concentrations. *Psychosomatic Medicine*, *74(7)*, 691–698. doi:10.1097/PSY.0b013e31825ff1de

González, H. M., Mungas, D., Reed, B. R., Marshall, & Haan, M. N. (2001). A new verbal learning and memory test for English- and Spanish-speaking older people. *Journal of the International Neuropsychological Society*, *7(5)*, 544–555. doi:10.1017/S1355617701755026

Gow, A., Corley, J., Starr, J., & Deary, I. J. (2013). Which social network or support factors are associated with cognitive abilities in old age? *Gerontology*, *59(5)*, 454–463. doi:10.1159/000351265

Grabe, M. E. & Kamhawi, R. (2006). Hard wired for negative news? Gender differences in processing broadcast news. *Communication Research*, *33(5)*, 346–369. doi:10.1177/0093650206291479

Grabe, M. E. & Samson, L. (2011). Sexual cues emanating from the anchorette chair: Implications for perceived professionalism, fitness for beat, and memory for news. *Communication Research*, *38(4)*, 471–496. doi:10.1177/0093650210384986

Grahn, A., Nilsson, S., Nordlund, A., Lindén, T., & Studahl, M. (2013). Cognitive impairment 3 years after neurological Varicella-zoster virus infection: A long-term case control study. *Journal of Neurology*, *260(11)*, 2761–2769. doi:10.1007/s00415-013-7057-1

Grambaite, R., Selnes, P., Reinvang, I., Aarsland, D., Hessen, E., Gjerstad, L., & Fladby, T. (2014). Executive

dysfunction in mild cognitive impairment is associated with changes in frontal and cingulate white matter tracts. *Journal of Alzheimer's Disease*, *27(2)*, 453–462. doi:10.3233/JAD-2011-110290

Gruzelier, J. H., Wilson, L., Liddiard, D., Peters, E., & Pusavat, L. (1999). Cognitive assymetri patterns in schizophrenia: Active and withdrawn syndroms and sex differences moderators. *Schizophrenia Bulletin*, *25(2)*, 349–362.

Guillem, F., Mendrek, A., Lavoie, M. E., Pampoulova, T., & Stip, E. (2009). Sex differences in memory processing in schizophrenia: An event-related potential (ERP) study. *Progress in Neuro-Psychopharmacology & Biological Psychiatry*, *33*, 1–10. doi:10.1016/j.pnpbp.2008.08.002

Guillem, F. & Mograss, M. (2005). Gender differences in memory processing: Evidence from event-related potentials to faces. *Brain and Cognition*, *57(1)*, 84–92. doi:10.1016/j.bandc.2004.08.026

Gummerum, M., Leman, P. J., & Hollins, T. S. (2013). Children's collaborative recall of shared and unshared information. *British Journal of Developmental Psychology*, *31*, 302–317. doi:10.1111/bjdp.12006

Gupta, A. & Kaur, K. (1996). Episodic memory among the aged. *Journal of the Indian Academy of Applied Psycology*, *22 (1-2)*, 107–113.

Gur, C. R., Richard, J., Chalkins, E. M., Chiavacci, R., Hansen, A. J., Bilker, B. W., . . . Gur, E. R. (2012). Age group and sex differences in performance on a computerized neurocognitive battery in children age 8-21. *Neuropsychology*, *26(2)*, 251–265. doi:doi:10.1037/a0026712.

Haan, M. N., Mungas, D. M., Gonzalez, H. M., Ortiz, T. A., Acharya, A., & Jagust, W. J. (2003). Prevalence of dementia in older latinos: The influence of type 2 diabetes mellitus, stroke and genetic factors. *Journal of the American Geriatrics Society*, *51*, 169–177.

Haász, J., Westlye, E. T., Fjær, S., Espeseth, T., Lundervold, A., & Lundervold, A. J. (2013). General fluid-type intelligence is related to indices of white matter structure in middle-aged and old adults. *NeuroImage*, *83*, 372–383. doi:10.1016/j.neuroimage.2013.06.040

Hagsand, A., Roos af Hjelmsäter, E., Granhag, P. A., Fahlke, C., & Söderpalm-Gordh, A. (2013). Bottled memories: On how alcohol affects eyewitness recall. *Scandinavian Journal of Psychology*, *54*, 188–195. doi:10.1111/sjop.12035

Hall, J. A. & Mast, M. S. (2008). Are women always more interpersonally sensitive than men? Impact of goals and content domain. *Personality and Social Psychology Bulletin*, *34*, 144–155. doi:10.1177/0146167207309192

Halpern, D. F. (1985). The influence of sex-role stereotypes on prose recall. *Sex Roles*, *12(3-4)*, 363–375. doi:10.1007/BF00287602

Halpern, J. H., Sherwood, A. R., Hudson, J. I., Gruber, S., Kozin, D., & Pope, H. G., Jr. (2011). Residual neurocognitive features of long-term ecstasy users with minimal exposure to other drugs. *Addiction*, *106 (4)*, 777–786. doi:10.1111/j.1360-0443.2010.03252.x

Han, M., Huang, X.-F., Chen, D. C., Xiu, M. H., Hui, L., Liu, H., . . . Zhang, X. Y. (2012). Gender differences in cognitive function of patients with chronic schizophrenia. *Progress in Neuro-Psychopharmacology & Biological Psychiatry*, *39(2)*, 358–363. doi:10.1016/j.pnpbp.2012.07.010

Hannay, H. J. & Rogers, J. P. (1979). Individual differences and asymmetry effects in memory for unfamiliar faces. *Cortex*, *15*, 257–267.

Harness, A., Jacot, L., Scherf, S., White, A., & Warnick, J. E. (2008). Sex differences in working memory. *Psychological Reports*, *103(1)*, 214–218. doi:10.2466/pr0.103.1.214-218

Harrington, M. G., Chiang, J., Pogoda, J. M., Gomez, M., Thomas, K., DeBoard Marion, S., . . . Fonteh, A. (2013). Executive function changes before memory in preclinical Alzheimer's pathology: A prospective, cross-sectional, case control study. *PLoS ONE*, *8(11)*, 1–12. doi:10.1371/journal.pone.0079378

Hassan, B. & Rahman, Q. (2007). Selective sexual orientation-related differences in object location memory. *Behavioral Neuroscience*, *121(3)*, 625–633. doi:10.1037/0735-7044.121.3.625

Hassmén, P., Hunt, D. P., & Dybeck, C. (2002). Effects of self-assesment on retention in rule-based learning. *Perceptual and Motor Skills*, *94(1)*, 296–306. doi:10.2466/pms.2002.94.1.296

Haut, K. M. & Barch, D. M. (2006). Sex influences on material-sensitive functional lateralization in working and episodic memory: Men and women are not all that different. *NeuroImage*, *32*, 411–422. doi:10.1016/j.neuroimage.2006.01.044

Hayden, K. M., Warren, L. H., Pieper, C. F., Ostbye, T., Tschanz, J. T., Norton, M. C., . . . Welsh-Bohmer, K. A. (2005). Identification of VaD and AD prodromes: The Cache County Study. *Alzheimer's & Dementia*, *1*, 19–29. doi:10.1016/j.jalz.2005.06.002

Hazlett, E. A., Byne, W., Brickman, A. M., Mitsis, E. M., Newmark, R., Haznedar, M. M., . . . Buchsbaum, M. S. (2010). Effects of sex and normal aging on regional brain activation during verbal memory performance. *Neurobiology of Aging*, *31*, 826–838. doi:10.1016/j.neurobiolaging.2008.10.005

Heisz, J. J., Pottruff, M. M., & Shore, D. I. (2013). Females scan more than males: A potential mechanism for sex differences in recognition memory. *Psychological Science*, *24(7)*, 1157–1163. doi:10.1177/0956797612468281

Heller, M. A., Lynn Jones, M., Walk, A. M., Schnarr, R., Hasara, A., & Litwiller, B. (2010). Sex differences in the haptic change task. *The Journal of General Psychology*, *137(1)*, 49–62.

Hellström, P., Edsbagge, M., Blomsterwall, W., Archer, T., Tisell, M., Tullberg, M., & Wikkelsø, C. (2008). Neuropsychological effects of shunt treatment in idiopathic normal pressure hydrocephalus. *Neurosurgery*, *63(3)*, 527–536. doi:10.1227/01.NEU.0000325258.16934.BB

Hellvin, T., Sundet, K., Simonsen, C., Aminoff, S. R., Lagerberg, T. V., Andreassen, O. A., & Melle, I. (2012). Neurocognitive functioning in patients recently diagnosed with bipolar disorder. *Bipolar Disorders*, *12*, 227–238. doi:10.1111/j.1399-5618.2012.01004.x

Herlitz, A., Airaksinen, E., & Nordström, E. (1999). Sex differences in episodic memory: The impact of verbal and visuospatial ability. *Neuropsychology*, *13(4)*, 590–597. doi:10.1037/0894-4105.13.4.590

Herlitz, A. & Kabir, Z. N. (2006). Sex differences in cognition among illiterate Bangladeshis: A comparison with literate Bangladeshis and Swedes. *Scandinavian Journal of Psychology*, *47*, 441–447. doi:10.1111/j.1467-9450.2006.00531.x

Herlitz, A., Nilsson, L.-G., & Bäckman, L. (1997). Gender differences in episodic memory. *Memory & Cognition*, *25(6)*, 801–811.

Herlitz, A., Reuterskiöld, L., Lovén, J., Thilers, P. P., & Rehnman, J. (2013). Cognitive sex differences are not magnified as a function of age, sex hormones, or puberty development during early adolescence. *Developmental Neuropsychology*, *38(3)*, 167–179. doi:10.1080/87565641.2012.759580

Herlitz, A. & Yonker, J. E. (2002). Sex differences in episodic memory: The influence of intelligence. *Journal of Clinical and Experimental Neuropsychology*, *24 (1)*, 107–114. doi:10.1076/jcen.24.1.107.970

Herold, C. J., Lasser, M. M., Schmid, L. A., Seidl, U., Kong, L., Fellhauer, I., . . . Schröder, J. (2013). Hippocampal volume reduction and autobiographical memory deficits in chronic schizophrenia. *Psychiatyry Research: Neuroimaging*, *211*, 189–194. doi:10.1016/j.pscychresns.2012.04.002

Herrmann, D. J., Crawford, M., & Holdsworth, M. (1992). Gender-linked differences in everyday memory performance. *British Journal of Psychology*, *83*, 221–231.

Herzmann, G., W. Bird, C., Freeman, M., & Curran, T. (2012). Effects of oxytocin on behavioral and ERP measures of recognition memory for own-race and other-race faces in women and men. *Psychoneuroendocrinology*, *38*, 2140–2151. doi:10.1016/j.psyneuen.2013.04.002

Hirata, E. S., Nakano, E. Y., Pinto Junior, J. A., Litvok, J., & Bottino, C. M. C. (2009). Prevalence and correlates of alcoholism in community-dwelling elderly living in São Paulo, Brazil. *International Journal of Geriatric Psychiatry*, *24*, 1045–1053. doi:10.1002/gps.2224

Hogervorst, E., De Jager, C., Budge, M., & Smith, A. D. (2004). Serum levels of estradiol and testosterone and performance in different cognitive domains in healthy elderly men and women. *Psychoneuroendocrinology*, *29(3)*, 405–421. doi:10.1016/S0306-4530(03)00053-2

Holsen, L. M., Dalton, K. M., Johnstone, T., & Davidson, R. J. (2008). Prefrontal social cognition network dysfunction underlying face encoding and social anxiety in fragile X syndrome. *NeuroImage*, *43*, 562–304.

doi:10.1016/j.neuroimage.2008.08.009
Honda, A. & Nihei, Y. (2009). Sex differences in object location memory: The female advantage of immediate detection of changes. *Learning and Individual Differences*, *19*, 234–237. doi:10.1016/j.lindif.2008.11.005
Horgan, T. G., Mast, M. S., Hall, J. A., & Carter, J. D. (2004). Gender differences in memory for the appearance of others. *Personality and Social Psychology Bulletin*, *30(2)*, 165–196. doi:10.1177/0146167203259928
Horgan, T. G., McGrath, M. P., & Long, J. A. (2009). The relevance of people versus objects in explaining females' advantage over males in appearance accuracy. *Sex Roles*, *60(11-12)*, 890–899. doi:10.1007/s11199-009-9596-6
Horgan, T. G., Stein, J. M., Southworth, J., & Swarbrick, M. (2012). Gender differences in memory for what others say about themselves and their family members. *Journal of Individual Diffrences*, *33(3)*, 169–174. doi:10.1027/1614-0001/a000087
Hota, N. (1983). Sex, grade and SES differences in categorization in a recall task. *Psychological Studies*, *28(1)*, 48–50.
Huang, K.-C., Lin, C.-C., & Chiang, S.-Y. (2008). Color preference and familiarity in performance on brand logo recall. *Perceptual & Motor Skills*, *107(2)*, 587–596. doi:10.2466/PMS.107.6.587-596
Hubley, A. M. (2010). Using the Rey-Osterrieth and Modified Taylor Complex Figures with older adults: A preliminary examination of accuracy score comparability. *Archives of Clinical Neuropsychology*, *25*, 197–203. doi:10.1093/arclin/acq003
Huestegge, L., Heim, S., Zettelmeyer, E., & Lange-Küttner, C. (2012). Gender-specific contribution of a visual cognition network to reading abilities. *British Journal of Psychology*, *103(1)*, 117–128. doi:10.1111/j.2044-8295.2011.02050.x
Hynd, G. W. & Obrzut, J. E. (1978). Developmental shift hypothesis and prefered memory attributes in elementary school children. *Perceptual and Motor Skills*, *46*, 167–170.
Hyttinena, L., Tuulio-Henriksson, A., Vuorioc, A. F., Kuosmanenb, N., Härkänen, T., Koskinen, S., & Strandberg, T. E. (2010). Long-term statin therapy is associated with better episodic memory in aged familial hypercholesterolemia patients in comparison with population controls. *Journal of Alzheimer's Disease*, *21*, 611–617. doi:10.3233/JAD-2010-091381
Iachini, T., Ruggiero, G., & Ruotolo, F. (2009). The effect of age on egocentric and allocentric spatial frames of reference. *Cognitive Processing*, *10(2)*, 222–224. doi:10.1007/s10339-009-0276-9
Iachini, T., Ruotolo, F., & Ruggiero, G. (2008). The effects of familiarity and gender on spatial representation. *Journal of Environmental Psychology*, *29*, 227–234. doi:10.1016/j.jenvp.2008.07.001
Iachini, T., Sergi, I., Ruggiero, G., & Gnisci, A. (2005). Gender differences in object location memory in a real three-dimensional environment. *Brain and Cognition*, *59(1)*, 52–59. doi:10.1016/j.bandc.2005.04.004
Ionescu, M. D. (2000). Sex differences in memory estimates for pictures and words. *Psychological Reports*, *87*, 315–322. doi:10.2466/pr0.2000.87.1.315
Ionescu, M. D. (2002). Sex differences in memory estimates, revisited. *Psychological Reports*, *91*, 167–172. doi:10.2466/PR0.91.5.167-172
Ionescu, M. D. (2004). Sex differences in memory estimates for pictures and words with multiple recall trials. *Psychological Reports*, *94*, 467–474. doi:10.2466/pr0.94.2.467-474
Iqbal, N., Caswell, H. L., Hare, D. J., Pilkington, O., Mercer, S., & Duncan, S. (2009). Neuropsychological profiles of patients with juvenile myoclonic epilepsy and their siblings: A preliminary controlled experimental video-EEG case series. *Epilepsy & Behavior*, *14(3)*, 516–521. doi:10.1016/j.yebeh.2008.12.025.
Iverson, G. L., Brooks, B. L., & Rennison, V. L. A. (2014). Minimal gender differences on the CNS vital signs. *Applied Neuropsychology: Adult*, *21*, 36–42. doi:10.1080/09084282.2012.721149
James, T. W. & Kimura, D. (1997). Sex differences in remembering the location of objects in an array: Location-shifts versus location-exchanges. *Evolution and Human Behavior*, *18*, 155–163.
Janowski, K., Gustaw, K., & Kasprowicz, M. (2012). Application of Choynowski's Memory Scale in assessment of patients with dementia. *Archives of Medical Science*, *8(1)*, 130–137. doi:10.5114/aoms.2012.27293

Jehna, M., Langkammer, C., Wallner-Blazek, M., Neuper, C., Loitfelder, M., Ropele, S., . . . Enzinger, C. (2011). Cognitively preserved MS patients demonstrate functional differences in processing neutral and emotional faces. *Brain Imaging and Behavior*, *5(4)*, 241–251. doi:10.1007/s11682-011-9128-1.

Jhoo, J. H., Lee, D. Y., Choo, I. H., Seo, E. H., Oh, J. S., Lee, J. S., . . . Woo, J. I. (2010). Discrimination of normal aging, MCI and AD with multimodal imaging measures on the medial temporal lobe. *Psychiatry Research: Neuroimaging*, *183*, 237–243. doi:10.1016/j.pscychresns.2010.03.006

Jiang, Y. V., Kwon, M., Shim, W. M., & Won, B.-Y. (2010). Redundancy effects in the perception and memory of visual objects. *Visual Cognition*, *18(9)*, 1233–1252. doi:10.1080/13506281003791074

Jones, M. S., Yokoi, L., Johnson, D. J., Lum, S., Cafaro, T., & Kee, D. W. (1996). Sex differences in the effectiveness of elaborative strategy use: Knowledge access comparisons. *Journal of Experimental Child Psychology*, *62*, 401–409.

Karadayi, H., Arisoy, O., Altunrende, B., Boztad, M. H., & Sercan, M. (2014). The relationship of cognitive impairment with neurological and psychiatric variables in multiple sclerosis patients. *International Journal of Psychiatry in Clinical Practice*, *18(1)*, 45–51. doi:10.3109/13651501.2013.845221

Kargopoulos, P., Bablekou, Z., Gonida, E., & Kiosseoglou, G. (2003). Effects of face and name presentation on memory for associated verbal descriptors. *American Journal of Psychology*, *116(3)*, 415–430.

Kashyap, H., Kumar, J. K., Kandavel, T., & Reddy, Y. C. J. (2013). Neuropsychological functioning in obsessive-compulsive disorder: Are executive functions the key deficit? *Comprehensive Psychiatry*, *54*, 533–540. doi:10.1016/j.comppsych.2012.12.003

Kawano, N., Awata, S., Ijuin, M., Iwamoto, K., & Ozaki, N. (2013). Necessity of normative data on the Japanese version of the Wechsler Memory Scale-Revised Logical Memory subtest for old-old people. *Geriatrics & Gerontology International*, *13(3)*, 726–730. doi:10.1111/ggi.12007

Kayser, J., Tenke, C. E., Gates, N. A., & Bruder, G. E. (2007). Reference-independent ERP old/new effects of auditory and visual word recognition memory: Joint extraction of stimulus- and response-locked neuronal generator patterns. *Psychophysiology*, *44*, 949–967. doi:10.1111/j.1469-8986.2007.00562.x

Keefe, R. S. E., Harvey, P. D., Goldberg, T. E., Gold, J. M., Walker, T. M., Kennel, C., & Hawkins, K. (2009). Norms and standardization of the brief assessment of cognition in schizophrenia (BACS). *Schizophrenia Research*, *102(1-3)*, 108–115. doi:10.1016/j.schres.2008.03.024

Keith, T. Z., Reynolds, M. R., Patel, P. G., & Ridley, K. P. (2009). Sex differences in latent cognitive abilities ages 6 to 59: Evidence from the Woodcock-Johnson III tests of cognitive abilities. *Intelligence*, *36*, 502–525. doi:10.1016/j.intell.2007.11.001

Keith, T. Z., Reynolds, M. R., Roberts, L. G., Winter, A. L., & Austin, C. A. (2011). Sex differences in latent cognitive abilities ages 5 to 17: Evidence from the Differential Ability Scales—Second Edition. *Intelligence*, *39*, 389–404. doi:10.1016/j.intell.2011.06.008

Kelley, B. J., Yeager, K. R., Pepper, T. H., & Beversdorf, D. Q. (2005). Cognitive impairment in acute cocaine withdrawal. *Cognitive and Behavioral Neurology*, *18(2)*, 108–112.

Kennet, J., McGuire, L., Willis, S. H., & Schaie, K. W. (2000). Memorability functions in verbal memory: A longitudinal approach. *Experimental Aging Research*, *26(2)*, 121–137. doi:10.1080/036107300243597

Kersker, J. L., Epley, M. L., & Wilson, J. F. (2003). Sex differences in landmark learning by children aged 5 to 12 years. *Perceptual and Motor Skills*, *96*, 329–338. doi:10.2466/pms.2003.96.1.329

Kessels, R. P. C., Nys, G. M. S., Brands, A. M. A., van den Berg, E., & Van Zandvoort, M. J. E. (2006). The modified Location Learning Test: Norms for the assessment of spatial memory function in neuropsychological patients. *Archives of Clinical Neuropsychology*, *21(8)*, 841–846. doi:10.1016/j.acn.2006.06.015

Kim, E., Lee, S. H., Lee, K. S., Cheong, H.-K., Namkoong, K., Hong, C. H., & Oh, B. H. (2012). AMPK γ2 subunit gene PRKAG2 polymorphism associated with cognitive impairment as well as diabetes in old age. *Psychoneuroendocrinology*, *37(3)*, 358–365. doi:doi:10.1016/j.psyneuen.2011.07.005

Kim, J. K. & Kang, Y. (1999). Normative study of the Korean-California Verbal Learning Test (K-CVLT). *The Clinical Neuropsychologist*, *13(3)*, 365–369.

Kim, M.-J., Kwon, J. S., & Shin, M.-S. (2013). Mediating effect of executive function on memory in normal aging adults. *Psychiatry Investiagtion*, *10*, 108–114. doi:10.4306/pi.2013.10.2.108

Kim, S. H. & Hamann, S. (2011). The effect of cognitive reappraisal on physiological reactivity and emotional memory. *International Journal of Psychophysiology*, *83*, 348–356. doi:10.1016/j.ijpsycho.2011.12.001

Kimura, D. & Clarke, P. G. (2002). Women's advantage on verbal memory is not restricted to concrete words. *Psychological Reports*, *91*, 1137–1142. doi:10.2466/pr0.2002.91.3f.1137

Kimura, D. & Seal, B. N. (2003). Sex differences in recall of real or nonsense words. *Psychological Reports*, *93*, 263–264. doi:10.2466/PR0.93.5.263-264

Kisser, J. E., Wendell, C. R., Spencer, R. J., & Waldstein, S. R. (2012). Neuropsychological performance of native versus non-native English speakers. *Archives of Clinical Neuropsychology*, *27*, 749–755. doi:10.1093/arclin/acs082

Koenig, A. K., Sakaie, K. E., Lowe, M. J., Lin, J., Stone, L., Bermel, R. A., . . . Pillips, M. D. (2013). High spatial and angular resolution diffusion-weighted imaging reveals forniceal damage related to memory impairment. *Magnetic Resonance Imaging*, *31*, 695–699. doi:10.1016/j.mri.2012.10.030

Koerts, J., Meijer, H. A., Colma, K. S. F., Tucha, L., Lange, K. W., & Tucha, O. (2013). What is measured with verbal fluency tests in Parkinson's disease patients at different stages of the disease? *Journal of Neural Transmission*, *120(3)*, 403–411. doi:10.1007/s00702-012-0885-9

Komulainen, P., Pedersen, M., Hänninen, T., Kivipelto, M., Hassinen, M., Bruunsgaard, H., . . . Rauramaa, R. (2008). BDNF is a novel marker of cognitive function in ageing women: The DR's EXTRA Study. *Neurobiology of Learning and Memory*, *90*, 596–603. doi:10.1016/j.nlm.2008.07.014

Kormi-Nouri, R., Moniri, S., & Nilsson, L.-G. (2003). Episodic and semantic memory in bilingual and monolingual children. *Scandinavian Journal of Psychology*, *44*, 47–54.

Kovac, D. & Majerova, M. (1974). Figure reproduction from the aspect of development and interfunctional relationships. *Studia Psychologica*, *16(2)*, 149–152.

Kowal, P., Chatterji, S., Naidoo, N., Biritwum, R., Fan, W., Ridaura, R. L., . . . the SAGE Collaborators. (2012). Data resource profile: The World Health Organization Study on Global AGEing and Adult Health (SAGE). *International Journal of Epidemiology*, *41*, 1639–1649. doi:10.1093/ije/dys210

Kramer, J. H., Delis, D. C., Kaplan, E., O'Donnell, L., & Prifitera, A. (1997). Developmental sex differences in verbal learning. *Neuropsychology*, *11(4)*, 577–584.

Kramer, J. H., Yaffe, K., Lengenfelder, J., & Delis, D. C. (2003). Age and gender interactions on verbal memory performance. *Journal of the International Neuropsychological Society*, *9*, 97–102. doi:10.10170S1355617703910113

Kremen, W. S., Goldstein, J. M., Seidman, L. J., Toomey, R., Lyons, M. J., Tsuang, M. T., & Faraone, S. V. (1997). Sex differences in neuropsychological function in non-psychotic relatives of schozophrenic probands. *Psychiatry Research*, *66(2-7)*, 131–144.

Kristensen, H. & Oerbeck, B. (2006). Is selective mutism associated with deficits in memory span and visual memory?: An explaoratory case-control study. *Depression and Anxiety*, *23*, 71–76. doi:10.1002/da.20140

Krohne, H. W. & Hock, M. (2008). Cognitive avoidance, positive affect, and gender as predictors of the processing of aversive information. *Journal of Research in Personality*, *42(6)*, 1572–1584. doi:10.1016/j.jrp.2008.07.015

Kromann, C. B., Jensen, M. L., & Ringsted, C. (2011). Test-enhanced learning may be a gender-related phenomenon explained by changes in cortisol level. *Medical Education*, *45*, 192–199. doi:10.1111/j.1365-2923.2010.03790.x

Kroneisen, M. & Bell, R. (2013). Sex, cheating, and disgust: Enhanced source memory for trait information that violates gender stereotypes. *Memory*, *2*, 167–181. doi:10.1080/09658211.2012.713971

Kuriyama, K., Mishima, K., Soshi, T., Honma, M., & Kim, Y. (2011). Effects of sex differences and regulation of the sleep-wake cycle on aversive memory encoding. *Neuroscience Research*, *70(1)*, 104–110.

Lachman, M. E. & Agrigoroaei, S. (2010). Low perceived control as a risk factor for episodic memory: The

mediational role of anxiety and task interference. *Memory and Cognition*, *40*, 287–296. doi:10.3758/s13421-011-0140-x

Lachman, M. E., Agrigoroaei, S., Tun, P. A., & Weaver, S. L. (2014). Monitoring cognitive functioning: Psychometric properties of the brief test of adult cognition by telephone. *Assessment*, *21(4)*, 404–417. doi:10.1177/1073191113508807

Laing, K. R., Mitchell, D., Wersching, H., Czira, M. E., Berger, K., & Baune, B. T. (2011). Brain-derived neurotrophic factor (BDNF) gene: A gender-specific role in cognitive function during normal cognitive aging of the MEMO-Study? *AGE*, *34*, 1011–1022. doi:10.1007/s11357-011-9275-8

Lajiness-O'Neill, R., Erdodi, L., & Bigler, E. D. (2011). Demographic and injury-related moderators of memory and achievement outcome in pediatric TBI. *Applied Neurposychology*, *18(4)*, 298–308. doi:10.1080/09084282.2011.595457

Lam, P. K., Kritz-Silverstein, D., Barrett-Connor, E., Milne, D., Nielsen, F., Gamst, A., . . . Wingard, D. (2008). Plasma trace elements and cognitive function in older men and women: The Rancho Bernardo study. *The Journal of Nutrition Health and Aging*, *12(1)*, 22–27.

Lanca, M. (1998). Three-dimensional representations of contour maps. *Contemporary Educational Psychology*, *23*, 22–41.

Larrabee, G. J. & Crook, T. H., III. (1993). Do men show more rapid age-associated decline in simulated everyday verbal memory than do women? *Psychology and Aging*, *8(1)*, 68–71. doi:10.1037/0882-7974.8.1.68

Larson, M., Lövdén, M., & Nilsson, L.-G. (2003). Sex differences in recollective experience for olfactory and verbal information. *Acta Psychologica*, *112*, 89–103.

Larsson, M., Öberg-Blåvarg, C., & Jönsson, F. U. (2009). Bad odors stick better than good ones. *Experimental Psychology*, *56(6)*, 375–380. doi:10.1027/1618-3169.56.6.375

Latvala, A., Casteneda, A. E., Perälä, J., Saarni, S. I., Aalto-Setälä, T., Lönnqvist, J., . . . Tuulio-Henriksson, A. (2009). Cognitive functioning in substance abuse and dependence: A popuation-based study of young adults. *Addiction*, *104*, 1558–1568. doi:10.1111/j.1360-0443.2009.02656.x

Laukka, E. J., Lövdén, M., Herliz, A., Karlsson, S., Ferencz, B., Pantzar, A., . . . Graff, C. (2013). Genetic effects on old-age cognitive functioning: A population-based study. *Psychology and Aging*, *28(1)*, 262–274. doi:10.1037/a0030829

Laureati, M., Morin-Audebrand, L., Pagliarini, G., Sulmont-Rossé, C., Köster, E. P., & Mojet, J. (2008). Food memory and its relation with age and liking: An incidental learning experiment with children, young and elderly people. *Appetite*, *51*, 273–282. doi:10.1016/j.appet.2008.02.019

Lauvsnes, M. B., Maroni, S. S., Appenzeller, S., Beyer, M. K., Greve, O. J., Kvaløy, J. T., . . . Omdal, R. (2013). Memory dysfunction in primary Sjögren's syndrome is associated with anti-NR2 antibodies. *Arthritis & Rheumatism*, *65(12)*, 3209–3217. doi:10.1002/art.38127

Lavoie, M. E., Thibault, G., Stip, E., & O'Connor, K. P. (2007). Memory and executive functions in adults with Gilles de la Tourette syndrome and chronic tic disorder. *Cognitive Neuropsychiatry*, *12(2)*, 165–181. doi:10.1080/13546800600826371

Lawrence, K., Bernstein, D., Pearson, R., Mandy, W., Campbell, R., & Skuse, D. (2008). Changing abilities in recognition of unfamiliar face photographs through childhood and adolescence: Performance on a test of non-verbal immediate memory (Warrington RMF) from 6 to 16 years. *Journal of Neuropsychology*, *2*, 27–45. doi:10.1348/174866407X231074

Lee, B. K., Glass, T. A., McAtee, M. J., Wand, G. S., Bandeen-Roche, K., Bolla, K. I., & Schwartz, B. I. (2007). Associations of salivary cortisol with cognitive function in the Baltimore memory study. *Archives of General Psychiatry*, *64(7)*, 810–818. doi:10.1001/archpsyc.64.7.810

Lee, T. M. C., Yuen, K. S. L., & Chan, C. C. H. (2002). Normative data for neuropsychological measures of fluency, attention, and memory measures for Hong Kong Chinese. *Journal of Clinical and Experimental Neuropsychology*, *24(5)*, 615–632. doi:10.1076/jcen.24.5.615.1001

Lee, T., Crawford, J. D., Henry, J. D., Trollor, J. N., Kochan, N. A., Wright, M. J., . . . Sachdev, P. S. (2012).

Mediating effects of processing speed and executive functions in age-related differences in episodic memory performance: A cross-validation study. *Neuropsychology*, *26(6)*, 776–784. doi:10.1037/a0030053

Lehmann, D. J., Refsum, H., Nurk, E., Warden, D. R., Tell, G. S., Vollset, G. E., . . . Smith, A. D. (2006). Apolipoprotein E ε4 and impaired episodic memory in community-dwelling elderly people: A marked sex difference. The Hordaland Health Study. *The Journal of Neurology, Neurosurgery, and Psychiatry*, *77*, 902–908. doi:10.1136/jnnp.2005.077818

Lehrner, J. P. (1993). Gender differences in long-term odor recognition memory: Verbal versus sensory influences and the consistency of label use. *Chemical Senses*, *18(1)*, 17–26.

Lejbak, L., Vrbancic, M., & Crossley, M. (2008). The female advantage in object location memory is robust to verbalizability and mode of presentation of test stimuli. *Brain and Cognition*, *69*, 148–153. doi:10.1016/j.bandc.2008.06.006

Lester, D. & Miller, N. H. (1974). Sex differences in inhibition on cognitive tasks. *Perceptual and Motor Skills*, *38*, 502. doi:10.2466/pms.1974.38.2.502

Levy, L. J., Astur, R. S., & Frick, K. M. (2005). Men and women differ in object memory but not performance of a virtual radial maze. *Behavioral Neuroscience*, *119(4)*, 853–862. doi:10.1037/0735-7044.119.4.853

Lewin, C. & Herlitz, A. (2002). Sex differences in face recognition—Women's faces make the difference. *Brain and Cognition*, *50*, 121–128. doi:10.1016/S0278-2626(02)00016-7

Lewin, C., Wolgers, G., & Herlitz, A. (2001). Sex differences favoring women in verbal but not in visuospatial episodic memory. *Neuropsychology*, *15(2)*, 165–173. doi:10.1037//0894-4105.15.2.165

Leynes, P. A., Crawford, J. T., Radebaugh, A. M., & Taranto, E. (2013). Event-related potential evidence of accessing gender stereotypes to aid source monitoring. *Brain Research*, *1491*, 176–187. doi:doi:10.1016/j.brainres.2012.11.013

Liben, L. S. & Signorella, M. L. (1980). Gender-related shemata and constructive memory in children. *Child Development*, *51*, 11–18.

Liben, L. S. & Signorella, M. L. (1993). Gender-schematic processing in children: The role of initial interpretations of stimuli. *Developmental Psychology*, *29*, 141–149. doi:10.1037/0012-1649.29.1.141

Lin, A., Northam, E. A., Rankins, D., Werther, G. A., & Cameron, F. J. (2010). Neuropsychological profiles of young people with type 1 diabetes 12 yr after disease onset. *Pediatric Diabetes*, *11(4)*, 235–243. doi:10.1111/j.1399-5448.2009.00588.x

Lin, K.-C., Guo, N.-W., Tsai, P.-C., Yang, C.-Y., & Guo, Y. L. (2008). Neurocognitive changes among elderly exposed to PCBs/PCDFs in Taiwan. *Environmental Health Perspectives*, *116/2*, 184–189. doi:10.1289/ehp.10134

Lindholm, T. & Christianson, S.-Å. (1998). Gender effects in eyewitness accounts of a violent crime. *Psychology, Crime & Law*, *4(4)*, 323–339. doi:10.1080/10683169808401763

Lindquist, B., Uvebrant, P., Rehn, E., & Carlsson, G. (2009). Cognitive functions in children with myelomeningocele without hydrocephalus. *Child's Nervous System*, *25(8)*, 969–975. doi:10.1007/s00381-009-0843-5

Ling, J. M., Klimaj, S., Toulouse, T., & Mayer, A. R. (2013). A prospective study of gray matter abnormalities in mild traumatic brain injury. *Neurology*, *81(21)*, 2121–2127. doi:10.1212/01.wnl.0000437302.36064.b1

Lipton, R. B., Hirsch, J., Katz, M. J., Wang, C., Sanders, A. E., Verghese, J., . . . Derby, C. A. (2010). Exceptional parental longevity associated with lower risk of Alzheimer's disease and memory decline. *Journal of the American Geriatrics Society*, *58(6)*, 1043–1049. doi:10.1111/j.1532-5415.2010.02868.x

Lobnig, B. M., Krömeke, O., Optenhostert-Porst, C., & Wolf, O. T. (2005). Hippocampal volume and cognitive performance in long-standing Type 1 diabetic patients without macrovascular complications. *Diabetic Medicine*, *23*, 32–32. doi:10.1111/j.1464-5491.2005.01716.x

Loebach Wetherell, J., Reynolds, C. A., Gatz, M., & Pedersen, N. L. (2002). Anxiety, cognitive performance, and cognitive decline in normal aging. *Psychological sciences*, *57B(3)*, 246–255.

Lombardo, M. V., Barnes, J. L., Wheelwright, S. J., & Baron-Cohen, S. (2007). Self-referential cognition and

empathy in autism. *Self-referential cognition and empathy in autism*, *2(9)*, e883.
Lorenzi-Cioldi, F. (1993). They all look alike, but so do we...sometimes: Perceptions of in-group and out-group homogeneity as a function of sex and context. *British Journal of Social Psychology*, *32*, 111–124.
Loskutova, N., Honea, R. A., Viboni, E. D., Brooks, W. M., & Burns, J. M. (2009). Bone density and brain atrophy in early Alzheimer's disease. *Journal of Alzheimer's Disease*, *18*, 777–785. doi:10.3233/JAD-2009-1185
Lövdén, M., Herlitz, A., Schellenbach, M., Grossman-Hutter, B., Krüger, A., & Lindenberger, U. (2007). Quantitative and qualitative sex differences in spatial navigation. *Cognition and Neurosciences*, *48*, 353–358. doi:10.1111/j.1467-9450.2007.00582.x
Lovén, J., Rehnman, J., Wiens, S., Lindholm, T., Peira, N., & Herlitz, A. (2012). Who are you looking at? The influence of face gender on visual attention and memory for own- and other-race faces. *Memory*, *20(4)*, 321–331. doi:10.1080/09658211.2012.658064
Lucas, J. A., Ivnik, R. J., Smith, G. E., Ferman, T. J., Willis, F. B., Petersen, R. C., & Graff-Radford, N. R. (2005). Mayo's Older African Americans Normative Studies: WMS-R norms for African American elders. *The Clinical Neuropsychologist*, *19*, 189–213. doi:10.1080/13854040590945292
Lui, S. S. Y., Wang, Y., Liu, A. C. Y., Chui, W. W. H., Gong, Q.-Y., Shum, D., . . . Chan, R. C. K. (2011). Prospective memory in patients with first-onset schizophrenia and their non-psychotic siblings. *Neuropsychologia*, *49*, 2217–2224. doi:10.1016/j.neuropsychologia.2011.04.002
Lunzer, E. A., Wilkinson, J. E., & Dolan, T. (1976). The distinctiveness of operativity as a measure of cognitive functioning in five-year-old children. *British Journal of Educational Psychology*, *46(3)*, 280–294.
Luszcz, M. A. (1992). Predictors of memory in young-old and old-old adults. *International Journal of Behavioral Development*, *15(1)*, 147–166.
Luzzi, S., Pesallaccia, M., Fabi, K., Muti, M., Viticchi, G., Provinciali, L., & Piccirilli, M. (2011). Non-verbal memory measured by Rey-Osterrieth Complex Figure B: Normative data. *Neurological Sciences*, *32(6)*, 1081–1089. doi:10.1007/s10072-011-0641-1
Lynn, R. & Wilson, G. (1993). Sex differences in cognitive abilities among Irish primary and secondary school children. *The Irish Journal of Psychology*, *14 (2)*, 293–300.
Maass, A., Kollhörster, K., Riediger, A., MacDonald, V., & Lohaus, A. (2011). Effects of violent and non-violent computer game content on memory performance in adolescents. *European Journal of Psychology of Education*, *26*, 339–353. doi:10.1007/s10212-010-0047-0
Maggi, S., Limongi, F., Noale, M., Romanato, G., Tonin, P., Rozzini, R., . . . Crepaldi, G. (2008). Diabetes as a risk factor for cognitive decline in older patients. *Dementia and Geriatric Cognitive Disorders*, *27(1)*, 24–33. doi:10.1159/000183842
Maheu, F. S., Merke, D. P., Schroth, E. A., Keil, M. F., Hardin, J., Poeth, K., . . . Ernst, M. (2008). Steroid abnormalities and the developing brain: Declarative memory for emotionally arousing and neutral material in children with congenital adrenal hyperplasia. *Psychoneuroendocrinology*, *33*, 238–245. doi:10.1016/j.psyneuen.2007.11.006
Malaspina, D., Keller, A., Antonius, D., Messinger, J. W., Goetz, D. M., Harkavy-Friedman, J., . . . Harlap, S. (2012). Olfaction and cognition in schizophrenia: Sex matters. *The Journal of Neuropsychiatry and Clinical Neurosciences*, *24(2)*, 165–175. doi:10.1176/appi.neuropsych.11070154
Malek-Ahmadi, M., Small, B. J., & Raj, A. (2011). The diagnostic value of controlled oral word association test-FAS and category fluency in single-domain amnestic mild cognitive impairment. *Dementia and Geriatric Cognitive Disorders*, *32*, 235–240. doi:10.1159/000334525
Malloy-Diniz, L. F., Lasmar, V. A. P., Gazinelli, L. d. S. R., Fuentes, D., & Salgado, J. V. (2007). The Rey Auditory-Verbal Learning Test: Applicability for the Brazilian elderly population. *Revista Brasileira de Psiquiatria*, *29(4)*, 324–329. doi:10.1590/S1516-44462006005000053
Marchant, N. L., King, S. L., Tabet, N., & Rusted, J. M. (2010). Positive effects of cholinergic stimulation favor young APOE ε4 carriers. *Neuropsychopharmacology*, *35*, 1090–1096. doi:10.1038/npp.2009.214
Marquié, J. C., Duarte, L. R., Bessières, P., Dalm, C., Gentil, C., & Ruidavets, J. B. (2010). Higher mental

stimulation at work is associated with improved cognitive functioning in both young and older workers. *Ergonomics*, *53(11)*, 1287–1301. doi:10.1080/00140139.2010.519125

Marsland, A. L., Petersen, K. L., Sathanoori, R., Muldoon, M. F., Neumann, S. A., Ryan, C., . . . Manuck, S. B. (2006). Interleukin-6 covaries inversely with cognitive performance among middle-aged community volunteers. *Psychosomatic Medicine*, *68*, 895–903. doi:10.1097/01.psy.0000238451.22174.92

Martin, K. L., Blizzard, L., Wood, A. G., Srikanth, V., Thomson, R., Sanders, L. M., & Callisaya, M. L. (2012). Cognitive function, gait, and gait variability in older people: A population-based study. *Journals of Gerontology*, *68(6)*, 726–732. doi:10.1093/gerona/gls224

Martins, I. P., Castro-Caldas, A., Townes, D. B., Ferreira, G., Rodrigues, P., Marques, S., . . . Derouen, T. (2005). Age and sex differences in neurobehavioral performance: A study of Portugese elementary school children. *International Journal of Neuroscience*, *115(12)*, 1687–1709. doi:doi:10.1080/00207450590958556

Martins, I. P., Mares, I., & Stilwell, P. A. (2012). How subjective are subjective language complaints. *European Journal of Neurology*, *19*, 666–671. doi:10.1111/j.1468-1331.2011.03635.x

Maruff, P., Collie, A., Darby, D., Weaver-Cargin, J., Masters, C., & Currie, J. (2004). Subtle memory decline over 12 months in mild cognitive impairment. *Dementia and Geriatric Cognitive Disorders*, *18 (3-4)*, 342–348. doi:10.1159/000080229

Marx, B. P., Doron-Lamarca, S., Proctor, S. P., & Vasterling, J. J. (2009). The influence of pre-deployment neurocognitive functioning on post-deployment PTSD symptom outcomes among Iraq-deployed Army soldiers. *Journal of the International Neuropsychological Society*, *15(6)*, 840–852. doi:10.1017/S1355617709990488

Maseda, A., Millán-Calenti, J. C., Lorenzo-López, L., & Núñez-Naveira, L. (2013). Efficacy of a computerized cognitive training application for older adults with and without memory impairments. *Aging Clinical and Experimental Research*, *25*, 411–419. doi:10.1007/s40520-013-0070-5

Mast, M. S. & Hall, J. A. (2006). Women's advantage at remembering others' appearance: A systematic look at the why and when of a gender difference. *Personality and Social Psychology Bulletin*, *32*, 353–364. doi:10.1177/0146167205282150

Mataix-Cols, D., Rahman, Q., Spiller, M., Alonso, P. M., Pifarreé, J., Menchón, J. M., & Velljo, J. (2006). Are there sex differences in neuropsychological functions among patients with obsessive-compulsive disorder? *Applied Neuropsychology*, *13(1)*, 42–50. doi:10.1207/s15324826an1301_6

Mathias, J. L., Dennington, V., Bowden, S. C., & Bigler, E. D. (2013). Community versus orthopaedic controls in traumatic brain injury research: How comparable are they? *Brain Injury*, *27 (7-8)*, 887–895. doi:10.3109/02699052.2013.793398

Matsuoka, K., Kotani, I., & Yamasato, M. (2012). Correct information unit analysis for determining the characteristics of narrative discourse in individuals with chronic traumatic brain injury. *Brain Injury*, *26(13-14)*, 1723–1730. doi:10.3109/02699052.2012.698789

May, R. B. & Hutt, C. (1974). Modality and sex differences in recall and recognition memory. *Child Development*, *45(1)*, 228–231.

McBurney, D. H., Gaulin, S. J. C., Devineni, T., & Adams, C. (1997). Superior spatial memory of women: Stronger evidence for the gathering hypothesis. *Evolution and Human Behavior*, *18*, 165–174.

McCall, K. M., Rellini, A. H., Seal, N., Brooke, & Meston, C. M. (2007). Sex differences in memory for sexually-relevant information. *Archives of Sexual Behavior*, *36*, 508–517. doi:10.1007/s10508-006-9106-7

McFarlane, F., Powell, M. B., & Dudgeon, P. (2002). An examination of the degree to which IQ, memory performance, socio-economic status and gender predict young children's suggestibility. *Legal and Criminological Psychology*, *7*, 227–239. doi:10.1348/135532502760274729

McGivern, R. F., Huston, J. P., Byrd, D., King, T., Siegle, G. J., & Reilly, J. (1997). Sex differences in visual recognition memory: Support for a sex-related difference in attention in adults and children. *Brain and Cognition*, *34*, 323–336.

McGivern, R. F., Mutter, K. L., Anderson, J., Wideman, G., Bodnar, M., & Huston, P. J. (1998). Gender differences in incidental learning and visual recognition memory: Support for a sex difference in unconcious environmental awareness. *Personality and Individual Differences*, *25*, 223–232.

McGugin, R. W., Richler, J. J., Herzmann, G., Speegle, M., & Gauthier, I. (2012). The Vanderbilt Expertise Test reveals domain-general and domain-specific sex effects in object recognition. *Vision Research*, *69*, 10–22. doi:10.1016/j.visres.2012.07.014

McGuinnes, D., Olsen, A., & Chapman, J. (1990). Sex differences in incidental recall for words and pictures. *Learning and Individual Differences*, *2(3)*, 263–285.

McGuinness, D. & McLaughlin, L. (1982). An investigation of sex differences in visual recognition and recall. *Journal of Mental Imagery*, *6(1)*, 203–212.

McKelvie, S. J. (1987). Sex differences, lateral reversal, and pose as factors in recognition memory for photographs of faces. *Journal of General Psychology*, *114(1)*, 13–37. doi:10.1080/00221309.1987.9711052

McKelvie, S. J., Standing, L., St. Jean, D., & Law, J. (1993). Gender differences in recognition memory for faces and cars: Evidence for the interest hypothesis. *Bulletin of the Psychonomic Society*, *31(5)*, 447–448.

McMains, S. E., Brown, G. R., Zachary, R., & Rundell, J. R. (1993). A screening test for subtle cognitive impairment early in the course of HIV infection. *Psychosomatics*, *34(5)*, 424–31. doi:10.1016/S0033-3182(93)71846-0

McWiliams, K., Goodman, G. S., Lyons, K. E., Newton, J., & Avila-Mora, E. (2014). Memory for child sexual abuse information: Simulated memory error and individual differences. *Memory & Cognition*, *42*, 151–163. doi:10.3758/s13421-013-0345-2

Mecklinger, A., Brunnemann, N., & Kipp, K. (2011). Two processes for recognition memory in children of early school age: An event-related potential study. *Journal of Cognitive Neuroscience*, *23(2)*, 435–446. doi:10.1162/jocn.2010.21455

Meekes, J., Braams, O., Braun, K. P. J., Jennekens-Schinkel, A., & van Nieuwenhuizen, O. (2013). Verbal memory after epilepsy surgery in childhood. *Epilepsy Research*, *107(1-2)*, 146–155. doi:10.1016/j.eplepsyres.2013.08.017

Mehta, U. M., Thirthalli, J., Kumar, C. N., Kumar, J. K., Keshavan, M. S., & Gangadhar, B. N. (2013). Schizophrenia patients experience substantial social cognition deficits across multiple domains in remission. *Asian Journal of Psychiatry*, *6*, 324–329. doi:10.1016/j.ajp.2013.02.001

Meier, B., Perrig-Chiello, P., & Perrig, W. (2002). Personality and memory in old age. *Aging Neuropsychology and Cognition*, *9(2)*, 135–144. doi:10.1076/anec.9.2.135.9544

Meier, S. T. (1991). Tests of the construct validity of occupational stress measures with college students: Failure to support discriminant validity. *Journal of Counseling Psychology*, *38(1)*, 91–97.

Meijer, J., Simons, C. J. P., Quee, P. J., & Verweij, K. (2011). Cognitive alterations in patients with non-affective psychotic disorder and their unaffected siblings and parents. *Acta Psychiatrica Scandinavica*, *125*, 66–76. doi:10.1111/j.1600-0447.2011.01777.x

Meléndes-Moral, J. C., Tomas, J. M., Blasco-Bataller, S., Olivier, A., & Navarro, E. (2010). Comparison between Spanish young and elderly people evaluated using Rivermead Behavioural Memory Test. *Aging, Neuropsychology and Cognition*, *17*, 545–555. doi:10.1080/13825581003763039

Mellet, E., Jobard, G., Zago, L., Crivello, F., Petit, L., Joliot, M., . . . Tzourio-Mazoyer, N. (2014). Relationships between hand laterality and verbal and spatial skills in 436 healthy adults balanced for handedness. *Laterality: Asymmetries of Body, Brain and Cognition*, *19(4)*, 383–404. doi:10.1080/1357650X.2013.796965

Merema, M. R., Speelman, C. P., Foster, J. K., & Kaczmarek, E. A. (2013). Neuroticism (not depressive symptoms) predicts memory complaints in some community-dwelling older adults. *The American Journal of Geriatric Psychiatry*, *21(8)*, 729–736. doi:10.1016/j.jagp.2013.01.059

Meyers-Levy, J. & Maheswaran, D. (1991). Exploring differences in males' and females' processing strategies.

Journal of Consumer Research, *18(1)*, 63–70.

Mielke, M. M., Wiste, H. J., Weigand, S. D., Knopman, D. S., Lowe, V. J., Roberts, R. O., . . . Jack, C. R., Jr. (2012). Indicators of amyloid burden in a population-based study of cognitively normal elderly. *Neurology*, *79(15)*, 1570–1577. doi:10.1212/WNL.0b013e31826e2696

Miller, L. K. & Santoni, V. (1986). Sex differences in spatial abilities: Strategic and experiential correlates. *Acta Psychologica*, *62(3)*, 225–235. doi:10.1016/0001-6918(86)90089-2

Minett, T. S. C., Dean, J. L., Firbank, M., English, P., & O'Brien, J. T. (2005). Subjective memory complaints, white-matter lesions, depressive symptoms, and cognition in elderly patients. *The American Journal of Geriatric Psychiatry*, *13(8)*, 665–671. doi:10.1097/00019442-200508000-00005

Mizuno, K., Tanaka, M., Fukuda, S., Yamano, E., Shigihara, Y., Imai-Matsumura, K., & Watanbe, Y. (2011). Low visual information-processing speed and attention are predictors of fatigue in elementary and junior high school students. *Bahvioral and Brain Functions*, *7(20)*, 1–7. doi:10.1186/1744-9081-7-20

Moffat, S. D., Hampson, E., & Hatzipantelis, M. (1998). Navigation in a "virtual" maze: Sex differences and correlation with psychometric measures of spatial ability in humans. *Evolution and Human Behavior*, *19(2)*, 73–87. doi:10.1016/S1090-5138(97)00104-9

Mokri, H., Ávila-Funes, J. A., Meillon, C., Gutiérres Robledo, L. M., & Amieva, H. (2013). Normative data for the Mini-Mental State Examination, the Free and Cued Selective Reminding Test and the Isaacs Set Test for an older adult Mexican population: The Coyoacán Cohort Study. *The Clinical Neuropsychologist*, *27(6)*, 1004–1018. doi:10.1080/13854046.2013.809793

Morales, M., Campo, P., Fernández, A., Moreno, D., Yáñez, J., & Sañudo, I. (2010). Normative data for a six-trial administration of a Spanish version of the Verbal Selective Reminding Test. *Archives of Clinical Neuropsychology*, *25(8)*, 745–761. doi:10.1093/arclin/acq076

Morgan, C. J. A., Perry, E. B., Cho, H.-S., Krystal, J. H., & D'Souza, D. C. (2006). Greater vulnerability to the amnestic effects of ketamine in males. *Psychopharmacology*, *187(4)*, 405–414. doi:10.1007/s00213-006-0409-0

Morrens, M., Hulstijn, W., & Sabbe, B. (2008). The effects of atypical and conventional antipsychotics on reduced processing speed and psychomotor slowing in schizophrenia: A cross-sectional exploratory study. *Clinical Therapeutics*, *30(4)*, 684–692. doi:10.1015/j.clinthera.2008.04.012

Mueller, S. C., Jackson, C. P. T., & Skelton, R. W. (2008). Sex differences in a virtual water maze: An eye tracking and pupillometry study. *Behavioral Brain Research*, *193*, 209–215. doi:10.1016/j.bbr.2008.05.017

Müller, B. W., Gimbel, K., Keller-Pliessnig, G., A. Sartory, Gastpar, M., & Davids, E. (2007). Neuropsychological assessment of adult patients with attention-deficit/hyperactivity disorder. *European Archives of Psychiatry and Clinical Neuroscience*, *257*, 112–119. doi:10.1007/s00406-006-0688-9

Munro, C. A., Winicki, J. M., Schretlen, D. J., Gower, E. W., Turano, K. A., Muñoz, B., . . . West, S. K. (2012). Sex differences in cognition in healthy elderly individuals. *Aging, Neuropsychology, and Cognition*, *19 (6)*, 759–768. doi:10.1080/13825585.2012.690366

Murre, J. M. J., Janssen, S. M. J., Romke, R., & Meeter, M. (2013). The rise and fall of immediate and delayed memory for verbal and visuospatial information from late childhood to late adulthood. *Acta Psychologica*, *142*, 96–107. doi:10.1016/j.actpsy.2012.10.005

Nairne, J. S., Pandeirada, J. N. S., Gregory, K. J., & Van Arsdall, J. E. (2009). Adaptive memory: Fitness relevance and the hunter-gatherer mind. *Psychological Science*, *20*, 740–746. doi:10.1111/j.1467-9280.2009.02356.x

Närhi, V., Lehto-Salo, P., & Marttunen, M. (2010). Neuropsychological subgroups of adolescents with conduct disorder. *Scandinavian Journal of Psychology*, *51(3)*, 278–284. doi:10.1111/j.1467-9450.2009.00767.x

Naveh-Benjamin, M., Maddox, G. B., Jones, P., Old, S., & Kilb, A. (2011). The effects of emotional arousal and gender on the associative memory deficit of older adults. *Memory & cognition*, *40(4)*, 551–566. doi:10.3758/s13421-011-0169-x

Newhouse, P., Newhouse, C., & Astur, R. S. (2007). Sex differences in visual-spatial learning using a virtual water

maze in pre-pubertal children. *Behavioural Brain Research*, *183*, 1–7. doi:10.1016/j.bbr.2007.05.011

Ngandu, T., Helkala, E.-L., Soininen, H., Winblad, B., Tuomilehto, J., Nissinen, A., & Kivipelto, M. (2006). Alcohol drinking and cognitive function: Findings from the Cardiovascular Risk Factors Aging and Dementia (CAIDE) Study. *Dementia and Geriatric Cognitive Disorders*, *23(3)*, 140–149. doi:10.1159/000097995

Nieto, A., Correia, R., de Nóbrega, E., Montón, F., Hess, S., & Barroso, J. (2012). Cognition in Friedreich ataxia. *Cerebellum*, *11*, 834–844. doi:10.1007/s12311-012-0363-9

Nobili, F., Mazzei, D., Dessi, B., Morbelli, S., Brugnolo, A., Barbieri, P., . . . Pagani, M. (2010). Unawareness of memory deficit in amnestic MCI: FDG-PET findings. *Journal of Alzheimer's Disease*, *22*, 993–1003. doi:10.3233/JAD-2010-100423

Novotny, J. A., Rumpler, W. V., Riddick, H., Herbert, J. R., Rhodes, D., Judd, J. T., . . . Briefel, R. (2003). Personality characteristics as predictors of underreporting of energy intake on 24-hour dietary recall interviews. *Journal of the American Dietic Association*, *103(9)*, 1146–1151. doi:10.1016/S0002-8223(03)00975-1

Öberg, C., Larsson, M., & Bäckman, L. (2002). Differential sex effects in olfactory functioning: The role of verbal processing. *Journal of the International Neuropsychological Society*, *8(5)*, 691–698. doi:10.1017/S1355617702801424

O'Hara, R., Miller, E., Liao, C.-P., Way, N., Lin, X., & Hallmayer, J. (2006). COMT genotype, gender and cognition in community-dwelling, older adults. *Neuroscience Letters*, *409*, 205–209. doi:10.1016/j.neulet.2006.09.047

Ojeda, N., Sánchez, P., Peña, J., Elizagárate, E., Yoller, A. B., Larumbe, J., . . . Ezcurra, J. (2010). Verbal fluency in schizophrenia: Does cognitive performance reflect the same underlying mechanisms in patients and healthy controls? *Journal of Nervous and Mental Disease*, *198(4)*, 286–291. doi:10.1097/NMD.0b013e3181d61748

Østby, Y., Tamnes, C. K., Fjell, A. M., & Walhovd, K. B. (2012). Dissociating memory processes in the developing brain: The role of hippocampal volume and cortical thickness in recall after minutes versus days. *Cerebral Cortex*, *22*, 381–390. doi:10.1093/cercor/bhr116

O'Sullivan, J. T. (1997). Effort, interest, and recall: Beliefs and behaviors of preschoolers. *Journal of Experimental Child Psychology*, *65(1)*, 43–67. doi:10.1006/jecp.1996.2355

Otero Dadin, C., Rodriquez Salgado, D., & Andrade Fernández, E. (2009). Natural sex hormone cycles and gender differences in memory. *Actas Espanolas de Psiquiatria*, *37(2)*, 68–74. Ott, D. A. & Lyman, R. D. (1993). Automatic and effortful memory in children exhibiting attention-deficit hyperactivity disorder. *Journal of Clinical Child Psychology*, *22(4)*, 420–427.

Owen, K. & Lynn, R. (1993). Sex differences in primary cognitive abilities among blacks, Indians and whites in South Africa. *Journal of Biosocial Science*, *25*, 557–560.

Palmer, M. A., Brewer, N., & Horry, R. (2013). Understanding gender bias in face recognition: Effects of divided attention at encoding. *Acta Psychologica*, *142*, 362–369. doi:10.1016/j.actpsy.2013.01.009

Palomo, R., Casals-Coll, M., Sánchez-Benavides, G., Quintana, M., Manero, R. M., Rognoni, T., . . . Pena-Casanova, J. (2013). Spanish normative studies in young adults (NEURONORMA young adults project): Norms for the Rey-Osterrieth Complex Figure (copy and memory) and Free and Cued Selective Reminding Test. *Neurologia*, *28(4)*, 226–235. doi:10.1016/j.nrl.2012.03.008

Parada, M., Corral, M., Caamano-Isorna, F., Mota, N., Crego, A., Holguin, S. R., & Cadaveira, F. (2011). Binge drinking and declarative memory in university students. *Alcoholism: Clinical and Experimental Research*, *35(8)*, 1475–1484. doi:10.1111/j.1530-0277.2011.01484.x

Parks, C. M., Iosif, A.-M., Farias, S., Reed, B., Mungas, D., & DeCarli, C. (2011). Executive function mediates effects of white matter hyperintensities on episodic memory. *Neuropsychologia*, *49(10)*, 2817–2824. doi:10.1016/j.neuropsychologia.2011.06.003

Passamonti, L., Novellino, F., Cerasa, A., Chiriaco, C., Rocca, F., Matina, M. S., . . . Quattrone, A. (2011). Altered cortical-cerebellar circuits during verbal working memory in essential tremor. *Brain*, *134(Pt 8)*, 2274–2286. doi:10.1093/brain/awr164

Pati, P. & Dash, A. S. (1990). Effects of grade, sex and achievement levels on intelligence, incidental memory and and Stroop scores. *Psychological Studies*, *35(1)*, 36–40.

Pauli, P., Dengler, W., & Wiedemann, G. (2005). Implicit and explicit memory processes in panic patients as reflected in behavioral and electrophysiological measures. *Journal of Behavior Therapy and Experimental Psychiatry*, *36(2)*, 111–127. doi:10.1016/j.jbtep.2004.08.003

Paulo, A. C., Sampaio, A., Santos, N. C., Costa, P. S., Cunha, P., Zihl, J., . . . Sousa, N. (2011). Patterns of cognitive performance in healthy ageing in northern Portugal: A cross-sectional analysis. *PLoS ONE*, *6(9)*, 1–9. doi:10.1371/journal.pone.0024553

Pauls, F., Petermann, F., & Lepach, A. C. (2013). Gender differences in episodic memory and visual working memory including the effects of age. *Memory*, *21(7)*, 857–874. doi:10.1080/09658211.2013.765892

Pavlik, V., Massman, P., Barber, R., & Doody, R. (2013). Differences in the association of peripheral insulin and cognitive function in non-diabetic Alzheimer's disease cases and normal controls. *Journal of Alzheimer's Disease*, *34*, 449–456. doi:10.3233/JAD-121999

Payne, J., Jackson, E., Ryan, L., Hoscheidt, S., Javobs, J., & Nadel, L. (2006). The impact of stress on neutral and emotional aspects of episodic memory. *Memory*, *14(1)*, 1–16. doi:10.1080/09658210500139176

Peavy, G. M., Santiago, P. D., & Edland, S. D. (2012). Subjective memory complaints are associated with diurnal measures of salivary cortisol in cognitively intact older adults. *The American Journal of Geriatric Psychiatry*, *21(9)*, 925–928. doi:10.1016/j.jagp.2013.01.022

Pedersen, M., Pedersen, K. K., Bruunsgaard, H., Krabbe, K. S., Thomsen, C., Faerch, K., . . . Mortensen, E. L. (2012). Cognitive functions in middle aged individuals are related to metabolic disturbances and aerobic capacity: A cross-sectional study. *PLoS ONE*, *7(12)*, 1–7. doi:10.1371/journal.pone.0051132

Pena-Casanova, J., Blesa, R., Aguilar, M., Gramunt-Fombuena, N., Gómez-Ansón, B., Molinuevo, J. L., . . . Sol, J. M. (2009). Spanish Multicenter Normative Studies (NEURONORMA Project): Methods and sample characteristics. *Archives of Clinical Neuropsychology*, *24*, 307–319. doi:10.1093/arclin/acp027

Pérez-Carpinell, J., Camps, V. J., & Trottini, M. (2008). Color memory in children. *COLOR research and application*, *33(5)*, 872–380. doi:10.1002/col.20433

Pérez-Carpinell, J., Camps, V. J., Trottini, M., & Perez-Baylach, C. M. (2006). Color memory in elderly adults. *COLOR research and application*, *31*, 458–467. doi:10.1002/col.20258

Perrig-Chiello, P., Perrig, W. J., & Stähelin, H. B. (2000). Differential aspects of memory self-evaluation in old and very old people. *Aging & Mental Health*, *4(2)*, 130–135. doi:10.1080/13607860050008646

Persinger, M. A. & Richards, P. M. (1995). Women reconstruct more details than men for a complex five-minute narrative: Implications for right-hemispheric factors in the serial memory effect. *Perceptual and Motor Skills*, *80*, 403–410.

Phillips, L. H., Henry, J. D., Scott, C., Summers, F., Whyte, M., & Cook, M. (2011). Specific impairments of emotion perception in multiple sclerosis. *Neuropsychology*, *25(1)*, 131–136. doi:10.1037/a0020752

Phillips, M. A., Childs, C. E., Calder, P. C., & Rogers, P. J. (2010). Lower omega-3 fatty acid intake and status are associated with poorer cognitive function in older age: A comparison of individuals with and without cognitive impairment and Alzheimer's disease. *Nutritional Neuroscience*, *15(6)*, 271–277. doi:10.1179/1476830512Y.0000000026

Phillips, S. & Fox, P. (1998). An investigation into the effects of nicotine gum on short-term memory. *Psychopharmacology*, *140(4)*, 429–433.

Pickel, K. L. (2009). The weapon focus effect on memory for female versus male perpetrators. *Memory*, *17(6)*, 664–678. doi:10.1080/09658210903029412

Piper, B. J., Acevedo, S. F., Edwards, K. R., Curtiss, A. B., McGinnis, G. J., & Raber, J. (2011). Age, sex and handedness differentially contribute to neurospatial function on the Memory Island and Novel-Image Novel-Location tests. *Physiology & Behavior*, *103(5)*, 513–522. doi:10.1016/j.physbeh.2011.03.024

Pontón, M. O., Satz, P., Herrera, L., Ortiz, F., Urrutia, C. P., Young, R., . . . Namerow, N. (1996). Normative data stratified by age and education for the Neuropsychological Screening Battery for Hispanics (NeSBHIS):

Initial report. *Journal of the International Neuropsychological Society*, *2(2)*, 96–104.
Portin, R., Saarijärvi, S., Joukamaa, M., & Salokangas, R. K. R. (1995). Education, gender and cognitive performance in a 62-year-old normal population: Results from the Turva Project. *Psychological Medicine*, *25*, 1295–1298.
Postma, A., Izendooorn, R., & De Haan, E. H. F. (1998). Sex differences in object location memory. *Brain and Cognition*, *36(3)*, 334–345.
Postma, A., Jager, G., Kessels, R. P. C., Koppeschaar, H. P. F., & van Honk, J. (2003). Sex differences for selective forms of spatial memory. *Brain and Cognition*, *54*, 24–34. doi:10.1016/S0278-2626(03)00238-0
Postma, A., Winkel, J., Tuiten, A., & van Honk, J. (1999). Sex differences and menstrual cycle effects in human spatial memory. *Psychoneuroendocrinology*, *24(2)*, 175–192.
Potts, R., Morse, M., Felleman, E., & Masters, J. C. (1986). Children's emotions and memory for affective narrative content. *Motivation and Emotion*, *10(1)*, 39–57.
Pouliot, S. & Gagnon, S. (2005). Is egocentric space automatically encoded? *Acta Psychologica*, *118*, 193–210. doi:doi:10.1016/j.actpsy.2004.10.016
Rabitt, P., Donlan, C., Watson, P., McInnes, L., & Bent, N. (1995). Unique and interactive effects of depression, age, socioeconomic advantage, and gender on cognitive perfromance of normal healthy older people. *Psychology and Aging*, *3*, 307–313.
Ragland, J. D., Coleman, A. R., Gur, C., Ruben, Glahn, D. C., & Gur, R. E. (2000). Sex differences in brain-behavior relationships between verbal episodic memory and resting regional cerebral blood flow. *Neuropsychologia*, *38(4)*, 451–461.
Rahman, Q., Abrahams, S., & Jussab, F. (2005). Sex differences in a human analogue of the Radial Arm Maze: The "17-Box Maze Test". *Brain and Cognition*, *58*, 312–317. doi:10.1016/j.bandc.2005.03.001
Rahman, Q., Bakare, M., & Serinsu, C. (2011). No sex differences in spatial location memory for abstract designs. *Brain and Cognition*, *76(1)*, 15–19. doi:10.1016/j.bandc.2011.03.012
Rahman, Q. & Clarke, C. D. (2005). Sex differences in neurocognitive functioning among abstinent recreational cocaine users. *Psychopharmacology*, *181*, 374–380. doi:10.1007/s00213-005-2257-8
Rahman, Q., Newland, C., & Smyth, B. M. (2011). Sexual orientation and spatial position effects on selective forms of object location memory. *Brain and Cognition*, *75*, 217–224. doi:10.1016/j.bandc.2010.11.010
Rahman, Q., Wilson, G. D., & Abrahams, S. (2003). Sexual orientation related differences in spatial memory. *Journal of the International Neuropsychological Society*, *9*, 376–383. doi:10.10170S1355617703930037
Rao, N. & Moely, B. E. (1989). Producing memory strategy maintenance and generalization by explicit or implicit training of memory knowledge. *Journal of Experimental Child Psychology*, *48(3)*, 335–352.
Rapeli, P., Fabritius, C., Alho, H., M., S., Wahlbeck, K., & Kalska, H. (2007). Methadone vs. buprenorphine/naloxone during early opioid substitution treatment: A naturalistic comparison of cognitive performance relative to healthy controls. *BMC Clinical Pharmacology*, *7*, 1–10. doi:10.1186/1472-6904-7-5
Rapisarda, A., Lim, T. F., Lim, M., Collinson, S. L., Kraus, M. S., & Keefe, R. S. E. (2013). Applicability of the MATRICS Consensus Cognitive Battery in Singapore. *The Clinical Neuropsychologist*, *27(3)*, 455–469. doi:10.1080/13854046.2012.762120
Raz, N., Rodrigue, K. M., Kennedy, K. M., & Land, S. (2009). Genetic and vascular modifiers of age-sensitive cognitive skills: Effects of COMT, BDNF, ApoE and hypertension. *Neuropsychology*, *23(1)*, 105–116. doi:10.1037/a0013487
Razumnikova, O. M. & Vol'f, N. V. (2007). Gender differences in interhemisphere interactions during distributed and directed attention. *Neuroscience and Behavioral Physiology*, *37(5)*, 429–434. doi:10.1007/s11055-007-0031-6
Read, S., Pedersen, N. L., Gatz, M., Berg, S., Vuoksimaa, E., Malmberg, B., . . . McClearn, G. E. (2006). Sex differences after all those years? Heritability of cognitive abilities in old age. *The Journals of Gerontology, Series B: Psychological Sciences and Social Sciences*, *61B(3)*, 137–143.

Rehmman, J. & Herlitz, A. (2006). Higher face recognition ability in girls: Magnified by own-sex and own-ethnicity bias. *Memory*, *14(3)*, 289–296. doi:10.1080/09658210500233581

Rehnman, J. & Herlitz, A. (2007). Women remember more faces than men do. *Acta Psychologica*, *124*, 344–355. doi:10.1016/j.actpsy.2006.04.004

Reijmer, Y. D., Brundel, M., de Bresser, J., Kappelle, L. J., Leemans, A., & Biessels, G. J. (2013). Microstructural white matter abnormalities and cognitive functioning in type 2 diabetes: A diffusion tensor imaging study. *Diabetes Care*, *36(1)*, 137–144. doi:10.2337/dc12-0493

Reis, J. P., Launer, L. J., Terry, J. G., Loria, C. M., Al Hazzouri, A. Z., Sidney, S., . . . Carr, J. J. (2013). Subclinical atherosclerotic calcification and cognitive functioning in middle-aged adults: The CARDIA study. *Atherosclerosis*, *231(1)*, 72–77. doi:10.1016/j.atherosclerosis.2013.08.038

Reiswich, J., Krumova, E. K., David, M., Stude, P., Tegenthoff, M., & Maier, C. (2012). Intact 2D-form recognition despite impaired spatial acuity in complex regional pain syndrome type I. *Pain*, *153(7)*, 1484–1494. doi:10.1016/j.pain.2012.04.005

Resmini, E., Santos, A., Gómez-Anson, B., Vives, Y., Pires, P., Crespo, I., . . . Webb, S. M. (2012). Verbal and visual memory performance and hippocampal volumes, measured by 3-Tesla magnetic resonance imaging, in patients with Cushing’s syndrome. *The Journal of Clinical Endocrinology & Metabolism*, *97(2)*, 663–671. doi:10.1210/jc.2011-2231

Ridout, N., Dritschel, B., Matthews, K., McVicar, M., Reid, I. C., & O’Carroll, R. E. (2009). Memory for emotional faces in major depression following judgement of physical facial characteristics at encoding. *Cognition and Emotion*, *23(4)*, 739–752. doi:10.1080/02699930802121137

Rizk-Jackson, A. M., Acevedo, S. F., Inman, D., Howieson, D., Benice, T. S., & Raber, J. (2006). Effects of sex on object recognition and spatial navigation in humans. *Behavioural Brain Research*, *173*, 181–190. doi:10.1016/j.bbr.2006.06.029

Robinson, N. M., Abott, R. D., Berninger, V. W., & Busse, J. (1996). The structure of abilities in math-precocious young children: Gender similarities and differences. *Journal of Educational Psychology*, *88(2)*, 341–352.

Romano, R., Bertolino, A., Gigante, A., Martino, D., Livrea, P., & Defazio, G. (2014). Impaired cognitive functions in adult-onset primary cranial cervical dystonia. *Parkinsonism and Related Disorders*, *20(2)*, 162–165. doi:10.1016/j.parkreldis.2013.10.008

Rosenbloom, M. J., O’Reilly, A., Sassoon, S. A., Sullivan, E. V., & Pfefferbaum, A. (2005). Persistent cognitive deficits in community-treated alcoholic men and women volunteering for research: Limited contribution from psychiatric comorbidity. *Journal of Studies of Alcohol*, *66(2)*, 254–265.

Rothen, N. & Meier, B. (2009). Do synesthetes have a general advantage in visual search and episodic memory? A case for group studies. *PLoS ONE*, *4(4)*, 1–9. doi:10.1371/journal.pone.0005037

Rucklidge, J. J. (2006). Gender differences in neuropsychological functioning of New Zealand adolescents with and without Attention Deficit Hyperactivity Disorder. *International Journal of Disability, Developement and Education*, *53(1)*, 47–66. doi:10.1080/10349120600577402

Ruff, R. M., Light, R. H., & Quayhagen, M. (1988). Selective Reminding Tests: A normative study of verbal learning in adults. *Journal of Clinical and Experimental Neuropsychology*, *11(4)*, 539–550.

Ruffieux, N., Njamnshi, A. K., Mayer, E., Sztajzel, R., Eta, S. C., Doh, R. F., . . . Hauert, C.-A. (2010). Neuropsychology in Cameroon: First normative data for cognitive tests among school-aged children. *Child Neuropsychology*, *16(1)*, 1–19. doi:10.1080/09297040902802932

Ruggiero, G., Sergi, I., & Iachini, T. (2008). Gender differences in remembering and inferring spatial distances. *Memory*, *16(8)*, 821–835. doi:10.1080/09658210802307695

Ruiz de Azua, S., Matute, C., Stertz, L., Mosquera, F., Aitor, P., de la Rosa, I., . . . Gonzales-Pinto, A. (2013). Plasma brain-derived neurotrophic factor levels, learning capacity and cognition in patients with first episode psychosis. *BMC Psychiatry*, *13*, 1–9. doi:10.1186/1471-244X-13-27

Rupp, C. I., Fleischhacker, W. W., Hausmann, A., Hinterhuber, H., & Kurz, M. (2006). Executive function and memory in relation to olfactory deficits in alcohol-dependent patients. *Alcoholism: Clincal and*

Experimental Research, *30(8)*, 1355–1362. doi:10.1111/j.1530-0277.2006.00162.x

Sabia, S., Nabi, H., Kivimaki, M., Shipley, M. J., Marmot, M. G., & Singh-Manoux, A. (2009). Health behaviors from early to late midlife as predictors of cognitive function the Whitehall II study. *American Journal of Epidemiology*, *170(4)*, 428–437. doi:10.1093/aje/kwp161

Said, J. A., Shores, A., Batchelor, J., & Thomas, P., D. Fahey. (1990). The Children's Auditory-Verbal Selective Reminding Test: Equivalence and test-retest reliability of two forms with boys and girls. *Developmental Neuropsychological*, *6(3)*, 225–230.

Salthouse, T. A. & Siedlecki, K. L. (2007). An individual difference analysis of false recognition. *American Journal of Psychology*, *120(3)*, 429–458.

Santos, F. H., Mello, C. B., Bueno, O. F. A., & Dellatolas, G. (2005). Cross-cultural differences for three visual memory tasks in Brazilian children. *Perceptual and Motor Skills*, *101(2)*, 421–433. doi:10.2466/pms.101.2.421-433

Santos, N. D., Costa, P. S., Cunha, P., Cotter, J., Sampaio, A., Zihl, J., . . . Sousa, N. (2013). Mood is a key determinant of cognitive performance in community-dwelling older adults: A cross-sectional analysis. *Age*, *35(5)*, 1983–1993. doi:10.1007/s11357-012-9482-y

Savage, R. M. & Gouvier, W. D. (1992). Rey Auditory-Verbal Learning Test: The effect of age and gender, and norms for delayed recall and story recognition recall. *Archives of Clinical Neuropsychology*, *7*, 407–414.

Savaskan, E., Ehrhardt, R., Schulz, A., Walter, M., & Schachinger, H. (2007). Post-learning intranasal oxytocin modulates human memory for facial identity. *Psychoneuroendocrinology*, *33(3)*, 368–374. doi:10.1016/j.psyneuen.2007.12.004

Scanlon Jones, S. (1984). Adult recipients of infant communtications: A sex difference in the salience of early "social" smiling. *Infant Behavior and Development*, *7*, 211–221.

Schaefer, C., von Rhein, M., Knirsch, W., Huber, R., Natalucci, G., Caflisch, J., . . . Latal, B. (2013). Neurodevelopmental outcome, psychological adjustment, and quality of life in adolescents with congenital heart disease. *Developmental Medicine & Child Neurology*, *55(12)*, 1143–1149. doi:10.1111/dmcn.12242

Schatz, P., Moser, R. S., Solomon, G. S., Ott, S. D., & Karpf, R. (2012). Prevalence of invalid computerized baseline neurocognitive test results in high school and collegiate athletes. *Journal of Athletic Training*, *47(3)*, 289–296. doi:10.4085/1062-6050-47.3.14

Schirmer, A., Chen, C.-B., Ching, A., Tan, L., & Hong, R. Y. (2013). Vocal emotions influence verbal memory: Neural correlates and interindividual differences. *Cognitive, Affective & Behavioral Neuroscience*, *13(1)*, 80–93. doi:10.3758/s13415-012-0132-8

Schmitzer-Torbert, N. (2007). Place and response learning in human virtual navigation: Behavioral measures and gender differences. *Behavioral Neuroscience*, *121(2)*, 277–290. doi:10.1037/0735-7044.121.2.277

Schofield, P. W., Ebrahimi, H., Jones, A. L., Bateman, G. A., & Murray, S. R. (2012). An olfactory 'stress test' may detect preclinical Alzheimer's disease. *BMC Neurology*, *12(24)*, 1–8. doi:10.1186/1471-2377-12-24

Schretlen, D. J., Inscore, A. B., Jinnah, H. A., Rao, V., Gordon, B., & Pearlson, G. D. (2007). Serum uric acid and cognitive function in community-dwelling older adults. *Neuropsychology*, *21*, 136–140. doi:10.1037/0894-4105.21.1.136

Schwartz, N. H. & Philippe, A. E. (1991). Individual differences in the retention of maps. *Contemporary Educational Psychology*, *16*, 171–182.

Seasmon, J. G., Guerry, J. D., Marsh, G. P., & Tracy, M. C. (2002). Accurate and false recalls in the Deese/Roediger and McDermott procedure: A methodological note on sex of participant. *Psychological Reports*, *91*, 423–428. doi:10.2466/pr0.2002.91.2.423

Segalàs, C., Alonso, P., Labad, J., Real, E., Pertusa, A., Jaurrieta, N., . . . Vellejo, J. (2010). A case-control study of sex differences in strategic processing and episodic memory in obsessive-compulsive disorder. *Comprehensive Psychiatry*, *51*, 303–311. doi:10.1016/j.comppsych.2009.05.008

Segura, B., Jurado, M. A., Freixenet, N., Albuin, J., C. Muniesa, & Junqué, C. (2009). Mental slowness and

executive dysfunctions in patients with metabolic syndrome. *Neuroscience Letters*, *462/1*, 49–53. doi:10.1016/j.neulet.2009.06.071

Serra, L., Bozzali, M., Cercigiani, M., Perri, R., Fadda, L., Caltagirone, C., & Carlesimo, G. A. (2010). Recollection and familiarity in Amnesic Mild Cognitive Impairment. *Neuropsychology*, *25(3)*, 316–326. doi:10.1037/a0017654

Sharps, M. J., Welton, A. L., & Price, J. L. (1993). Gender and task in the determination of spatial cognitive performance. *Psychology of Women Quarterly*, *17*, 71–83.

Shi, J., Tian, J., Wei, M., Miao, Y., & Wang, Y. (2012). The utility of the Hopkins Verbal Learning Test (Chinese version) for screening dementia and mild cognitive impairment in a Chinese population. *BMC Neurology*, *12(136)*, 1–8. doi:10.1186/1471-2377-12-136

Shichita, K., Hatano, S., Ohashi, Y., Shibata, H., & Matuzaki, T. (1986). Memory changes in the Benton Visual Retention Test between ages 70 and 75. *Journal of Gerontology*, *41(3)*, 385–386.

Silverman, I., Choi, J., & Peters, M. (2007). The hunter-gatherer theory of sex differences in spatial abilities: Data from 40 countries. Archives of Sexual Behavior, 36, 261–268. doi:10.1007/s10508-006-9168-6

Simons, J. S., Dodson, C. S., Bell, D., & Schacter, D. L. (2004). Specific- and partial-source memory: Effects of aging. *Psychology and Aging*, *19(4)*, 689–694. doi:10.1037/0882-7974.19.4.689

Simpson, E. E. A., Maylor, E. A., Rae, G., Meunier, N., Andriollo-Sanchez, M., Catasta, G., . . . Coudray, C. (2005). Cognitive function in healthy older European adults: The ZENITH study. *European Journal of Clinical Nutrition*, *59(2)*, 26–30. doi:10.1038/sj.ejcn.1602294

Skjerve, A., Nordhus, I. H., Engedal, K., Pallesen, S.., Brækhus, A., & Nygaard, H. A. (2007). Seven minute screen performance in a normal elderly sample. *International Journal of Geriatric Psychiatry*, *22*, 764–769. doi:10.1002/gps.1736

Slegers, K., van Boxtel, M. P. J., & Jolles, J. (2011). Computer use in older adults: Determinants and the relationship with cognitive change over a 6 year episode. *Computers in Human Behavior*, *28(1)*, 1–10. doi:10.1016/j.chb.2011.08.003

Slone, A. E., Brigham, J. C., & Meissner, C. A. (2000). Social and cognitive factors affecting the own-race bias in whites. *Basic and Applied Social Psychology*, *22(2)*, 71–84. doi:10.1207/S15324834BASP2202_1

Smeets, T., Jelicic, M., & Merckelbach, H. (2006). Stress-induced cortisol responses, sex differences, and false recollections in a DRM paradigm. *Biological Psychology*, *72*, 164–172. doi:10.1016/j.biopsycho.2005.09.004

Smith, E. E., Salat, D. H., Jeng, J., McCreary, C. R., Fischl, B., Schmahmann, J. D., . . . Greenberg, S. M. (2011). Correlations between MRI white matter lesion location and executive function and episodic memory. *Neurology*, *76*, 1492–1499. doi:10.1212/WNL.0b013e318217e7c8

Smith, S. & Fein, G. (2010). Cognitive performance in treatment-naïve active alcoholics. *Alcoholism: Clinical and Experimental Research*, *34(12)*, 2097–2105. doi:10.1111/j.1530-0277.2010.01306.x

Smits, C. H. M., Deeg, D. J. H., & Jonker, C. (1997). Cognitive and emotional predictors of disablement in older adults. *Journal of Aging and Health*, *9(2)*, 204–221.

Snitz, B. E., Loewenstein, D. A., Chang, C.-C. H., Lee, C.-W., Vander Bilt, J., Saxton, J., & Ganguli, M. (2010). A novel approach to assessing memory at the population level: Vulnerability to semantic interference. *International Psychogeriatrics*, *22(5)*, 785–794. doi:10.1017/S1041610209991657

Snitz, B. E., O'Meara, E. S., Carlson, M. C., Arnold, A. M., Ives, D. G., Rapp, S. R., . . . DeKosky, S. T. (2009). Ginkgo biloba for preventing cognitive decline in older adults. *Journal of the American Medical Association*, *302(24)*, 2663–2670. doi:10.1001/jama.2009.1913

Soares, L. M., Cachioni, M., da Silva Falcão, D. V., Tavares Batistoni, S. S., Lopes, A., Liberalesso Neri, A., & Sanches Yassuda, M. (2012). Determinants of cognitive performance among community dwelling older adults in an impoverished sub-district of São Paulo in Brazil. *Archives of Gerontology and Geriatrics*, *54*, e187–e192. doi:10.1016/j.archger.2011.11.014

Sobal, J. & Juhasz, J. B. (1977). Sex, experimenter, and reinforcement effects in verbal learning. *The Journal of*

Social Psychology, *102*, 267–273.
Söderlund, H., Nilsson, L.-G., Berger, K., Breteler, M. M., Dufouil, C., Fuhrer, R., . . . Launer, L. J. (2006). Cerebral changes on MRI and cognitive function: The CASCADE study. *Neurobiology of Aging*, *27*, 16–23. doi:10.1016/j.neurobiolaging.2004.12.008
Solowij, N., Jones, K. A., Rozman, M. E., Davis, S. M., Ciarrochi, J., Heaven, P. C. L., . . . Yücel, M. (2011). Verbal learning and memory in adolescent cannabis users, alcohol users and non-users. *Psychopharmacology*, *216*, 131–144. doi:10.1007/s00213-011-2203-x
Sommer, W., Hildebrandt, A., Kunina-Habenicht, O., Schacht, A., & Wilhelm, O. (2013). Sex differences in face cognition. *Acta Psychologia*, *142*, 62–73.
Song, X., Shang, R., Bi, Q., Zhang, X., & Wu, Y. (2012). The influence of sex difference on self-reference effects in a male-dominated culture. *Psychological Reports: Mental & Physical Health*, *111(2)*, 383–392. doi:10.2466/07.02.10.21.PR0.111.5.383-392
Sonnega, A., Faul, J. D., Ofstedal, M. B., Langa, K. M., Phillips, J. W., & Weir, D. R. (2014). Cohort profile: The Health and Retirement Study (HRS). *International Journal of Epidemiology*, 576–585. doi:10.1093/ije/dyu067
Sosa, A. L., Albanese, E., Prince, M., Acosta, D., Ferri, C. P., Guerra, M., . . . Stewart, R. (2009). Population normative data for the 10/66 Dementia Research Group cognitive test battery from Latin America, India and China: A cross-sectional survey. *BMC Neurology*, *9(48)*, 1–11. doi:10.1186/1471-2377-9-48
Spiers, M. V., Sakamoto, M., Elliott, R. J., & Baumann, S. (2008). Sex differences in spatial object-location memory in a virtual grocery store. *CyberPsychology & Behavior*, *11(4)*, 471–483. doi:10.1089/cpb.2007.0058
Squeglia, L. M., Schweinsburg, A. D., Pulido, C., & Trapert, S. F. (2011). Adolescent binge drinking linked to abnormal spatial working memory brain activation: Differential gender effects. *Alcoholism: Clinical and Experimental Research*, *35(10)*, 1831–1841. doi:10.1111/j.1530-0277.2011.01527.x
Stangor, C. (1988). Stereotype accessibility and information processing. *Personality and Social Psychology Bulletin*, *14 (4)*, 694–708.
Staresina, B. P., Bauer, H., Deecke, L., & Walla, P. (2005). Neurocognitive correlates of incidental verbal memory encoding: A magnetoencephalographic (MEG) study. *NeuroImage*, *25(2)*, 430–443. doi:10.1016/j.neuroimage.2004.11.035
Stein, J., Luppa, M., Luck, T., Maier, W., Wagner, M., Daerr, M., . . . Riedel-Heller, S. G. (2012). The assessment of changes in cognitive functioning: Age-, education-, and gender-specific reliable change indices for older adults tested on the CERAD-NP battery: Results of the German Study on Ageing, Cognition, and Dementia in Primary Care Patients (AgeCoDe). *Amrican Journal of Geratric Psychiatry*, *20:1*, 84–97. doi:10.1097/JGP.0b013e318209dd08
Steptoe, A., Breeze, E., Banks, J., & Nazroo, J. (2013). Cohort profile: The English Longitudinal Study of Ageing. *International Journal of Epidemiology*, *42*, 1640–1648. doi:10.1093/ije/dys168
Stewart, R., Richards, M., Brayne, C., & Mann, A. (2001). Cognitive function in UK-community dwelling African Caribbean elders: Normative data for a test battery. *International Journal of Geriatric Psychiatry*, *16(5)*, 518–527. doi:10.1002/gps.384
Stijntjes, M., de Craen, A. J. M., van Heemst, D., Meskers, C. G. M., van Buchem, M. A., Westendorp, R. G. J., . . . Maier, A. B. (2013). Familial longevity is marked by better cognitive performance at middle age: The Leiden Longevity Study. *PLoS ONE*, *8(3)*, 1–8. doi:10.1371/journal.pone.0057962
Stumpf, H. (1998). Gender-related differences in academically talented students' scores and use of time on tests of spatial memory. *Gifted Child Quarterly*, *42 (3)*, 157–171.
Stumpf, H. & Jackson, D. N. (1994). Gender-related differences in cognitive abilities: Evidence from a medical school admissions testing program. *Personality and Individual Differences*, *17(3)*, 335–344.
Su, C.-Y., Chen, H.-M., Kwan, A.-L., Lin, Y.-H., & Guo, N.-W. (2007). Neuropsychological impairment after hemorrhagic stroke in basal ganglia. *Archives of Clinical Neuropsychology*, *22*, 465–474.

doi:10.1016/j.acn.2007.01.025

Sunderaraman, P., Blumen, H. M., DeMatteo, D., & Cosentino, S. (2013). Task demand influences relationships among sex, clustering strategy, and recall: 16-word versus 9-word list learning tests. *Cognitive Behavioral Science*, *26*, 78–84. doi:10.1097/WNN.0b013e31829de450

Sung, Y. H. & Dawis, R. V. (1981). Level and factor structure differences in selected abilities across race and sex groups. *Journal of Applied Psychology*, *66(5)*, 613–624.

Susilo, T., Germine, L., & Duchaine, B. (2013). Face recognition ability matures late: Evidence from individual differences in young adults. *Journal of Experimental Psychology*, *39(5)*, 1212–1217. doi:10.1037/a0033469

Swan, G. E., Lessov-Schlaggar, C. N., Carmelli, D., Schellenberg, G. D., & La Rue, A. (2005). Apolipoprotein E ε4 and change in cognitive functioning in community-dwelling older adults. *Journal of Geriatric Psychiatry and Neurology*, *18*, 196–201. doi:10.1177/0891988705281864

Takei, K., Yamasue, H., Abe, O., Yamada, H., Inoue, H., Suga, M., . . . Kasai, K. (2009). Structural disruption of the dorsal cingulum bundle is associated with impaired Stroop performance in patients with schizophrenia. *Schizophrenia Research*, *114*, 119–127. doi:10.1016/j.schres.2009.05.012

Tamm, L., Epstein, J. N., Lisdahl, K. M., Molina, B., Tapert, S., Hinshaw, S. P., . . . Swanson, J. M. (2013). Impact of ADHD and cannabis use on executive functioning in young adults. *Drug and Alcohol Dependence*, *133*, 607–614. doi:10.1016/j.drugalcdep.2013.08.001

Temple, C. M. & Cornish, K. M. (1993). Recognition memory for words and faces in schoolchildren: A female advantage for words. *British Journal of Developmental Psychology*, *11*, 421–426.

Terry, D. P., Puente, A. N., Brown, C. L., Faraco, C. C., & Miller, L. S. (2013). Openness to experience is related to better memory ability in older adults with questionable dementia. *Journal of Clinical and Experimental Neuropsychology*, *35(5)*, 509–517. doi:10.1080/13803395.2013.795932

Thakur, G. P., Shahi, S. P., & Kumar, A. (1981). Social memory task in relation to sex, age, and rural-urban background. *Psychological Reports*, *49*, 948.

Thomson, P. A., Harris, S. E., Starr, J. M., Whalley, L. J., Porteous, D. J., & Deary, I. J. (2005). Association between genotype at an exonic SNP in DISC1 and normal cognitive aging. *Neuroscience Letters*, *389*, 41–45. doi:10.1016/j.neulet.2005.07.004

Timothy, S. A. (2014). Correlates of cognitive change. *Journal of Experimental Psychology: General*, *143(3)*, 1026–1048. doi:10.1037/a0034847

Tippet, W. J., Lee, J. H., Mraz, R., Zakzanis, K. K., Snyder, P. J., Black, S. A., & Graham, S. J. (2009). Convergent validity and sex differences in healthy elderly adults for performance on 3D virtual reality navigation learning and 2D hidden maze tasks. *CyberPsychology & Behavior*, *12*, 169–174. doi:10.1089/cpb.2008.0218

Toomela, A. (2012). Short-term memory in young adults with spastic diplegic cerebral palsy. *Developmental Neuropsychology*, *37(4)*, 317–332. doi:10.1080/87565641.2011.632461

Torniainen, M., Suviaari, J., Partonen, T., Castaneda, A. E., Kuha, A., Perälä, J., . . . Annamari, T.-H. (2011). Sex differences in cognition among persons with schizophrenia and healthy first-degree relatives. *Psychiatry Research*, *188*, 7–12. doi:10.1016/j.psychres.2010.11.009

Torrent, C., Martinez-Arán, A., Daban, C., Amann, B., Balanzá-Martínez, V., del Mar Bonnín, C., . . . Vieta, E. (2011). Effects of atypical antipsychotics on neurocognition in euthymic bipolar patients. *Comprehensive Psychiatry*, *52*, 613–622. doi:10.1016/j.comppsych.2010.12.009

Torres, D. S., Abrantes, J., & Brandão-Mello, C. E. (2013). Cognitive assessment of patients with minimal hepatic encephalopathy in Brazil. *Metabolic Brain Disease*, *28(3)*, 473–483. doi:10.1007/s11011-013-9405-3

Tottenham, L. S., Deboran, S., Elias, L., & Gutwin, C. (2003). Female advantage for spatial location memory in both static and dynamic environments. *Brain and Cognition*, *53*, 381–383. doi:10.1016/S0278-2626(03)00149-0

Townes, B. D., Martins, P. I., Castro-Caldas, I., Rosenbaum, G., & Derouen, T. (2008). Repeat test scores on

neurobehavioral measures over an eight-year period in a sample of Portuguese children. *International Journal of Neuoscience*, *118*, 79–93. doi:10.1080/00207450601042102

Trachtenberg, R. E., Aisen, P. S., & Chuang, Y.-L. (2005). One-trial 10-item free-recall performance in Taiwanese elderly and near-elderly: A potential screen for cognitive decline. *American Journal of Alzheimer's Disease and Other Dementias*, *20(4)*, 239–247. doi:10.1177/153331750502000410

Trahan, D. E. & Quintana, J. W. (1990). Analysis of gender effects upon verbal and visual memory performance in adults. *Archives of Clinical Neuropsychology*, *5*, 325–334.

Tropp Sneider, J., Sava, S., Rogowska, J., & Yurgelun-Todd, D. A. (2011). A preliminary study of sex differences in brain activation during a spatial navigation task in healthy adults. *Perceptual and Motor Skills*, *113(2)*, 461–480. doi:10.2466/04.22.24.27.PMS.113.5.461-480

Unsworth, N. (2010). On the division of working memory and long-term memory and their relation to intelligence: A latent variable approach. *Acta Psychologica*, *134(1)*, 16–28. doi:10.1016/j.actpsy.2009.11.010

Unterhalter, G., Farrell, S., & Mohr, C. (2007). Selective memory biases for words reflecting sex-specific body image concerns. *Eating Behaviors*, *8(3)*, 382–389. doi:10.1016/j.eatbeh.2006.11.015

Unwerzagt, F. W., Ogunniyi, A., Taler, V., Gao, S., Lane, K. A., Baiyewu, O., . . . Hall, K. S. (2011). Incidence and risk factors for cognitive impairment no dementia and mild cognitive impairment in African Americans. *Alzheimer Disease & Associated Disorders*, *25(1)*, 4–10. doi:10.1097/WAD.0b013e3181f1c8b1

Uttl, B., Graf, P., & Richter, L. K. (2002). Verbal paired associates tests limits on validity and reliability. *Archives of Clinical Neuropsychology*, *17(6)*, 567–581. doi:10.1016/S0887-6177(01)00135-4

Uttner, I., Weber, S., Freund, W., Bengel, D., Schmitz, D., Ludolph, A. C., & Huber, R. (2011). Hippocampal cavities are not associated with cognitive impairment in transient global amnesia. *European Journal of Neurology*, *18*, 882–887. doi:10.1111/j.1468-1331.2010.03310.x

Vakil, E. & Blachstein, H. (1994). A supplementary measure in the Rey AVLT for assessing incidental learning of temporal order. *Journal of Clinical Psychology*, *50(2)*, 240–245.

Vakil, E. & Blachstein, H. (1997). Rey AVLT: Developmental norms for adults and the sensitivity of different memory measures to age. *The Clinical Neuropsychologist*, *11(4)*, 356–369.

Vakil, E., Greenstein, Y., & Blachstein, H. (2010). Normative data for composite scores for children and adults derived from the Rey Auditory Verbal Learning Test. *The Clinical Neuropsychologist*, *24(4)*, 662–677. doi:10.1080/13854040903493522

Valis, M., Masopust, J., Bazant, J., Rihova, Z., Kalnicka, D., Urban, A., . . . Hort, J. (2011). Cognitive changes in spinocerebellar ataxia type 2. *Neuroendocrinology Letters*, *32(3)*, 354–359.

van der Werf, M., Köhler, S., Verkaaik, M., Verhey, F., & van Os, J. (2012). Cognitive functioning and age at onset in non-affective psychotic disorder. *Acta Psychiatrica Scandinavia*, *126(4)*, 274–281. doi:10.1111/j.1600-0447.2012.01873.x

van Boxtel, M. P. J., Langerak, K., Houx, P. J., & Jolles, J. (1996). Self-reported physical activity, subjective health, and cognitive performance in older adults. *Experimental Aging Research*, *22(4)*, 363–379.

van Exel, E., Gussekloo, J., Houx, P., de Craen, A. J. M., Macfarlane, P. W., Bootsma-van der Wiel, A., . . . Westendorp, R. J. J. (2002). Atherosclerosis and cognitive impairment are linked in the elderly. the Leiden 85-plus Study. *Atherosclerosis*, *165*, 353–359.

van Hooren, S. A. H., Valentijn, A. M., Bosma, H., Ponds, R. W. H. M., van Boxtel, M. P. J., & Jolles, J. (2007). Cognitive functioning in healthy older adults aged 64-81: A cohort study into the effects of age, sex and education. *Aging, Neuropsychology and Cognition*, *14(1)*, 40–54. doi:10.1080/138255890969483

van Oostrom, I., Franke, B., Rijpkema, M., Gerritsen, L., Arias-Vasquez, A., Fernandez, G., & Tendolkar, I. (2012). Interaction between BDNF Val66Met and childhood stressful life events is associated to affective memory bias in men but not women. *Biological Psychology*, *89(1)*, 214–219. doi:10.1016/j.biopsycho.2011.10.012

Vanhoutte, S., de Letter, M., Corthals, P., van Borsel, J., & Santens, P. (2012). Quantitative analysis of language production in Parkinson's disease using a cued sentence generation task. *Clinical Linguistics & Phonetics*,

26(10), 863–881. doi:10.3109/02699206.2012.711420
Vaskinn, A., Sundet, K., Simonsen, C., Hellvin, T., Melle, I., & Andreassen, O. A. (2011). Sex differences in neuropsychological performance and social functioning in schizophrenia and bipolar disorder. *Neuropsychology*, *25 (4)*, 449–510. doi:10.1037/a0022677
Veena, S. R., Hegde, B. G., Ramachandraiah, S., Krishnaveni, G. V., Fall, C. H. D., & Krishnamachari, S. (2014). Relationship between adiposity and cognitive performance in 9-10-year-old children in south India. *Archives of Disease in Childhood*, *99(2)*, 126–34. doi:10.1136/archdischild-2013-304478
Veena, S. R., Krishnaveni, G. V., Srinivasan, K., Kurpad, A. V., Muthayya, S., Hill, J. C., . . . Fall, C. H. D. (2010). Childhood cognitive ability: Relationship to gestational diabetes mellitus in India. *Diabetologia*, *53*, 2134–2138. doi:10.1007/s00125-010-1847-0
Venter, A. & Louw, D. A. (2004). The effect of violent versus non-violent incidents on eyewitness memory. *Medicine and Law*, *23(4)*, 833–58.
Venter, A. & Low, D. A. (2005). Method of testing and the accuracy of eyewitness testimony. *Medicine and Law*, *24*, 61–79.
Verbaam, D., Marinus, J., Visser, M., van Rooden, S. M., Stiggelbout, A. M., Middelkoop, H. A. M., & van Hilten, J. J. (2007). Cognitive impairment in Parkinson's disease. *Journal of Neurology, Neurosurgery & Psychiatry*, *78(11)*, 1182–1187. doi:10.1136/jnnp.2006.112367
Vilberg, K. L. & Rugg, M. D. (2012). The neural correlates of recollection: Transient versus sustained fMRI effects. *The Journal of Neuroscience*, *32(45)*, 15679–15687. doi:10.1523/JNEUROSCI.3065-12.2012
Villardita, C., Smirni, P., Le Pira, F., & Zappalà, G. (1981). Verbal, visual, and spatial learning in adolesence: Sex differences. *Italian Journal of Psychology*, *8(2)*, 81–85.
Vitulli, W. F. & Henderson, M. E. (1994). Size of audience, gender, and digitrate effects on short-term memory. *Psychological reports*, *74*, 315–322.
Volf, N. V. & Razumnikova, O. M. (2001). Gender differences in hemispheric spatiotemporal EEG patterns upon reproduction of verbal information. *Human Pysiology*, *30(3)*, 274–280.
Volz-Sidiropoulou, E. & Gauggel, S. (2011). Do subjective measures of attention and memory predict actual performance? Metacognition in older couples. *Psychology and Aging*, *27(2)*, 440–450. doi:10.1037/a0025384
Wagner, M., Schulze-Rauschenbach, S., Petrovsky, N., Brinkmeyer, J., von der Goltz, C., Grunder, G., . . . Winterer, G. (2012). Neurocognitive impairments in non-deprived smokers-results from a population-based multi-center study on smoking-related behavior. *Addiction Biology*, *18*, 752–761. doi:10.1111/j.1369-1600.2011.00429.x
Wagovich, S. A., Pak, Y., & Miller, M. D. (2012). Orthographic word knowledge growth in school-age children. *American Journal of Speech-Language Pathology*, *21*, 140–153. doi:10.1044/1058-0360(2012/10-0032)
Wahlin, Å., deFrias, C. M., MacDonald, S. W. S., Nilsson, L., & Dixon, R. A. (2006). How do health and biological age influence chronological age and sex differences in cognitive aging: Moderating, mediating, or both? *Psychology and Aging*, *21(2)*, 318–332. doi:10.1037/0882-7974.21.2.318
Waldstein, S. R. & I., K. L. (2004). Gender differences in the relation of hypertension to cognitive function in older adults. *Neurological Research*, *26(5)*, 502–506. doi:10.1179/016164104225016173
Walhovd, K. B., Fjell, A. M., Dale, A. M., Fischl, B., Quinn, B. T., Makris, N., . . . Reinvang, I. (2006). Regional cortical thickness matters in recall after months more than minutes. *NeuroImage*, *31*, 1343–1351. doi:10.1016/j.neuroimage.2006.01.011
Wang, B. (2012a). Effect of positive emotion on consolidation of memory for faces: The modulation of facial valence and facial gender. *Memory*, *21(6)*, 707–721. doi:10.1080/09658211.2012.753461
Wang, B. (2012b). Females' superiority in item memory, but not source memory for neutral and emotional Chinese words. *Personality and Individual Differences*, *52(8)*, 925–929. doi:10.1016/j.paid.2012.02.005
Wang, B. & Fu, X.-l. (2009). Gender difference in the effect of daytime sleep on declarative memory for pictures. *Journal of Zhejiang University-SCIENCE B*, *10(7)*, 536–546. doi:10.1631/jzus.B0820384

Wang, B. & Fu, X. (2010). Gender differences in the effects of post-learning emotion on consolidation of item memory and source memory. *Neurobiology of Learning and Memory*, *93(4)*, 572–580. doi:10.1016/j.nlm.2010.02.005

Wang, B. (2013). Gender difference in recognition memory for neutral and emotional faces. *Memory*, *21(8)*, 991–1003. doi:10.1080/09658211.2013.771273

Wang, H.-B., Ma, N., Yu, Y.-Q., Chen, Y.-R., Wang, K., & Zhang, D.-R. (2010). Is the contribution of the amygdala to the sex- and enhancement-related effects of emotional memory time-dependent? *Neurobiology of Learning and Memory*, *93*, 1–7. doi:10.1016/j.nlm.2009.07.009

Wang, H.-X., Jin, Y., Hendrie, H. C., Liang, C., Yang, L., Cheng, Y., . . . Gao, S. (2013). Late life leisure activities and risk of cognitive decline. *Journals of Gerontology Series A: Biological Sciences and Medical Sciences*, *68(2)*, 205–213. doi:10.1093/gerona/gls153

Weintraub, S., Salmon, D., Mercaldo, N., Ferris, S., Graff-Radford, N. R., Chui, H., . . . Morris, J. C. (2009). The Alzheimer's disease centers' Uniform Data Set (UDS): The neuropsychological test battery. *Alzheimer Disease & Associated Disorders*, *23(2)*, 91–101. doi:10.1097/WAD.0b013e318191c7dd

Weirich, S., Hoffmann, F., Meissner, L., Heinz, A., & Bengner, T. (2011). Sex influence on face recognition memory moderated by presentation duration and reencoding. *Neuropsychology*, *25(6)*, 806–813. doi:10.1037/a0025633

Weiss, E. M., Ragland, J. D., Brensinger, C. M., Bilker, W. B., Deisenhammer, E. A., & Delazer, M. (2006). Sex differences in clustering and switching in verbal fluency tasks. *Journal of the International Neuropsychological Society*, *12*, 502–509. doi:10.1017/S1355617706060656

Wesson Ashford, J., Gere, E., & Bayley, P. J. (2014). Measuring memory in large group settings using a continuous recognition test. *Journal of Alzheimer's Disease*, *27*, 885–895. doi:10.3233/JAD-2011-110950

West, R. L., Crook, T. L., & Barron, K. L. (1992). Everyday memory performance across the life span: Effects of age and noncognitive individual differences. *Psychology and Aging*, *7(1)*, 72–82.

West, R. L., Welch, D. C., & Knabb, P. D. (2002). Gender and aging: Spatial self-efficacy and location recall. *Basic and Applied Social Psychology*, *24(1)*, 71–80. doi:10.1207/S15324834BASP2401_7

Wicks, P., Abrahams, S., Papps, B., Al-Chalabi, A., Shaw, C. E., Leigh, P. N., & Goldstein, L. H. (2009). SOD1 and cognitive dysfunction in familial amyotrophic lateral sclerosis. *Journal of Neurology*, *256*, 234–241. doi:10.1007/s00415-009-0078-0

Widmann, C. N., Beinhoff, U., & Riepe, M. W. (2012). Everyday memory deficits in very mild Alzheimer's disease. *Neurobiology of Aging*, *33(2)*, 297–303. doi:10.1016/j.neurobiolaging.2010.03.012

Wiebe, M. J. & Watkins, E. O. (1980). Factor analysis of the McCarthy scales of children's abilities on preschool children. *Journal of School Psychology*, *18(2)*, 154–162. doi:10.1016/0022-4405(80)90031-X

Wiederholt, W. C., Cahn, D., Butters, N. M., Salmon, D. P., Kritz-Silverstein, D., & Barrett-Connor, E. (1993). Effects of age, gender and education on selected neuropsychological tests in an elderly community cohort. *Journal of the American Geriatrics Society*, *41(6)*, 639–647.

Wilhelm, P. & Van Klink, M. (2007). Validity of the Rey Visual Design Learning Test in primary and secondary school children. *Child Neuropsychology: A Journal on Normal and Abnormal Developent in Childhood and Adolescence*, *13/1*, 86–98. doi:10.1080/09297040600634579

Wingbermühle, E., Roelofs, R. L., van der Burgt, I., Souren, P. M., Verhoeven, W. M. A., Kessels, R. P. C., & Egger, J. I. M. (2012). Cognitive functioning of adults with Noonan syndrome: A case-control study. *Genes, Brain and Behavior*, *11(7)*, 785–793. doi:10.1111/j.1601-183X.2012.00821.x

Woicik, P. A., Moeller, S. J., Alia-Klein, N., Maloney, T., M., L. T., Yeliosof, O., . . . Goldstein, R. Z. (2009). The neuropsyhology of cocaine addiction: Recent cocaine use masks impairment. *Neuropsychopharmacology*, *34(5)*, 1112–1122. doi:10.1038/npp.2008.60

Wolf, O. T., Schommer, N. C., Hellhammer, D. H., McEwen, B. S., & Kirschbaum, C. (2001). The relationship between stress induced cortisol levels and memory differs between men and women. *Psychoneuroendocrinology*, *26*, 711–720.

Wolff, N., Kemter, K., Schweinberger, S. R., & Wiese, H. (2013). What drives social in-group biases in face recognition memory? ERP evidence from the own-gender bias. *Social Cognitive and Affective Neuroscience*, *9(5)*, 580–590. doi:10.1093/scan/nst024

Woo, S. J., Park, K. H., Ahn, J., Choe, J. Y., Jeong, H., Han, J. W., . . . Kim, K. W. (2012). Cognitive impairment in age-related macular degeneration and geographic atrophy. *Ophthalmology*, *119(10)*, 2094–2101. doi:10.1016/j.ophtha.2012.04.026

Woolley, D. G., Vermaercke, B., Op de Beeck, H., Wagemans, J., Gantois, I., D'Hooge, R., . . . Wenderoth, N. (2010). Sex differences in human virtual water maze performance: Novel measures reveal the relative contribution of directional responding and spatial knowledge. *Behavioural Brain Research*, *208*, 408–414. doi:10.1016/j.bbr.2009.12.019

Xu, L., Jiang, C. Q., Lam, T. H., Liu, B., Jin, Y. L., Zhu, T., . . . Thomas, N. (2011). Short or long sleep duration is associated with memory impairment in older Chinese: The Guangzhou Biobank Cohort Study. *Sleep*, *34(5)*, 575–580.

Yang Zhang, X., Chun Chen, D., Hong Xiu, M., De Yang, F., Haile, C. N., Kosten, T. A., & Kosten, T. R. (2012). Gender differences in never-medicated first-episode schizophrenia and medicated chronic schizophrenia patients. *Journal of Clinical Psychiatry*, *73(7)*, 1025–1033. doi:10.4088/JCP.11m07422

Yang, L., Unverzagt, F. W., Jin, Y., Hendrie, H. C., Liang, C., Hall, K. S., . . . Gao, S. (2012). Normative data for neuropsychological tests in a rural elderly Chinses cohort. *The Clinical Neuropsychologist*, *26(4)*, 641–653. doi:10.1080/13854046.2012.666266

Ye, B. S., Seo, S. W., Lee, Y., Kim, S. Y., Choi, S. H., Lee, Y. M., . . . Kim, E. (2012). Neuropsychological performance and conversion to Alzheimer's disease in early- compared to late-onset amnestic mild cognitive impairment: CREDOS study. *Dementia and Geriatric Cognitive Disorders*, *34*, 156–166. doi:10.1159/000342973

Young, G. D. & Wilson, J. F. (1994). Comparing matching ability, spatial memory and ideational fluency in boys and girls. *Perceptual and Motor Skills*, *79(2)*, 1019–1024. doi:10.2466/pms.1994.79.2.1019

Young, R. J. (1979). The effect of regular exercise on cognitive functioning and personality. *British Journal of Sports Medicine*, *13(3)*, 110–117.

Youngjohn, J. R., Larrabe, G. J., & Crook, T. H. (1991). First-Last Names and the Grocery List Selective Reminding Test: Two computerized measures of everyday verbal learning. *Archives of Clinical Neuropsychology*, *6(4)*, 287–300.

Ystad, M. A., Lundervold, A. J., Wehling, E., Espeseth, T., Rootwelt, H., Westlye, L. T., . . . Lundervold, A. (2009). Hippocampal volumes are important predictors for memory function in elderly women. *Medical Imaging*, *9(17)*, 1–15. doi:10.1186/1471-2342-9-17

Yurgelun-Todd, D. A., Killgore, W. D. S., & Cintron, C. B. (2003). Cognitive correlates of medial temporal lobe development across adolescence: A magnetic resonance imaging study. *Perceptual and Motor Skills*, *96(1)*, 3–17. doi:10.2466/pms.2003.96.1.3

Zahodne, L. B. S., Farrell, P. W., Stern, M. T., & Manly, J. J. (2014). Bilingualism does not alter cognitive decline or dementia risk among Spanish-speaking immigrants. *Neuropsychology*, *28(2)*, 248–246. doi:10.1037/neu0000014

Zanello, A., Perrig, L., & Huguelet, P. (2006). Cognitive functions related to interpersonal problem-solving skills in schizophrenic patients compared with health subjects. *Psychiatry Research*, *142(1)*, 67–78. doi:10.1016/j.psychres.2003.07.009

Zehnder, A. E., Bläsi, S., Berres, M., Monsch, A. U., Stähelin, H. B., & Spiegel, R. (2009). Impact of APOE status on cognitive maintenance in healthy elderly persons. *International Journal of Geriatric Psychiatry*, *24*, 132–141. doi:10.1002/gps.2080

Zhang, H., Wang, X., Lin, J., Sun, Y., Huang, Y., Yang, T., . . . Zhang, J. (2012). Grey and white matter abnormalities in chronic obstructive pulmonary disease: A case-control study. *BMJ Open*, *2(2)*, e000844. doi:10.1136/bmjopen-2012-000844

Zhong, W., Cruickshanks, K. J., Schubert, C. R., Carlsson, R. J., C. M. Chappel, Klein, B. E. K., Klein, R., & Acher, C. W. (2014). Pulse wave velocity and cognitive functions in older adults. *Alzheimer Disease & Associated Disorders*, *28(1)*, 44–49. doi:10.1097/WAD.0b013e3182949f06

Zimmerman, M. E., Bigal, M. E., Katz, M. J., Brickman, A. M., & Lipton, R. B. (2012). Sleep onset/maintenance difficulties and cognitive function in nondemented older adults: The role of cognitive reserve. *Journal of International Neuropsychological Society*, *18*, 260–470. doi:10.1017/S1355617711001901

Zoladz, P. R., Warnecke, A. J., Woelke, S. A., Burke, H. M., Frigo, R. M., Pisansky, J. M., . . . Talbot, J. N. (2013). Pre-learning stress that is temporally removed from acquisition exerts sex-specific effects on long-term memory. *Neurobiology of Learning and Memory*, *100*, 77–87. doi:10.1016/j.nlm.2012.12.012
